# Clinically Significant Cytochrome P450-Mediated Drug-Drug Interactions in Children Admitted to Intensive Care Units

**DOI:** 10.1155/2022/2786914

**Published:** 2022-08-23

**Authors:** Tong Li, Biwen Hu, Ling Ye, Zeying Feng, Longjian Huang, Chengjun Guo, Xiong Wu, Wei Tan, Yi Wang, Guoping Yang, Chengxian Guo

**Affiliations:** ^1^Center of Clinical Pharmacology, The Third Xiangya Hospital, Central South University, Changsha 410013, Hunan, China; ^2^Youjiang Medical University for Nationalities, Baise 533000, Guangxi, China; ^3^School of Applied Mathematics, Guangdong University of Technology, Guangzhou 510006, Guangdong, China; ^4^Easier Data Technologies Co., Ltd, Changsha 410016, China; ^5^Department of Neonatology, Maternal& Child Health Hospital of Guangxi Zhuang Autonomous Region, Nanning 53003, Guangxi Zhuang Autonomous Region, China

## Abstract

**Objectives:**

Children admitted to intensive care units (ICUs) often require multiple medications due to the complexity and severity of their disease, which put them at an increased risk for drug interactions. This study examined cytochrome P450-mediated drug-drug interactions (DDIs) based on the Pediatric Intensive Care (PIC) database, with the aim of analyzing the incidence of clinically significant potential drug-drug interactions (pDDIs) and exploring the occurrence of actual adverse reactions.

**Methods:**

The Lexicomp database was used to screen cytochrome P450-mediated DDI pairings with good levels of reliability and clear clinical phenotypes. Patients exposed to the above drug pairs during the same period were screened in the PIC database. The incidence of clinically significant pDDIs was calculated, and the occurrence of adverse reactions was explored based on laboratory measurements.

**Results:**

In total, 84 (1.21%) of 6920 children who used two or more drugs were exposed to at least one clinically significant pDDI. All pDDIs were based on CYP3A4, with nifedipine + voriconazole (39.60%) being the most common drug pair, and the most frequent being the J02 class of drugs. Based on laboratory measurements, 15 adverse reactions were identified in 12 patients.

**Conclusions:**

Clinically significant cytochrome P450-mediated pDDIs existed in the children admitted to ICUs, and some of the pDDIs led to adverse clinical outcomes. The use of clinical decision support systems can guide clinical medication use, and clinical monitoring of patients' needs has to be enhanced.

## 1. Introduction

Children admitted to intensive care units (ICUs) often suffer from severe, complex medical conditions that expose them to multiple medications [1]. Many studies have shown that simultaneous use of multiple drugs increases the risk of potential drug-drug interactions (pDDIs) [2–4]. Drug-drug interactions (DDIs) are common and preventable prescribing errors. According to the US Food and Drug Administration (FDA), DDIs refer to the phenomenon that the effects and duration of drugs are changed to varying degrees due to drug interactions when two or more drugs are used simultaneously or sequentially [5].

DDIs are generally classified as pharmacokinetic and pharmacodynamic interactions. Pharmacokinetic interactions can occur during the absorption, distribution, metabolism, and excretion phases, with cytochrome P450 (CYP450)-mediated interactions during the drug metabolism phase being the most common and preventable drug interactions. Current studies have shown that most DDIs have adverse effects on patient care, potentially reducing drug efficacy or enhancing drug toxicity, causing treatment failure, adverse drug events, and even death [6]. In children, their drug metabolism in the liver is low maturity and many CYP450 enzymes are expressed at low levels, which may mean that children admitted to ICUs are more susceptible to adverse effects of CYP-mediated DDIs [7]. However, there is literature on the prevalence of CYP-mediated drug interactions in elderly patients [8, 9]and psychiatric patients [10, 11], but there is no information on CYP-mediated pDDIs in children.

Although pDDIs are important causes of adverse drug reactions (ADRs), not all pDDIs are clinically significant, and identifying the incidence of clinically significant pDDIs is even more important for children in ICUs [12], which can help clinicians or pharmacists identify drug combinations that need to be avoided [13]. However, there are situations where certain drugs must be used together for therapeutic purposes even though they may interact with each other. Assessing the occurrence of pDDI-related adverse reactions in such cases can prompt physicians to monitor patients for serum drug concentrations and adverse reactions to avoid the adverse consequences of drug interactions whenever possible.

There have been some studies on DDIs of children in ICUs [4, 12, 14, 15]. The incidence of pDDIs has been found to be related to the number of drugs used, and pDDIs can increase the length of stay. However, the occurrence of clinically significant CYP-mediated pDDIs in children is poorly studied, and the related adverse effects have not been investigated. Therefore, in this study, we aimed to assess the prevalence of clinically significant CYP450-mediated pDDIs in children admitted to ICUs using medication information from the Pediatric Intensive Care (PIC) database and to evaluate the incidence of the actual adverse reactions based on laboratory test data. The complexity of medications in the ICUs may lead to an increased incidence of pDDIs, and the unique nature of the children may expose them to higher risks of associated adverse reactions. Increased understanding of DDIs of children in the ICUs can help improve the safety of drug prescriptions and provide guidance for clinical monitoring, thus improving ICU children's care.

## 2. Methods

### 2.1. Data Sources

This retrospective study was conducted using the patient's data from the PIC database [16], which contains information of patients admitted to the Children's Hospital of Zhejiang University School of Medicine (Zhejiang, China) between 2010 and 2019. The database includes demographic information, length of hospital stay, vital sign measurements, laboratory measurements, diagnoses, medications, and survival data.

### 2.2. Eligibility Criteria and Study Population

The drug information in the PIC database contains the approved drug names, the time and mode of administration, and the dose. The medication information in the database was initially cleaned to exclude the following medications: (1) topical medications such as creams and drops; (2) Chinese herbal medicines; (3) glucose injection and sodium chloride injection series. Patients aged 0–17 years and who took two or more medications (after cleaning) during hospitalization were screened for further study.

### 2.3. Definitions of CYP-Mediated pDDIs

The CYP-mediated pDDI pairings with good levels of reliability and clear clinical phenotypes were selected for this study. Potential pDDI pairings were identified using the information provided in the Lexicomp database (an online drug interaction checker, https://www.uptodate.com/drug-interactions), which classifies pDDIs into 6 reliability ratings, from low to high. In this study, we selected 3 high levels: reliability rating fair, reported in the prescribing information, reliability rating good, and reliability rating excellent. The database also gives patient management recommendations, and we selected pDDI pairings with clear clinical phenotypes (with clear clinical management recommendations) for further study (see in supplementary table).

### 2.4. Identification of Clinically Significant CYP-Mediated pDDIs

In this study, clinically significant CYP-mediated pDDI was defined as exposure to two drugs of the above pDDI pairings during the 24 h period of hospitalization. This criterion was used to identify the occurrence of clinically significant pDDIs in all included patients.

### 2.5. Identification of Adverse Reactions

Criteria for identifying adverse reactions based on laboratory test results: laboratory test results were normal at the 1st test and abnormal at the nth test, and patients were exposed to the DDI pairings within 7 days before the abnormality [17]. The abnormal values were determined according to the reference literature or relevant treatment guidelines. The diagnostic criteria for adverse reactions based on laboratory test results used in this study are shown as supplementary methods.

## 3. Results

### 3.1. Clinical and Demographic Characteristics of Patients

A total of 6920 patients in the PIC database used at least two drugs during their hospitalization, ranging from 2 to 104 types. Of these patients, 84 (1.21%) were exposed to clinically significant CYP-mediated pDDIs (Table 1), and their ages ranged from 0 to 14 years (median age of 4 years). The patients' common diagnoses were diseases of the respiratory system (20, 23.81%), neoplasms (16, 19.05%), and certain conditions originating in the perinatal period (12, 14.29%). The length of stay ranged from 6 to 335 days, with a median length of 37.5 days. During the period, the minimum type of medication was 23, the maximum was 103, and the median was 45.

### 3.2. Prevalence and Pattern of Clinically Significant CYP-Mediated pDDIs

**Figure 1:**
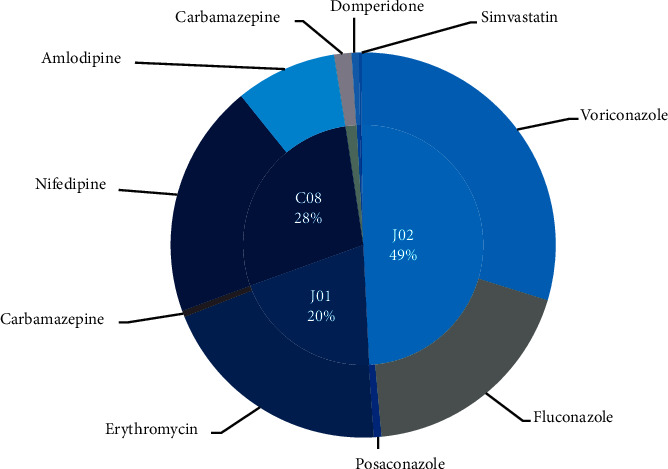
Drugs involved in pDDIs. −pDDIs = potential drug-drug interactions; −ATC = anatomical therapeutic chemical; −J02, J01, and C08 are ATC codes.

In total, 84 patients were exposed to 101 interactions, involving 8 pDDI pairings (Table 2). Nifedipine + voriconazole, erythromycin + fluconazole, and amlodipine + voriconazole were the most common combinations causing pDDIs. All pDDIs were based on CYP3A4 metabolism and were a combination of substrate and inhibitor. All the pDDIs had good reliability ratings. The 10 drugs involved were classified according to ATC codes (Anatomical Therapeutic Chemical) (Table 3, Figure 1), and the most frequent categories were J02 (antimycotics for systemic use, 49%), C08 (calcium channel blockers, 28%), and J01 (antibacterials for systemic use, 20%). The most prevalent drugs were voriconazole (29.70%), nifedipine (19.80%), erythromycin (19.80%), and fluconazole (18.81%).

### 3.3. Occurrence of Adverse Reactions Based on Laboratory Test Results

A total of 12 (14.29%) of the 84 children had 15 adverse reactions (Table 4), of which 4 were rhabdomyolyses, 4 leukopenia, 3 neutropenia, 2 acute kidney injury, 1 myocardial injury, and 1 thrombocytopenia. The most frequently occurring DDI pairing that caused adverse reactions was nifedipine + voriconazole (10 times).

## 4. Discussion

In this study, we identified the prevalence and characteristics of CYP-mediated and clinically significant pDDIs in ICU hospitalized children from the PIC database and identified the occurrence of adverse reactions based on laboratory test results. There were several studies on the prevalence, common drug pairs, risk factors, and adverse outcomes of pDDIs in children in ICUs [4, 12, 14, 15, 18]. However, the occurrence of CYP-mediated and clinically significant pDDIs and related adverse effects have not been studied to the best of our knowledge.

Our study found that 84 (1.21%) of 6920 children who used more than two drugs were exposed to at least one clinically significant CYP-mediated pDDI. The pDDIs identified in our study involved a total of 8 pDDI pairings, with nifedipine + voriconazole (39.60%) and erythromycin + fluconazole (33.66%) being the two most common drug combinations, accounting for more than 70% of all drug pairs. In our study, we only focused on pDDIs based on CYP450 with good reliability levels and clinical significance. But most studies examined all types of pDDIs, which may also include pharmacodynamics, other types of pharmacokinetics, and interactions of unclear clinical significance. Thus, our study showed a low incidence of pDDIs and the pDDI pairings with a high incidence found in this study as well as commonly used drugs were also inconsistent with other studies.

The pDDIs we identified were all CYP3A4 involved. Human cytochrome CYP3A4 is the most abundant hepatic and intestinal phase I enzyme, metabolizing about 50% of the drugs [19]. In humans, CYP3A4 shows an age-dependent maturation pattern [20], which allows for possible differences in CYP3A4-mediated drug metabolism between children and adults.

In addition, the 10 drugs involved were categorized using ATC codes, and the most frequent category of occurrence was J (anti-infectives for systemic use). It was consistent with the most frequently reported drug categories that led to ADR visits in previous studies [21]. However, the most frequent drugs in our study were voriconazole (29.70%) and erythromycin (19.80%), which may be due to their common clinical use as antifungal drugs [22] and also as effective inhibitors of CYP3A4. The concomitant use of substrates and inhibitors of CYP3A4 may lead to higher drug concentrations, resulting in a higher risk of ADRs [23].

In our study, a total of 12 patients experienced 15 adverse reactions. Some of these adverse reactions can be explained by the abovementioned theories. For example, in the FDA-approved drug label information, adverse effects of nifedipine [24] have been seen with thrombocytopenia, leukopenia, and damage to the heart. Also, elevated creatine kinase has been found in patients using nifedipine, but the relationship with nifedipine treatment is uncertain. The combination of nifedipine with voriconazole, a strong inhibitor of CYP3A4, increases the blood concentration of nifedipine and may aggravate the adverse effects. In addition, voriconazole [25] also has side effects of granulocyte deficiency, thrombocytopenia, and leukopenia. The combination of the two drugs may increase the chance and severity of these adverse reactions. Similarly, amlodipine [26] can cause leukopenia, and fluconazole [27] has leukopenia and neutropenia. The combination of amlodipine and fluconazole or voriconazole to produce ADRs could be similarly understood using the abovementioned theory.

Some adverse reactions cannot be the direct side effects of drugs but rather further injuries. For example, the possible mechanism of acute kidney injury (AKI) is caused by the combination of nifedipine and voriconazole. Due to the effective inhibition of CYP3A4 by voriconazole, voriconazole increases the blood concentration of nifedipine and excessively enhances its hypotensive effect. Severe hypotension may lead to inadequate renal perfusion, resulting in ischemic AKI [28]. In addition, there were still some adverse reaction symptoms that may be natural history or complication of the patient's primary disease, such as systemic lupus erythematosus, which may have manifestations of rhabdomyolysis [29, 30].

There are still some limitations to our study. First, our study excluded Chinese herbal medicines, which studies have shown are metabolized by cytochrome P450 and can be involved in interactions [31]. However, due to the complex composition of herbal medicines and the unspecified metabolizing enzymes of some components, the pDDIs involving herbal medicines were not evaluated in this study. Secondly, we explored the occurrence of adverse reactions based on the laboratory test results because there were no drug monitoring data or adverse reaction records in the database. However, changes in laboratory test results may be due to a variety of reasons, not all of which are caused by pDDIs. Moreover, there were many adverse reactions, such as DDI-induced tardive dyskinesia, which could not be identified by the available data.

However, our study can still provide some reference to ICU children's care. We found that CYP-mediated pDDIs were still occurring in the children admitted to ICUs. CYP-mediated interactions are usually measurable and, therefore, preventable. We recommend using clinical decision support systems such as Lexicomp to try avoiding combinations that would produce serious adverse effects. Sometimes, the combination of these drugs may be unavoidable. So, we recommend monitoring the serum drug concentrations and paying attention to clinical monitoring for possible adverse reactions. We have also identified a number of drugs that are associated with pDDIs and the occurrence of adverse reactions, such as voriconazole. The risk of pDDIs and adverse reactions may be significantly reduced if these drugs are appropriately discontinued or switched to other drugs with the same pharmacological effects.

## 5. Conclusions

We explored the occurrence of clinically significant CYP-mediated pDDIs in ICUs at a large children's hospital in China and identified adverse reactions based on laboratory test results. We recommend the use of clinical decision support systems in ICUs to improve medication safety as well as better monitoring.

## Figures and Tables

**Table 1:** Characteristics of the study population (*n* = 84).

Characteristics	*N* (%) or median (range)
Gender	
Female	43 (51.19)
Male	41 (48.81)
Age group	
<1 month	17 (20.24)
1 month–1 year	19 (22.62)
2years–5 years	12 (14.29)
6years–11 years	25 (29.76)
12 years–17 years	11 (13.10)
Main diagnosis	
Diseases of the respiratory system	20 (23.81)
Neoplasms	16 (19.05)
Certain conditions originating in the perinatal period	12 (14.29)
Diseases of the musculoskeletal system and connective tissue	8 (9.52)
Diseases of the blood and blood-forming organs involving the immune mechanism	8 (9.52)
Congenital malformation deformations and chromosomal abnormalities	5 (5.95)
Certain infectious and parasitic diseases	4 (4.76)
Diseases of the genitourinary system	4 (4.76)
Injury, poisoning, and certain other consequences of external causes	2 (2.38)
Diseases of the nervous system	2 (2.38)
Diseases of the digestive system	1 (1.19)
Diseases of the circulatory system	1 (1.19)
Factors influencing health status and contact with health services	1 (1.19)
Length of stay (days)	37.5 (6–335)
Total number of drugs prescribed	45 (23–103)

**Table 2:** Clinically significant pDDIs (*n* = 101).

pDDIs	*N* (%)	CYP isoenzyme	Reliability rating
Nifedipine + voriconazole	40 (39.60)	CYP3A4	Good
Erythromycin + fluconazole	34 (33.66)	CYP3A4	Good
Amlodipine + voriconazole	12 (11.88)	CYP3A4	Good
Erythromycin + voriconazole	7 (6.93)	CYP3A4	Good
Fluconazole + fluconazole	3 (2.97)	CYP3A4	Good
Amlodipine + fluconazole	3 (2.79)	CYP3A4	Good
Amlodipine + posaconazole	1 (0.99)	CYP3A4	Good
Domperidone + voriconazole	1 (0.99)	CYP3A4	Good

pDDIs = potential drug-drug interactions.

**Table 3:** Drugs involved in pDDIs (*n* = 202).

Drugs	*N* (%)	ATC classification
Voriconazole	60 (29.70)	J02AC03
Nifedipine	40 (19.80)	C08CA05
Erythromycin	40 (19.80)	J01FA01
Fluconazole	38 (18.81)	J02AC01
Amlodipine	17 (8.42)	C08CA01
Carbamazepine	3 (1.49)	N03AF01
Posaconazole	1 (0.50)	J02AC04
Clarithromycin	1 (0.50)	J01FA09
Domperidone	1 (0.50)	A03FA03
Simvastatin	1 (0.50)	C10AA01

pDDIs = potential drug-drug interactions; ATC = anatomical therapeutic chemical.

**Table 4:** The occurrence of ADRs (*n* = 15).

No.	Age	Gender	Length of stay (days)	Diagnosis	DDI pairings	ADRs
1	11 years	Male	40	Systemic lupus erythematosus	Amlodipine + voriconazole	Rhabdomyolysis
2	14 years	Male	29	Aplastic anemia	Nifedipine + voriconazole	Rhabdomyolysis
3 (1)	11 years	Male	40	Systemic lupus erythematosus	Nifedipine + voriconazole	Rhabdomyolysis
4	13 years	Female	38	Leukocythemia	Erythromycin + voriconazole	Rhabdomyolysis
5	10 years	Female	20	Hematuria, with minor glomerular lesions	Amlodipine + voriconazole	Leukopenia
6	3 years	Male	23	Aplastic anemia	Nifedipine + voriconazole	Leukopenia
7	7 years	Female	33	Juvenile idiopathic arthritis	Nifedipine + voriconazole	Leukopenia
8	6 years	Female	28	Pneumonia	Nifedipine + voriconazole	Leukopenia
9 (7)	7 years	Female	33	Juvenile idiopathic arthritis	Amlodipine + voriconazole	Neutropenia
10	6 years	Female	64	Pneumonia	Amlodipine + fluconazole	Neutropenia
11 (7)	7 years	Female	33	Juvenile idiopathic arthritis	Nifedipine + voriconazole	Neutropenia
12	3 years	Male	30	Hemophagocytic lymphohistiocytosis	Nifedipine + voriconazole	Acute kidney injury
13	6 years	Male	10	Muscular dystrophy	Nifedipine + voriconazole	Acute kidney injury
14	11 years	Female	47	Leukocythemia	Nifedipine + voriconazole	Myocardial injury
15	1 years	Female	22	Pneumonia	Nifedipine + voriconazole	Thrombocytopenia

The numbers in parentheses represent the same patients as the previous numbers. ADRs = adverse drug reactions; DDI pairings = drug-drug interactions pairings.

## Data Availability

The data used to support the findings of this study may be released upon request.

## Supplementary Materials

(Supplementary Materials) The diagnostic criteria for adverse reactions based on laboratory test results used in this study are found as supplementary methods. Information on CYP-mediated pDDI pairings included in this study could be found in supplementary table.
