# Supplementary material for: Clinically Significant Cytochrome P450-Mediated Drug-Drug Interactions in Children Admitted to Intensive Care Units
Source: Int J Clin Pract. 2022 Aug 23;2022:2786914. doi: 10.1155/2022/2786914 (PMC9427250; doi:10.1155/2022/2786914)
Supplement: (Supplementary Materials) — The diagnostic criteria for adverse reactions based on laboratory test results used in this study are found as supplementary methods. Information on CYP-mediated pDDI pairings included in this study could be found in supplementary table. [file 2786914.f1.zip › 2786914.f1/Supplementary methods.docx]

## Supplementary methods

The diagnostic criteria for adverse reactions based on laboratory test results used in this study.

Rhabdomyolysi [1]: Serum creatine kinase (CK) level is > 1000 U/L or at least 5x the upper limit of normal.

Neutropenia [2]: Neutrophil count < 0.5×10^9^/L.

Thrombocytopenia [3]: Platelet count < 50×10^9^/L.

Leukopenia [4]: White blood cell (WBC) count < 4×10^9^/L.

Myocardial injury [5]: Cardiac troponin (cTn) above the 99th percentile of the upper limit of normal reference.

Acute kidney injury [6]: An increase in serum creatinine (Scr) by 0.3 mg/dl within 48 hours or a 50% increase in serum creatinine from the baseline within 7 days.

Supplementary references

[1] Cabral B, Edding SN, Portocarrero JP, Lerma EV. Rhabdomyolysis. Dis Mon. 2020;66:101015. <https://doi.org/10.1016/j.disamonth.2020.101015.>

[2] Andrès E, Mourot-Cottet R. Non-chemotherapy drug-induced neutropenia-an update. Expert Opin Drug Saf. 2017;16:1235-42. <https://doi.org/10.1080/14740338.2017.1376645.>

[3] Danese E, Montagnana M, Favaloro EJ, Lippi G. Drug-induced thrombocytopenia: Mechanisms and laboratory diagnostics. Semin Thromb Hemost. 2020;46:264-74. <https://doi.org/10.1055/s-0039-1697930.>

[4] van Gennep S, Konté K, Meijer B, Heymans MW, D'Haens GR, Löwenberg M, et al. Systematic review with meta-analysis: risk factors for thiopurine-induced leukopenia in IBD. Aliment Pharmacol Ther. 2019;50:484-506. <https://doi.org/10.1111/apt.15403.>

[5] Thygesen K, Alpert JS, Jaffe AS, Chaitman BR, Bax JJ, Morrow DA, et al. Fourth Universal Definition of Myocardial Infarction (2018). J Am Coll Cardiol. 2018;72:2231-64. <https://doi.org/10.1016/j.jacc.2018.08.1038.>

[6] Levey AS, Levin A, Kellum JA. Definition and classification of kidney diseases. Am J Kidney Dis. 2013;61:686-88. <https://doi.org/10.1053/j.ajkd.2013.03.003.>
