# Supplementary material for: Clinically Significant Cytochrome P450-Mediated Drug-Drug Interactions in Children Admitted to Intensive Care Units
Source: Int J Clin Pract. 2022 Aug 23;2022:2786914. doi: 10.1155/2022/2786914 (PMC9427250; doi:10.1155/2022/2786914)
Supplement: (Supplementary Materials) — The diagnostic criteria for adverse reactions based on laboratory test results used in this study are found as supplementary methods. Information on CYP-mediated pDDI pairings included in this study could be found in supplementary table. [file 2786914.f1.zip › 2786914.f1/Supplementary table.docx]

**Supplementary table: Information on CYP-mediated DDI pairings**

| Title | Members of Drug 1 | Members of Drug 2 | CYP isoenzyme | Clinical Phenotype | Risk Rating | Reliability Rating | Severity | Patient Management | Summary |
| --- | --- | --- | --- | --- | --- | --- | --- | --- | --- |
| Nateglinide / CYP2C9 Inhibitors (Moderate) | Nateglinide | CYP2C9 Inhibitors (Moderate) Interacting Members Fluconazole, MiFEPRIStone, Nitisinone | CYP2C9 | Hypoglycemia | C: Monitor therapy | Reliability Rating Good | Severity Moderate | Monitor patients for increased nateglinide effects (eg, hypoglycemia) if combined with moderate CYP2C9 inhibitors. | CYP2C9 Inhibitors (Moderate) may increase the serum concentration of Nateglinide. |
| Perhexiline / CYP2D6 Inhibitors (Moderate) | Perhexiline | CYP2D6 Inhibitors (Moderate) Interacting Members Abiraterone Acetate, Cinacalcet, Darifenacin, Darunavir, DULoxetine, Givosiran, Lorcaserin (Withdrawn From US Market), Mirabegron, Perhexiline, Rolapitant, Terbinafine (Systemic), Thioridazine | CYP2D6 | Hypoglycemia | C: Monitor therapy | Reliability Rating Good | Severity Moderate | Monitor for increased perhexiline serum concentrations and toxicities (eg, hypoglycemia, neuropathy, liver dysfunction) when combined with moderate CYP2D6 inhibitors. Perhexiline dose reductions may be necessary. | CYP2D6 Inhibitors (Moderate) may increase the serum concentration of Perhexiline. |
| Perhexiline / CYP2D6 Inhibitors (Strong) | Perhexiline | CYP2D6 Inhibitors (Strong) Interacting Members BuPROPion, Dacomitinib, FLUoxetine, PARoxetine, QuiNIDine, Quinidine (Non-Therapeutic), Tipranavir | CYP2D6 | Hypoglycemia | C: Monitor therapy | Reliability Rating Good | Severity Moderate | Monitor for increased perhexiline serum concentrations and toxicities (eg, hypoglycemia, neuropathy, liver dysfunction) when combined with strong CYP2D6 inhibitors. Perhexiline dose reductions may be necessary. | CYP2D6 Inhibitors (Strong) may increase the serum concentration of Perhexiline. |
| Pioglitazone / CYP2C8 Inhibitors (Moderate) | Pioglitazone | CYP2C8 Inhibitors (Moderate) Interacting Members Clopidogrel, Deferasirox, Leflunomide, Selpercatinib, Teriflunomide | CYP2C8 | Hypoglycemia | C: Monitor therapy | Reliability Rating Good | Severity Moderate | Monitor patients for increased pioglitazone effects (eg, decreased blood glucose, evidence of edema or hepatotoxicity) when combined with moderate CYP2C8 inhibitors. | CYP2C8 Inhibitors (Moderate) may increase the serum concentration of Pioglitazone. |
| Pioglitazone / CYP2C8 Inhibitors (Strong) | Pioglitazone | CYP2C8 Inhibitors (Strong) Interacting Members Gemfibrozil* | CYP2C8 | Hypoglycemia | D: Consider therapy modification | Reliability Rating Good | Severity Moderate | Limit the pioglitazone dose to 15 mg daily and monitor for increased pioglitazone toxicities (eg, decreased blood glucose, evidence of edema or hepatotoxicity) when used in combination with strong CYP2C8 inhibitors. | CYP2C8 Inhibitors (Strong) may increase the serum concentration of Pioglitazone. |
| Repaglinide / CYP2C8 Inhibitors (Strong) | Repaglinide | CYP2C8 Inhibitors (Strong) Interacting Members Gemfibrozil | CYP2C8 | Hypoglycemia | X: Avoid combination | Reliability Rating Excellent | Severity Major | Do not use repaglinide with strong CYP2C8 inhibitors. This combination is listed as contraindicated in the repaglinide prescribing information. | CYP2C8 Inhibitors (Strong) may increase the serum concentration of Repaglinide. |
| Repaglinide / CYP2C8 Inhibitors (Moderate) | Repaglinide | CYP2C8 Inhibitors (Moderate) Interacting Members Deferasirox, Leflunomide, Selpercatinib, Teriflunomide Exception Clopidogrel | CYP2C8 | Hypoglycemia | C: Monitor therapy | Reliability Rating Good | Severity Moderate | Monitor blood glucose more frequently when repaglinide is combined with moderate CYP2C8 inhibitors. Repaglinide dose reductions may be required. See separate drug interaction monographs for drugs listed as ‘Exceptions’ below. | CYP2C8 Inhibitors (Moderate) may increase the serum concentration of Repaglinide. |
| Repaglinide / CYP2C8 Inhibitors (Weak) | Repaglinide | CYP2C8 Inhibitors (Weak) Interacting Members Abiraterone Acetate, Favipiravir, PAZOPanib, Tazemetostat, Tecovirimat, Trimethoprim, Tucatinib Exception Ketoconazole (Systemic) | CYP2C8 | Hypoglycemia | C: Monitor therapy | Reliability Rating Good | Severity Moderate | Monitor blood glucose more frequently when repaglinide is combined with weak CYP2C8 inhibitors. Repaglinide dose reductions may be required. See separate drug interaction monographs for drugs listed as ‘Exceptions’ below. | CYP2C8 Inhibitors (Weak) may increase the serum concentration of Repaglinide. |
| Repaglinide / CYP3A4 Inhibitors (Strong) | Repaglinide | CYP3A4 Inhibitors (Strong) Interacting Members Atazanavir, Ceritinib, Clarithromycin, Cobicistat, Darunavir, Idelalisib, Indinavir, Itraconazole, Ketoconazole (Systemic), Lonafarnib, Lopinavir, MiFEPRIStone, Nefazodone, Nelfinavir, Ombitasvir, Paritaprevir, and Ritonavir, Ombitasvir, Paritaprevir, Ritonavir, and Dasabuvir, Posaconazole, Ritonavir, Saquinavir, Telithromycin, Tucatinib, Voriconazole | CYP3A4 | Hypoglycemia | C: Monitor therapy | Reliability Rating Excellent | Severity Moderate | Monitor for increased hypoglycemic response to repaglinide when used in combination with a strong CYP3A4 inhibitor. Repaglinide dose reductions may be required. | CYP3A4 Inhibitors (Strong) may increase the serum concentration of Repaglinide. |
| SAXagliptin / CYP3A4 Inhibitors (Moderate) | SAXagliptin | CYP3A4 Inhibitors (Moderate) Interacting Members Aprepitant, Berotralstat, Conivaptan, Crizotinib, DilTIAZem, Dronedarone, Duvelisib, Erythromycin (Systemic), Fedratinib, Fluconazole, Fosamprenavir, Fosnetupitant, Grapefruit Juice, Imatinib, Isavuconazonium Sulfate, Lefamulin, Letermovir, Netupitant, Nilotinib, Ribociclib, Schisandra, Verapamil | CYP3A4 | Hypoglycemia | C: Monitor therapy | Reliability Rating Good | Severity Moderate | Monitor blood glucose concentrations and other markers of clinical response to saxagliptin closely when using saxagliptin together with drugs that are moderate inhibitors of CYP3A4. | CYP3A4 Inhibitors (Moderate) may increase the serum concentration of SAXagliptin. |
| SAXagliptin / CYP3A4 Inhibitors (Strong) | SAXagliptin | CYP3A4 Inhibitors (Strong) Interacting Members Atazanavir, Ceritinib, Clarithromycin, Cobicistat, Darunavir, Idelalisib, Indinavir, Itraconazole, Ketoconazole (Systemic), Lonafarnib, Lopinavir, MiFEPRIStone, Nefazodone, Nelfinavir, Ombitasvir, Paritaprevir, and Ritonavir, Ombitasvir, Paritaprevir, Ritonavir, and Dasabuvir, Posaconazole, Ritonavir, Saquinavir, Telithromycin, Tucatinib, Voriconazole | CYP3A4 | Hypoglycemia | D: Consider therapy modification | Reliability Rating Good | Severity Moderate | Limit the saxagliptin dose to 2.5 mg daily when combined with strong CYP3A4 inhibitors. When using the saxagliptin combination products saxagliptin/dapagliflozin or saxagliptin/dapagliflozin/metformin, avoid use with strong CYP3A4 inhibitors. | CYP3A4 Inhibitors (Strong) may increase the serum concentration of SAXagliptin. |
| Sulfonylureas / CYP2C9 Inhibitors (Moderate) | ChlorproPAMIDE*, Gliclazide*, Glimepiride, GlipiZIDE*, GlyBURIDE*, TOLAZamide*, TOLBUTamide* | CYP2C9 Inhibitors (Moderate) Interacting Members Fluconazole, MiFEPRIStone, Nitisinone | CYP2C9 | Hypoglycemia | C: Monitor therapy | Reliability Rating Good | Severity Moderate | Monitor for increased sulfonylurea effects and toxicities (ie, hypoglycemia) when combined with moderate CYP2C9 inhibitors. | CYP2C9 Inhibitors (Moderate) may increase the serum concentration of Sulfonylureas. |
| TOLBUTamide / CYP2C9 Inhibitors (Weak) | TOLBUTamide | CYP2C9 Inhibitors (Weak) Interacting Members Amiodarone, Amodiaquine, Benzbromarone, Berotralstat, Capecitabine, Ceritinib, CycloSPORINE (Systemic), Doxifluridine, Floxuridine, Fluorouracil (Systemic), FluvoxaMINE, MetroNIDAZOLE (Systemic), Milk Thistle, Quercetin, Resveratrol, Rucaparib, Sulfinpyrazone, Tamoxifen, Tegafur, Zafirlukast Exceptions Chloramphenicol (Systemic), Fenofibrate and Derivatives, Voriconazole | CYP2C9 | Hypoglycemia | C: Monitor therapy | Reliability Rating Good | Severity Moderate | Monitor for increased tolbutamide effects and toxicities (ie, hypoglycemia) when combined with weak CYP2C9 inhibitors. See separate drug interaction monographs for drugs listed as ‘Exceptions’ below. | CYP2C9 Inhibitors (Weak) may increase the serum concentration of TOLBUTamide. |
| Nateglinide / CYP3A4 Inducers (Strong) | Nateglinide | CYP3A4 Inducers (Strong) Interacting Members Apalutamide, CarBAMazepine, Enzalutamide, Fosphenytoin, Lumacaftor and Ivacaftor, Mitotane, PHENobarbital, Phenytoin, Primidone, RifAMPin | CYP3A4 | Hyperglycemia | C: Monitor therapy | Reliability Rating Good | Severity Moderate | Monitor for decreased nateglinide efficacy (ie, hyperglycemia) if combined with strong CYP3A4 inducers. Nateglinide dose increases may be needed. | CYP3A4 Inducers (Strong) may decrease the serum concentration of Nateglinide. |
| Repaglinide / CYP3A4 Inducers (Strong) | Repaglinide | CYP3A4 Inducers (Strong) Interacting Members Apalutamide, CarBAMazepine, Enzalutamide, Fosphenytoin, Lumacaftor and Ivacaftor, Mitotane, PHENobarbital, Phenytoin, Primidone Exception RifAMPin | CYP3A4 | Hyperglycemia | C: Monitor therapy | Reliability Rating Good | Severity Moderate | Monitor for decreased therapeutic effects of repaglinide (eg, hyperglycemia) when combined with strong CYP3A4 inducers. Repaglinide dose increases may be required. See separate drug interaction monographs for drugs listed as ‘Exceptions’ below. | CYP3A4 Inducers (Strong) may decrease the serum concentration of Repaglinide. |
| Sulfonylureas / CYP2C9 Inducers (Moderate) | ChlorproPAMIDE*, Gliclazide*, Glimepiride, GlipiZIDE*, GlyBURIDE*, TOLAZamide*, TOLBUTamide* | CYP2C9 Inducers (Moderate) Interacting Members Enzalutamide, RifAMPin | CYP2C9 | Hyperglycemia | C: Monitor therapy | Reliability Rating Good | Severity Moderate | Monitor for reduced sulfonylurea effectiveness (eg, hyperglycemia) when combined with moderate CYP2C9 inducers. | CYP2C9 Inducers (Moderate) may decrease the serum concentration of Sulfonylureas. |
| Alfuzosin / CYP3A4 Inhibitors (Moderate) | Alfuzosin | CYP3A4 Inhibitors (Moderate) Interacting Members Aprepitant, Berotralstat, Conivaptan, Crizotinib, DilTIAZem, Dronedarone, Duvelisib, Erythromycin (Systemic), Fedratinib, Fluconazole, Fosamprenavir, Fosnetupitant, Grapefruit Juice, Imatinib, Isavuconazonium Sulfate, Lefamulin, Letermovir, Netupitant, Nilotinib, Ribociclib, Schisandra, Verapamil | CYP3A4 | Hypotension | C: Monitor therapy | Reliability Rating Good | Severity Moderate | Monitor for increased alfuzosin effects and toxicities (eg, hypotension) when combined with moderate CYP3A4 inhibitors. | CYP3A4 Inhibitors (Moderate) may increase the serum concentration of Alfuzosin. |
| Alfuzosin / CYP3A4 Inhibitors (Strong) | Alfuzosin | CYP3A4 Inhibitors (Strong) Interacting Members Atazanavir, Ceritinib, Clarithromycin, Cobicistat, Darunavir, Idelalisib, Indinavir, Itraconazole, Ketoconazole (Systemic), Lonafarnib, Lopinavir, MiFEPRIStone, Nefazodone, Nelfinavir, Ombitasvir, Paritaprevir, and Ritonavir, Ombitasvir, Paritaprevir, Ritonavir, and Dasabuvir, Posaconazole, Ritonavir, Saquinavir, Telithromycin, Tucatinib, Voriconazole | CYP3A4 | Hypotension | X: Avoid combination | Reliability Rating Good | Severity Major | Do not use alfuzosin with a strong CYP3A4 inhibitor. This combination is listed as a contraindication in the alfuzosin prescribing information. | CYP3A4 Inhibitors (Strong) may increase the serum concentration of Alfuzosin. |
| AmLODIPine / CYP3A4 Inhibitors (Moderate) | AmLODIPine | CYP3A4 Inhibitors (Moderate) Interacting Members Aprepitant, Berotralstat, Conivaptan, Crizotinib, DilTIAZem, Dronedarone, Duvelisib, Erythromycin (Systemic), Fedratinib, Fluconazole, Fosamprenavir, Fosnetupitant, Grapefruit Juice, Imatinib, Isavuconazonium Sulfate, Lefamulin, Letermovir, Netupitant, Nilotinib, Ribociclib, Schisandra, Verapamil | CYP3A4 | Hypotension | C: Monitor therapy | Reliability Rating Good | Severity Minor | Monitor for increased amlodipine effects and toxicities (eg, hypotension, edema) when combined with a moderate CYP3A4 inhibitor. | CYP3A4 Inhibitors (Moderate) may increase the serum concentration of AmLODIPine. |
| AmLODIPine / CYP3A4 Inhibitors (Strong) | AmLODIPine | CYP3A4 Inhibitors (Strong) Interacting Members Atazanavir, Ceritinib, Clarithromycin, Cobicistat, Darunavir, Idelalisib, Indinavir, Itraconazole, Ketoconazole (Systemic), Lonafarnib, Lopinavir, MiFEPRIStone, Nefazodone, Nelfinavir, Ombitasvir, Paritaprevir, and Ritonavir, Ombitasvir, Paritaprevir, Ritonavir, and Dasabuvir, Posaconazole, Ritonavir, Saquinavir, Telithromycin, Tucatinib, Voriconazole | CYP3A4 | Hypotension | C: Monitor therapy | Reliability Rating Good | Severity Moderate | Monitor for increased amlodipine effects and toxicities (eg, hypotension, edema) when combined with a strong CYP3A4 inhibitor and consider a 50% amlodipine dose reduction. | CYP3A4 Inhibitors (Strong) may increase the serum concentration of AmLODIPine. |
| Avanafil / CYP3A4 Inhibitors (Moderate) | Avanafil | CYP3A4 Inhibitors (Moderate) Interacting Members Aprepitant, Berotralstat, Conivaptan, Crizotinib, DilTIAZem, Dronedarone, Duvelisib, Erythromycin (Systemic), Fedratinib, Fluconazole, Fosamprenavir, Fosnetupitant, Grapefruit Juice, Imatinib, Isavuconazonium Sulfate, Lefamulin, Letermovir, Netupitant, Nilotinib, Ribociclib, Schisandra, Verapamil | CYP3A4 | Hypotension | D: Consider therapy modification | Reliability Rating Good | Severity Moderate | The maximum avanafil dose is 50 mg per 24-hour period when used together with a moderate CYP3A4 inhibitor. Patients receiving such a combination should also be monitored more closely for evidence of adverse effects (eg, hypotension, syncope, priapism). | CYP3A4 Inhibitors (Moderate) may increase the serum concentration of Avanafil. |
| Avanafil / CYP3A4 Inhibitors (Strong) | Avanafil | CYP3A4 Inhibitors (Strong) Interacting Members Atazanavir, Ceritinib, Clarithromycin, Cobicistat, Darunavir, Idelalisib, Indinavir, Itraconazole, Ketoconazole (Systemic), Lonafarnib, Lopinavir, MiFEPRIStone, Nefazodone, Nelfinavir, Ombitasvir, Paritaprevir, and Ritonavir, Ombitasvir, Paritaprevir, Ritonavir, and Dasabuvir, Posaconazole, Ritonavir, Saquinavir, Telithromycin, Tucatinib, Voriconazole | CYP3A4 | Hypotension | X: Avoid combination | Reliability Rating Good | Severity Major | Avanafil should not be used in patients who are receiving a strong CYP3A4 inhibitor. | CYP3A4 Inhibitors (Strong) may increase the serum concentration of Avanafil. |
| Benidipine / CYP3A4 Inhibitors (Moderate) | Benidipine | CYP3A4 Inhibitors (Moderate) Interacting Members Aprepitant, Berotralstat, Conivaptan, Crizotinib, DilTIAZem, Dronedarone, Duvelisib, Erythromycin (Systemic), Fedratinib, Fluconazole, Fosamprenavir, Fosnetupitant, Grapefruit Juice, Imatinib, Isavuconazonium Sulfate, Lefamulin, Letermovir, Netupitant, Nilotinib, Ribociclib, Schisandra, Verapamil | CYP3A4 | Hypotension | C: Monitor therapy | Reliability Rating Good | Severity Minor | Monitor for increased benidipine effects and toxicities (eg, hypotension, edema) if combined with moderate CYP3A4 inhibitors. | CYP3A4 Inhibitors (Moderate) may increase the serum concentration of Benidipine. |
| Benidipine / CYP3A4 Inhibitors (Strong) | Benidipine | CYP3A4 Inhibitors (Strong) Interacting Members Atazanavir, Ceritinib, Clarithromycin, Cobicistat, Darunavir, Idelalisib, Indinavir, Itraconazole, Ketoconazole (Systemic), Lonafarnib, Lopinavir, MiFEPRIStone, Nefazodone, Nelfinavir, Ombitasvir, Paritaprevir, and Ritonavir, Ombitasvir, Paritaprevir, Ritonavir, and Dasabuvir, Posaconazole, Ritonavir, Saquinavir, Telithromycin, Tucatinib, Voriconazole | CYP3A4 | Hypotension | C: Monitor therapy | Reliability Rating Good | Severity Moderate | Monitor for increased benidipine effects and toxicities (eg, hypotension, edema) if combined with strong CYP3A4 inhibitors. | CYP3A4 Inhibitors (Strong) may increase the serum concentration of Benidipine. |
| Carvedilol / CYP2C9 Inhibitors (Moderate) | Carvedilol | CYP2C9 Inhibitors (Moderate) Interacting Members Fluconazole, MiFEPRIStone, 尼替西农 | CYP2C9 | Hypotension | C: Monitor therapy | Reliability Rating Good | Severity Moderate | Monitor patients closely for signs and symptoms of excessive response to carvedilol (eg, hypotension, bradycardia, orthostasis) when adding a moderate CYP2C9 inhibitor to existing carvedilol therapy or vice versa. | CYP2C9 Inhibitors (Moderate) may increase the serum concentration of Carvedilol. Specifically, concentrations of the S-carvedilol enantiomer may be increased. |
| Carvedilol / CYP2D6 Inhibitors (Strong) | Carvedilol | CYP2D6 Inhibitors (Strong) Interacting Members BuPROPion, Dacomitinib, FLUoxetine, PARoxetine, QuiNIDine, Quinidine (Non-Therapeutic), Tipranavir | CYP2D6 | Hypotension | C: Monitor therapy | Reliability Rating Good | Severity Moderate | Monitor patients closely for signs and symptoms of excessive response to carvedilol (eg, hypotension, bradycardia, orthostasis) when used together with strong inhibitors of CYP2D6. | CYP2D6 Inhibitors (Strong) may increase the serum concentration of Carvedilol. |
| DilTIAZem / CYP3A4 Inhibitors (Moderate) | DilTIAZem | CYP3A4 Inhibitors (Moderate) Interacting Members Aprepitant, Berotralstat, Conivaptan, Crizotinib, DilTIAZem, Dronedarone, Duvelisib, Fedratinib, Fluconazole, Fosamprenavir, Fosnetupitant, Grapefruit Juice, Imatinib, Isavuconazonium Sulfate, Lefamulin, Letermovir, Netupitant, Nilotinib, Ribociclib, Schisandra, Verapamil Exception Erythromycin (Systemic) | CYP3A4 | Hypotension | C: Monitor therapy | Reliability Rating Good | Severity Minor | Monitor for increased diltiazem effects and toxicities (eg, hypotension, bradycardia) when combined with moderate CYP3A4 inhibitors. See separate drug interaction monographs for drugs listed as ‘Exceptions’ below. | CYP3A4 Inhibitors (Moderate) may increase the serum concentration of DilTIAZem. |
| Felodipine / CYP3A4 Inhibitors (Moderate) | Felodipine | CYP3A4 Inhibitors (Moderate) Interacting Members Aprepitant, Berotralstat, Conivaptan, Crizotinib, DilTIAZem, Dronedarone, Duvelisib, Erythromycin (Systemic), Fedratinib, Fluconazole, Fosamprenavir, Fosnetupitant, Imatinib, Isavuconazonium Sulfate, Lefamulin, Letermovir, Netupitant, Nilotinib, Ribociclib, Schisandra, Verapamil Exception Grapefruit Juice | CYP3A4 | Hypotension | C: Monitor therapy | Reliability Rating Good | Severity Moderate | Monitor for increased felodipine effects and toxicities (eg, hypotension, edema) if combined with moderate CYP3A4 inhibitors. See separate drug interaction monographs for drugs listed as ‘Exceptions’ below. | CYP3A4 Inhibitors (Moderate) may increase the serum concentration of Felodipine. |
| Felodipine / CYP3A4 Inhibitors (Strong) | Felodipine | CYP3A4 Inhibitors (Strong) Interacting Members Atazanavir, Ceritinib, Clarithromycin, Cobicistat, Darunavir, Idelalisib, Indinavir, Itraconazole, Ketoconazole (Systemic), Lonafarnib, Lopinavir, MiFEPRIStone, Nefazodone, Nelfinavir, Ombitasvir, Paritaprevir, and Ritonavir, Ombitasvir, Paritaprevir, Ritonavir, and Dasabuvir, Posaconazole, Ritonavir, Saquinavir, Telithromycin, Tucatinib, Voriconazole | CYP3A4 | Hypotension | D: Consider therapy modification | Reliability Rating Good | Severity Moderate | Consider using lower felodipine doses when combined with strong CYP3A4 inhibitors. Monitor patients for increased felodipine effects and toxicities (eg, hypotension, edema) when these agents are combined. | CYP3A4 Inhibitors (Strong) may increase the serum concentration of Felodipine. |
| GuanFACINE / CYP3A4 Inhibitors (Moderate) | GuanFACINE | CYP3A4 Inhibitors (Moderate) Interacting Members Aprepitant, Berotralstat, Conivaptan, Crizotinib, DilTIAZem, Dronedarone, Duvelisib, Erythromycin (Systemic), Fedratinib, Fluconazole, Fosamprenavir, Fosnetupitant, Grapefruit Juice, Imatinib, Isavuconazonium Sulfate, Lefamulin, Letermovir, Netupitant, Nilotinib, Ribociclib, Schisandra, Verapamil | CYP3A4 | Hypotension | D: Consider therapy modification | Reliability Rating Fair: Reported in the prescribing information | Severity Moderate | Reduce the extended-release guanfacine dose by 50% when combined with a moderate CYP3A4 inhibitor. When discontinuing moderate CYP3A4 inhibitor treatment, increase the extended-release guanfacine dose to the recommended dose range. No specific dose adjustments are provided for use with immediate release guanfacine, but patients receiving any formulation of guanfacine with a moderate CYP3A4 inhibitor should be monitored closely for evidence of excessive guanfacine response (eg, hypotension, bradycardia, CNS depression). | CYP3A4 Inhibitors (Moderate) may increase the serum concentration of GuanFACINE. |
| GuanFACINE / CYP3A4 Inhibitors (Strong) | GuanFACINE | CYP3A4 Inhibitors (Strong) Interacting Members Atazanavir, Ceritinib, Clarithromycin, Cobicistat, Darunavir, Idelalisib, Indinavir, Itraconazole, Ketoconazole (Systemic), Lonafarnib, Lopinavir, MiFEPRIStone, Nefazodone, Nelfinavir, Ombitasvir, Paritaprevir, and Ritonavir, Ombitasvir, Paritaprevir, Ritonavir, and Dasabuvir, Posaconazole, Ritonavir, Saquinavir, Telithromycin, Tucatinib, Voriconazole | CYP3A4 | Hypotension | D: Consider therapy modification | Reliability Rating Good | Severity Moderate | Reduce the extended-release guanfacine dose by 50% when combined with a strong CYP3A4 inhibitor. When discontinuing strong CYP3A4 inhibitor treatment, increase the extended-release guanfacine dose to the recommended dose range. No specific dose adjustments are provided for use with immediate release guanfacine, but patients receiving any formulation of guanfacine with a strong CYP3A4 inhibitor should be monitored closely for evidence of excessive guanfacine response (eg, hypotension, bradycardia, CNS depression). | CYP3A4 Inhibitors (Strong) may increase the serum concentration of GuanFACINE. |
| Isradipine / CYP3A4 Inhibitors (Moderate) | Isradipine | CYP3A4 Inhibitors (Moderate) Interacting Members Aprepitant, Berotralstat, Conivaptan, Crizotinib, DilTIAZem, Dronedarone, Duvelisib, Erythromycin (Systemic), Fedratinib, Fluconazole, Fosamprenavir, Fosnetupitant, Grapefruit Juice, Imatinib, Isavuconazonium Sulfate, Lefamulin, Letermovir, Netupitant, Nilotinib, Ribociclib, Schisandra, Verapamil | CYP3A4 | Hypotension | C: Monitor therapy | Reliability Rating Good | Severity Minor | Monitor for increased isradipine effects and toxicities (eg, hypotension, edema) if combined with moderate CYP3A4 inhibitors. | CYP3A4 Inhibitors (Moderate) may increase the serum concentration of Isradipine. |
| Isradipine / CYP3A4 Inhibitors (Strong) | Isradipine | CYP3A4 Inhibitors (Strong) Interacting Members Atazanavir, Ceritinib, Clarithromycin, Cobicistat, Darunavir, Idelalisib, Indinavir, Itraconazole, Ketoconazole (Systemic), Lonafarnib, Lopinavir, MiFEPRIStone, Nefazodone, Nelfinavir, Ombitasvir, Paritaprevir, and Ritonavir, Ombitasvir, Paritaprevir, Ritonavir, and Dasabuvir, Posaconazole, Ritonavir, Saquinavir, Telithromycin, Tucatinib, Voriconazole | CYP3A4 | Hypotension | C: Monitor therapy | Reliability Rating Good | Severity Moderate | Monitor for increased isradipine effects and toxicities (eg, hypotension, edema) if combined with strong CYP3A4 inhibitors. | CYP3A4 Inhibitors (Strong) may increase the serum concentration of Isradipine. |
| Levamlodipine / CYP3A4 Inhibitors (Moderate) | Levamlodipine | CYP3A4 Inhibitors (Moderate) Interacting Members Aprepitant, Berotralstat, Conivaptan, Crizotinib, DilTIAZem, Dronedarone, Duvelisib, Erythromycin (Systemic), Fedratinib, Fluconazole, Fosamprenavir, Fosnetupitant, Grapefruit Juice, Imatinib, Isavuconazonium Sulfate, Lefamulin, Letermovir, Netupitant, Nilotinib, Ribociclib, Schisandra, Verapamil | CYP3A4 | Hypotension | C: Monitor therapy | Reliability Rating Good | Severity Minor | Monitor for increased levamlodipine effects and toxicities (eg, hypotension, edema) when combined with a moderate CYP3A4 inhibitor. | CYP3A4 Inhibitors (Moderate) may increase the serum concentration of Levamlodipine. |
| Levamlodipine / CYP3A4 Inhibitors (Strong) | Levamlodipine | CYP3A4 Inhibitors (Strong) Interacting Members Atazanavir, Ceritinib, Clarithromycin, Cobicistat, Darunavir, Idelalisib, Indinavir, Itraconazole, Ketoconazole (Systemic), Lonafarnib, Lopinavir, MiFEPRIStone, Nefazodone, Nelfinavir, Ombitasvir, Paritaprevir, and Ritonavir, Ombitasvir, Paritaprevir, Ritonavir, and Dasabuvir, Posaconazole, Ritonavir, Saquinavir, Telithromycin, Tucatinib, Voriconazole | CYP3A4 | Hypotension | C: Monitor therapy | Reliability Rating Good | Severity Moderate | Monitor for increased levamlodipine effects and toxicities (eg, hypotension, edema) when combined with a strong CYP3A4 inhibitor and consider a 50% levamlodipine dose reduction. | CYP3A4 Inhibitors (Strong) may increase the serum concentration of Levamlodipine. |
| Lofexidine / CYP2D6 Inhibitors (Strong) | Lofexidine | CYP2D6 Inhibitors (Strong) Interacting Members BuPROPion, Dacomitinib, FLUoxetine, PARoxetine, Quinidine (Non-Therapeutic), Tipranavir Exception QuiNIDine | CYP2D6 | Hypotension | C: Monitor therapy | Reliability Rating Good | Severity Moderate | Clinical monitoring for signs and symptoms of lofexidine toxicity (eg, orthostatic hypotension, bradycardia) is warranted when coadministering lofexidine and strong CYP2D6 inhibitors. See separate drug interaction monographs for drugs listed as ‘Exceptions’ below. | CYP2D6 Inhibitors (Strong) may increase the serum concentration of Lofexidine. |
| Metoprolol / CYP2D6 Inhibitors (Moderate) | Metoprolol | CYP2D6 Inhibitors (Moderate) Interacting Members Abiraterone Acetate, Cinacalcet, Darifenacin, Darunavir, DULoxetine, Givosiran, Lorcaserin (Withdrawn From US Market), Mirabegron, Perhexiline, Rolapitant, Terbinafine (Systemic), Thioridazine | CYP2D6 | Hypotension | C: Monitor therapy | Reliability Rating Excellent | Severity Moderate | Monitor closely for evidence of excessive response to metoprolol (including, but not necessarily limited to, parameters such as heart rate, PR interval, blood pressure). Metoprolol dose reductions may be necessary. | CYP2D6 Inhibitors (Moderate) may increase the serum concentration of Metoprolol. |
| Metoprolol / CYP2D6 Inhibitors (Strong) | Metoprolol | CYP2D6 Inhibitors (Strong) Interacting Members BuPROPion, Dacomitinib, FLUoxetine, PARoxetine, QuiNIDine, Quinidine (Non-Therapeutic), Tipranavir | CYP2D6 | Hypotension | C: Monitor therapy | Reliability Rating Excellent | Severity Moderate | Monitor closely for evidence of excessive response to metoprolol (including, but not necessarily limited to, parameters such as heart rate, PR interval, blood pressure). Metoprolol dose reductions may be necessary. | CYP2D6 Inhibitors (Strong) may increase the serum concentration of Metoprolol. |
| Nebivolol / CYP2D6 Inhibitors (Strong) | Nebivolol | CYP2D6 Inhibitors (Strong) Interacting Members BuPROPion, Dacomitinib, FLUoxetine, PARoxetine, QuiNIDine, Quinidine (Non-Therapeutic), Tipranavir | CYP2D6 | Hypotension | C: Monitor therapy | Reliability Rating Excellent | Severity Moderate | Monitor for increased nebivolol effects and toxicities (eg, hypotension, bradycardia) when combined with strong CYP2D6 inhibitors. Adjust nebivolol doses as needed. | CYP2D6 Inhibitors (Strong) may increase the serum concentration of Nebivolol. |
| NIFEdipine / CYP3A4 Inhibitors (Moderate) | NIFEdipine | CYP3A4 Inhibitors (Moderate) Interacting Members Aprepitant, Berotralstat, Conivaptan, Crizotinib, DilTIAZem, Dronedarone, Duvelisib, Erythromycin (Systemic), Fedratinib, Fluconazole, Fosamprenavir, Fosnetupitant, Imatinib, Isavuconazonium Sulfate, Lefamulin, Letermovir, Netupitant, Nilotinib, Ribociclib, Schisandra, Verapamil Exception Grapefruit Juice | CYP3A4 | Hypotension | C: Monitor therapy | Reliability Rating Good | Severity Moderate | Monitor for increased nifedipine effects and toxicities (eg, hypotension, edema) if combined with moderate CYP3A4 inhibitors. Lower nifedipine doses may be required. See separate drug interaction monographs for drugs listed as ‘Exceptions’ below. | CYP3A4 Inhibitors (Moderate) may increase the serum concentration of NIFEdipine. |
| NIFEdipine / CYP3A4 Inhibitors (Strong) | NIFEdipine | CYP3A4 Inhibitors (Strong) Interacting Members Atazanavir, Ceritinib, Clarithromycin, Cobicistat, Darunavir, Idelalisib, Indinavir, Itraconazole, Ketoconazole (Systemic), Lonafarnib, Lopinavir, MiFEPRIStone, Nefazodone, Nelfinavir, Ombitasvir, Paritaprevir, and Ritonavir, Ombitasvir, Paritaprevir, Ritonavir, and Dasabuvir, Posaconazole, Ritonavir, Saquinavir, Telithromycin, Tucatinib, Voriconazole | CYP3A4 | Hypotension | D: Consider therapy modification | Reliability Rating Good | Severity Moderate | Consider alternatives to this combination when possible. If combined, initiate nifedipine at the lowest dose available in patients taking strong CYP3A4 inhibitors and monitor patients closely for increased nifedipine effects and toxicities (eg, hypotension, edema). | CYP3A4 Inhibitors (Strong) may increase the serum concentration of NIFEdipine. |
| Nitrendipine / CYP3A4 Inhibitors (Moderate) | Nitrendipine | CYP3A4 Inhibitors (Moderate) Interacting Members Aprepitant, Berotralstat, Conivaptan, Crizotinib, DilTIAZem, Dronedarone, Duvelisib, Erythromycin (Systemic), Fedratinib, Fluconazole, Fosamprenavir, Fosnetupitant, Grapefruit Juice, Imatinib, Isavuconazonium Sulfate, Lefamulin, Letermovir, Netupitant, Nilotinib, Ribociclib, Schisandra, Verapamil | CYP3A4 | Hypotension | C: Monitor therapy | Reliability Rating Good | Severity Moderate | Monitor for increased nitrendipine effects and toxicities (eg, hypotension, edema) when combined with moderate CYP3A4 inhibitors. | CYP3A4 Inhibitors (Moderate) may increase the serum concentration of Nitrendipine. |
| Nitrendipine / CYP3A4 Inhibitors (Strong) | Nitrendipine | CYP3A4 Inhibitors (Strong) Interacting Members Atazanavir, Ceritinib, Clarithromycin, Cobicistat, Darunavir, Idelalisib, Indinavir, Itraconazole, Ketoconazole (Systemic), Lonafarnib, Lopinavir, MiFEPRIStone, Nefazodone, Nelfinavir, Ombitasvir, Paritaprevir, and Ritonavir, Ombitasvir, Paritaprevir, Ritonavir, and Dasabuvir, Posaconazole, Ritonavir, Saquinavir, Telithromycin, Tucatinib, Voriconazole | CYP3A4 | Hypotension | C: Monitor therapy | Reliability Rating Good | Severity Moderate | Monitor for increased nitrendipine effects and toxicities (eg, hypotension, edema) when combined with strong CYP3A4 inhibitors. | CYP3A4 Inhibitors (Strong) may increase the serum concentration of Nitrendipine. |
| Propranolol / CYP2D6 Inhibitors (Strong) | Propranolol | CYP2D6 Inhibitors (Strong) Interacting Members BuPROPion, Dacomitinib, FLUoxetine, PARoxetine, QuiNIDine, Quinidine (Non-Therapeutic), Tipranavir | CYP2D6 | Hypotension | C: Monitor therapy | Reliability Rating Good | Severity Moderate | Monitor for increased propranolol effects and toxicities (eg, bradycardia, hypotension) when combined with strong CYP2D6 inhibitors. | CYP2D6 Inhibitors (Strong) may increase the serum concentration of Propranolol. |
| Riociguat / Inhibitors of CYP3A4 (Strong) and P-glycoprotein | Riociguat | Inhibitors of CYP3A4 (Strong) and P-glycoprotein Interacting Members Itraconazole, Ketoconazole (Systemic), Ombitasvir, Paritaprevir, and Ritonavir, Ritonavir, Tucatinib Exceptions Clarithromycin, Cobicistat | CYP3A4 | Hypotension | D: Consider therapy modification | Reliability Rating Good | Severity Moderate | Consider a riociguat starting dose of 0.5 mg 3 times a day when initiating riociguat in patients receiving strong CYP3A4 and P-gp inhibitors. Monitor for signs and symptoms of hypotension on initiation and on treatment with strong CYP3A4 and P-gp inhibitors. A dose reduction should be considered in patients who may not tolerate the hypotensive effect of riociguat. Exceptions to this monograph are discussed in separate drug interaction monographs. | Inhibitors of CYP3A4 (Strong) and P-glycoprotein may increase the serum concentration of Riociguat. |
| Tamsulosin / CYP2D6 Inhibitors (Strong) | Tamsulosin | CYP2D6 Inhibitors (Strong) Interacting Members BuPROPion, Dacomitinib, FLUoxetine, PARoxetine, QuiNIDine, Quinidine (Non-Therapeutic), Tipranavir | CYP2D6 | Hypotension | C: Monitor therapy | Reliability Rating Good | Severity Moderate | Monitor for increased tamsulosin effects (eg, hypotension, orthostasis) if tamsulosin is combined with a strong CYP2D6 inhibitor. Increases in tamsulosin exposure may be greater if a CYP2D6 inhibitor and a CYP3A4 inhibitor are combined with tamsulosin. | CYP2D6 Inhibitors (Strong) may increase the serum concentration of Tamsulosin. |
| Tamsulosin / CYP3A4 Inhibitors (Strong) | Tamsulosin | CYP3A4 Inhibitors (Strong) Interacting Members Atazanavir, Ceritinib, Clarithromycin, Cobicistat, Darunavir, Idelalisib, Indinavir, Itraconazole, Ketoconazole (Systemic), Lonafarnib, Lopinavir, MiFEPRIStone, Nefazodone, Nelfinavir, Ombitasvir, Paritaprevir, and Ritonavir, Ombitasvir, Paritaprevir, Ritonavir, and Dasabuvir, Posaconazole, Ritonavir, Saquinavir, Telithromycin, Tucatinib, Voriconazole | CYP3A4 | Hypotension | X: Avoid combination | Reliability Rating Good | Severity Moderate | Avoid concomitant use of tamsulosin with strong CYP3A4 inhibitors. The US manufacturer of at least one strong CYP3A4 inhibitor (boceprevir) lists its combination with tamsulosin as contraindicated. Increases in tamsulosin exposure may be greater if a CYP2D6 inhibitor and a CYP3A4 inhibitor are combined with tamsulosin. | CYP3A4 Inhibitors (Strong) may increase the serum concentration of Tamsulosin. |
| Timolol (Ophthalmic) / CYP2D6 Inhibitors (Strong) | Timolol (Ophthalmic) | CYP2D6 Inhibitors (Strong) Interacting Members BuPROPion, Dacomitinib, FLUoxetine, PARoxetine, QuiNIDine, Quinidine (Non-Therapeutic), Tipranavir | CYP2D6 | Hypotension | C: Monitor therapy | Reliability Rating Good | Severity Moderate | Monitor closely for evidence of systemic beta-blocker effects, including but not limited to orthostatic hypotension, bradycardia, and exercise intolerance. | CYP2D6 Inhibitors (Strong) may increase the serum concentration of Timolol (Ophthalmic). |
| Timolol (Systemic) / CYP2D6 Inhibitors (Strong) | Timolol (Systemic) | CYP2D6 Inhibitors (Strong) Interacting Members BuPROPion, Dacomitinib, FLUoxetine, PARoxetine, QuiNIDine, Quinidine (Non-Therapeutic), Tipranavir | CYP2D6 | Hypotension | C: Monitor therapy | Reliability Rating Good | Severity Moderate | Monitor for increased timolol effects and toxicities (eg, bradycardia, hypotension) when combined with strong CYP2D6 inhibitors. | CYP2D6 Inhibitors (Strong) may increase the serum concentration of Timolol (Systemic). |
| TiZANidine / CYP1A2 Inhibitors (Moderate) | TiZANidine | CYP1A2 Inhibitors (Moderate) Interacting Members Capmatinib, Deferasirox, Enoxacin, Givosiran, Methoxsalen (Systemic), Mexiletine, Rucaparib, Stiripentol, Thiabendazole, Vemurafenib Exception Ciprofloxacin (Systemic) | CYP1A2 | Hypotension | D: Consider therapy modification | Reliability Rating Good | Severity Moderate | Avoid the use of tizanidine with moderate CYP1A2 inhibitors when possible. If combined use cannot be avoided, initiate tizanidine at an adult dose of 2 mg and increase in 2 to 4 mg increments based on patient response. Monitor for increased effects of tizanidine, including adverse reactions (eg, hypotension, bradycardia, drowsiness). Drugs listed as exceptions are discussed in separate interaction monographs. | CYP1A2 Inhibitors (Moderate) may increase the serum concentration of TiZANidine. |
| TiZANidine / CYP1A2 Inhibitors (Strong) | TiZANidine | CYP1A2 Inhibitors (Strong) Interacting Members FluvoxaMINE*, Viloxazine | CYP1A2 | Hypotension | X: Avoid combination | Reliability Rating Good | Severity Major | Avoid concomitant use of tizanidine and strong CYP1A2 inhibitors. US prescribing information states that combined use is contraindicated. | CYP1A2 Inhibitors (Strong) may increase the serum concentration of TiZANidine. |
| TiZANidine / CYP1A2 Inhibitors (Weak) | TiZANidine | CYP1A2 Inhibitors (Weak) Interacting Members Acyclovir (Systemic), Caffeine, Cannabidiol, Cimetidine, Cola-Containing Drinks, Dipyrone, Disulfiram, Elagolix, Estradiol, and Norethindrone, Estradiol (Systemic), Estradiol (Topical), Estrogens (Conjugated A/Synthetic), Estrogens (Conjugated/Equine, Systemic), Estrogens (Conjugated/Equine, Topical), Ethinyl Estradiol, Glecaprevir and Pibrentasvir, Interferon Alfa-2b, Kola Nut, Mestranol, Obeticholic Acid, Osilodrostat, Pefloxacin, Peginterferon Alfa-2a, Peginterferon Alfa-2b, Pipemidic Acid, Propafenone, Propranolol, Simeprevir, Ticlopidine, ValACYclovir, Verapamil, Zileuton | CYP1A2 | Hypotension | D: Consider therapy modification | Reliability Rating Good | Severity Major | Avoid the use of tizanidine with weak CYP1A2 inhibitors when possible. If combined use cannot be avoided, initiate tizanidine at an adult dose of 2 mg and increase in 2 to 4 mg increments based on patient response. Monitor for increased effects of tizanidine, including adverse reactions (eg, hypotension, bradycardia, drowsiness). | CYP1A2 Inhibitors (Weak) may increase the serum concentration of TiZANidine. |
| Treprostinil / CYP2C8 Inhibitors (Strong) | Treprostinil | CYP2C8 Inhibitors (Strong) Interacting Members Gemfibrozil | CYP2C8 | Hypotension | D: Consider therapy modification | Reliability Rating Good | Severity Moderate | Reduce the initial dose of treprostinil extended release tablets to 0.125 mg twice daily, titrating by increments of 0.125 mg every 3 to 4 days, in patients receiving strong CYP2C8 inhibitors. No preemptive dose adjustment is recommended for patients receiving strong CYP2C8 inhibitors with other treprostinil formulations. If combined, monitor patients for increases in treprostinil adverse reactions (eg, hypotension, bleeding, cough, headache) and need for treprostinil dose reduction. | CYP2C8 Inhibitors (Strong) may increase the serum concentration of Treprostinil. |
| Vardenafil / CYP3A4 Inhibitors (Strong) | Vardenafil | CYP3A4 Inhibitors (Strong) Interacting Members Atazanavir, Ceritinib, Clarithromycin, Cobicistat, Darunavir, Idelalisib, Indinavir, Lonafarnib, Lopinavir, MiFEPRIStone, Nefazodone, Nelfinavir, Ombitasvir, Paritaprevir, and Ritonavir, Ombitasvir, Paritaprevir, Ritonavir, and Dasabuvir, Posaconazole, Saquinavir, Telithromycin, Tucatinib, Voriconazole Exceptions Itraconazole, Ketoconazole (Systemic), Ritonavir | CYP3A4 | Hypotension | D: Consider therapy modification | Reliability Rating Good | Severity Major | Levitra (vardenafil) prescribing information recommends limiting the vardenafil dose to a single 2.5 mg dose within a 24-hour period when used with strong CYP3A4 inhibitors. Monitor patients closely for evidence of vardenafil toxicity (eg, hypotension). Staxyn (vardenafil) prescribing information recommends avoiding concomitant use with strong CYP3A4 inhibitors. Some non-US labeling states that the combination of vardenafil and strong CYP3A4 inhibitors is contraindicated. See separate drug interaction monographs for drugs listed as ‘Exceptions’ below. | CYP3A4 Inhibitors (Strong) may increase the serum concentration of Vardenafil. |
| Verapamil / CYP3A4 Inhibitors (Moderate) | Verapamil | CYP3A4 Inhibitors (Moderate) Interacting Members Aprepitant, Berotralstat, Conivaptan, Crizotinib, DilTIAZem, Dronedarone, Duvelisib, Fedratinib, Fluconazole, Fosamprenavir, Fosnetupitant, Grapefruit Juice, Imatinib, Isavuconazonium Sulfate, Lefamulin, Letermovir, Netupitant, Nilotinib, Ribociclib, Schisandra, Verapamil Exception Erythromycin (Systemic) | CYP3A4 | Hypotension | C: Monitor therapy | Reliability Rating Good | Severity Moderate | Monitor for increased verapamil toxicities (eg, hypotension, bradycardia) when combined with moderate CYP3A4 inhibitors. See separate drug interaction monographs for drugs listed as ‘Exceptions’ below. | CYP3A4 Inhibitors (Moderate) may increase the serum concentration of Verapamil. |
| Fostamatinib / CYP3A4 Inhibitors (Strong) | Fostamatinib | CYP3A4 Inhibitors (Strong) Interacting Members Atazanavir, Ceritinib, Clarithromycin, Cobicistat, Darunavir, Idelalisib, Indinavir, Itraconazole, Ketoconazole (Systemic), Lonafarnib, Lopinavir, MiFEPRIStone, Nefazodone, Nelfinavir, Ombitasvir, Paritaprevir, and Ritonavir, Ombitasvir, Paritaprevir, Ritonavir, and Dasabuvir, Posaconazole, Ritonavir, Saquinavir, Telithromycin, Tucatinib, Voriconazole | CYP3A4 | Hypertension | C: Monitor therapy | Reliability Rating Good | Severity Moderate | Monitor patients for increased fostamatinib toxicities when combined with strong CYP3A4 inhibitors. Specifically monitor for toxicities that may require fostamatinib dose reductions (ie, hypertension, hepatoxicity, diarrhea, neutropenia) when these agents are combined. | CYP3A4 Inhibitors (Strong) may increase serum concentrations of the active metabolite(s) of Fostamatinib. |
| Moclobemide / CYP2C19 Inhibitors (Moderate) | Moclobemide | CYP2C19 Inhibitors (Moderate) Interacting Members Cannabidiol, Cenobamate, Fedratinib, FLUoxetine, FluvoxaMINE, Moclobemide, Stiripentol, Voriconazole | CYP2C19 | Hypertension | C: Monitor therapy | Reliability Rating Good | Severity Minor | Monitor for increased moclobemide effects and toxicities (eg, serotonin syndrome, hypertension, dry mouth) if combined with moderate CYP2C19 inhibitors. | CYP2C19 Inhibitors (Moderate) may increase the serum concentration of Moclobemide. |
| Ripretinib / CYP3A4 Inhibitors (Strong) | Ripretinib | CYP3A4 Inhibitors (Strong) Interacting Members Atazanavir, Ceritinib, Clarithromycin, Cobicistat, Darunavir, Idelalisib, Indinavir, Itraconazole, Ketoconazole (Systemic), Lonafarnib, Lopinavir, MiFEPRIStone, Nefazodone, Nelfinavir, Ombitasvir, Paritaprevir, and Ritonavir, Ombitasvir, Paritaprevir, Ritonavir, and Dasabuvir, Posaconazole, Ritonavir, Saquinavir, Telithromycin, Tucatinib, Voriconazole | CYP3A4 | Hypertension | C: Monitor therapy | Reliability Rating Good | Severity Moderate | Monitor for increased ripretinib toxicities (eg, hypertension, arthralgias, left ventricular systolic dysfunction) when combined with strong CYP3A4 inhibitors. | CYP3A4 Inhibitors (Strong) may increase the serum concentration of Ripretinib. |
| Tucatinib / CYP2C8 Inhibitors (Moderate) | Tucatinib | CYP2C8 Inhibitors (Moderate) Interacting Members Clopidogrel, Deferasirox, Leflunomide, Selpercatinib, Teriflunomide | CYP2C8 | Hepatotoxicity | C: Monitor therapy | Reliability Rating Fair: Reported in the prescribing information | Severity Moderate | Close clinical and laboratory monitoring for signs and symptoms of tucatinib toxicity (eg, diarrhea, liver function tests) is warranted during coadministration of tucatinib and moderate CYP2C8 inhibitors. | CYP2C8 Inhibitors (Moderate) may increase the serum concentration of Tucatinib. |
| SUNItinib / CYP3A4 Inhibitors (Strong) | SUNItinib | CYP3A4 Inhibitors (Strong) Interacting Members Atazanavir, Ceritinib, Clarithromycin, Cobicistat, Darunavir, Idelalisib, Indinavir, Itraconazole, Ketoconazole (Systemic), Lonafarnib, Lopinavir, MiFEPRIStone, Nefazodone, Nelfinavir, Ombitasvir, Paritaprevir, and Ritonavir, Ombitasvir, Paritaprevir, Ritonavir, and Dasabuvir, Posaconazole, Ritonavir, Saquinavir, Telithromycin, Tucatinib, Voriconazole | CYP3A4 | Hepatotoxicity | D: Consider therapy modification | Reliability Rating Good | Severity Major | Avoid concurrent use of sunitinib with strong CYP3A4 inhibitors whenever possible. If such a combination cannot be avoided, decrease the sunitinib dose to a minimum of 37.5 mg daily for 4 weeks on treatment, then 2 weeks off when treating gastrointestinal stromal tumor (GIST) or renal cell carcinoma (RCC). Decrease the sunitinib dose to a minimum of 25 mg daily when treating pancreatic neuroendocrine tumor (PNET). Monitor patients carefully for both evidence of sunitinib toxicity and reduced sunitinib efficacy. | CYP3A4 Inhibitors (Strong) may increase the serum concentration of SUNItinib. |
| Perhexiline / CYP2D6 Inhibitors (Moderate) | Perhexiline | CYP2D6 Inhibitors (Moderate) Interacting Members Abiraterone Acetate, Cinacalcet, Darifenacin, Darunavir, DULoxetine, Givosiran, Lorcaserin (Withdrawn From US Market), Mirabegron, Perhexiline, Rolapitant, Terbinafine (Systemic), Thioridazine | CYP2D6 | Hepatotoxicity | C: Monitor therapy | Reliability Rating Good | Severity Moderate | Monitor for increased perhexiline serum concentrations and toxicities (eg, hypoglycemia, neuropathy, liver dysfunction) when combined with moderate CYP2D6 inhibitors. Perhexiline dose reductions may be necessary. | CYP2D6 Inhibitors (Moderate) may increase the serum concentration of Perhexiline. |
| Perhexiline / CYP2D6 Inhibitors (Strong) | Perhexiline | CYP2D6 Inhibitors (Strong) Interacting Members BuPROPion, Dacomitinib, FLUoxetine, PARoxetine, QuiNIDine, Quinidine (Non-Therapeutic), Tipranavir | CYP2D6 | Hepatotoxicity | C: Monitor therapy | Reliability Rating Good | Severity Moderate | Monitor for increased perhexiline serum concentrations and toxicities (eg, hypoglycemia, neuropathy, liver dysfunction) when combined with strong CYP2D6 inhibitors. Perhexiline dose reductions may be necessary. | CYP2D6 Inhibitors (Strong) may increase the serum concentration of Perhexiline. |
| Bosentan / CYP3A4 Inhibitors (Strong) | Bosentan | CYP3A4 Inhibitors (Strong) Interacting Members Atazanavir, Ceritinib, Clarithromycin, Cobicistat, Darunavir, Idelalisib, Indinavir, Itraconazole, Ketoconazole (Systemic), Lonafarnib, Lopinavir, MiFEPRIStone, Nefazodone, Nelfinavir, Ombitasvir, Paritaprevir, and Ritonavir, Ombitasvir, Paritaprevir, Ritonavir, and Dasabuvir, Posaconazole, Ritonavir, Saquinavir, Telithromycin, Tucatinib, Voriconazole | CYP3A4 | Hepatotoxicity | C: Monitor therapy | Reliability Rating Good | Severity Moderate | Monitor for increased bosentan toxicities (ie, hepatotoxicity) when bosentan is combined with a strong CYP3A4 inhibitor. Use of bosentan with a strong CYP3A4 inhibitor and a CYP2C9 inhibitor is not recommended. | CYP3A4 Inhibitors (Strong) may increase the serum concentration of Bosentan. |
| Capmatinib / CYP3A4 Inhibitors (Strong) | Capmatinib | CYP3A4 Inhibitors (Strong) Interacting Members Atazanavir, Ceritinib, Clarithromycin, Cobicistat, Darunavir, Idelalisib, Indinavir, Itraconazole, Ketoconazole (Systemic), Lonafarnib, Lopinavir, MiFEPRIStone, Nefazodone, Nelfinavir, Ombitasvir, Paritaprevir, and Ritonavir, Ombitasvir, Paritaprevir, Ritonavir, and Dasabuvir, Posaconazole, Ritonavir, Saquinavir, Telithromycin, Tucatinib, Voriconazole | CYP3A4 | Hepatotoxicity | C: Monitor therapy | Reliability Rating Good | Severity Moderate | Monitor for increased capmatinib toxicities (eg, hepatotoxicity, interstitial lung disease) when combined with strong CYP3A4 inhibitors. | CYP3A4 Inhibitors (Strong) may increase the serum concentration of Capmatinib. |
| Fostamatinib / CYP3A4 Inhibitors (Strong) | Fostamatinib | CYP3A4 Inhibitors (Strong) Interacting Members Atazanavir, Ceritinib, Clarithromycin, Cobicistat, Darunavir, Idelalisib, Indinavir, Itraconazole, Ketoconazole (Systemic), Lonafarnib, Lopinavir, MiFEPRIStone, Nefazodone, Nelfinavir, Ombitasvir, Paritaprevir, and Ritonavir, Ombitasvir, Paritaprevir, Ritonavir, and Dasabuvir, Posaconazole, Ritonavir, Saquinavir, Telithromycin, Tucatinib, Voriconazole | CYP3A4 | Hepatotoxicity | C: Monitor therapy | Reliability Rating Good | Severity Moderate | Monitor patients for increased fostamatinib toxicities when combined with strong CYP3A4 inhibitors. Specifically monitor for toxicities that may require fostamatinib dose reductions (ie, hypertension, hepatoxicity, diarrhea, neutropenia) when these agents are combined. | CYP3A4 Inhibitors (Strong) may increase serum concentrations of the active metabolite(s) of Fostamatinib. |
| Pioglitazone / CYP2C8 Inhibitors (Moderate) | Pioglitazone | CYP2C8 Inhibitors (Moderate) Interacting Members Clopidogrel, Deferasirox, Leflunomide, Selpercatinib, Teriflunomide | CYP2C8 | Hepatotoxicity | C: Monitor therapy | Reliability Rating Good | Severity Moderate | Monitor patients for increased pioglitazone effects (eg, decreased blood glucose, evidence of edema or hepatotoxicity) when combined with moderate CYP2C8 inhibitors. | CYP2C8 Inhibitors (Moderate) may increase the serum concentration of Pioglitazone. |
| Pioglitazone / CYP2C8 Inhibitors (Strong) | Pioglitazone | CYP2C8 Inhibitors (Strong) Interacting Members Gemfibrozil* | CYP2C8 | Hepatotoxicity | D: Consider therapy modification | Reliability Rating Good | Severity Moderate | Limit the pioglitazone dose to 15 mg daily and monitor for increased pioglitazone toxicities (eg, decreased blood glucose, evidence of edema or hepatotoxicity) when used in combination with strong CYP2C8 inhibitors. | CYP2C8 Inhibitors (Strong) may increase the serum concentration of Pioglitazone. |
| Diclofenac (Systemic) / CYP2C9 Inhibitors (Moderate) | Diclofenac (Systemic) | CYP2C9 Inhibitors (Moderate) Interacting Members Fluconazole, MiFEPRIStone, 尼替西农 | CYP2C9 | Renal toxicity | C: Monitor therapy | Reliability Rating Good | Severity Moderate | Monitor for increased diclofenac toxicities (eg, gastrointestinal bleeding, renal toxicity) if combined with moderate CYP2C9 inhibitors. A dosage adjustment of diclofenac may be warranted. The prescribing information for the diclofenac and misoprostol combination product recommends a maximum dose of 50 mg twice daily when coadministered with CYP2C9 inhibitors. | CYP2C9 Inhibitors (Moderate) may increase the serum concentration of Diclofenac (Systemic). |
| Flurbiprofen (Systemic) / CYP2C9 Inhibitors (Moderate) | Flurbiprofen (Systemic) | CYP2C9 Inhibitors (Moderate) Interacting Members Fluconazole, MiFEPRIStone, 尼替西农 | CYP2C9 | Renal toxicity | C: Monitor therapy | Reliability Rating Excellent | Severity Moderate | Monitor for increased flurbiprofen toxicities (eg, gastrointestinal bleeding, renal toxicity) if combined with moderate CYP2C9 inhibitors. | CYP2C9 Inhibitors (Moderate) may increase the serum concentration of Flurbiprofen (Systemic). |
| Ifosfamide / CYP3A4 Inducers (Strong) | Ifosfamide | CYP3A4 Inducers (Strong) Interacting Members Apalutamide, CarBAMazepine, Enzalutamide, Fosphenytoin, Lumacaftor and Ivacaftor, Mitotane, PHENobarbital, Phenytoin, Primidone, RifAMPin | CYP3A4 | Renal toxicity | C: Monitor therapy | Reliability Rating Good | Severity Moderate | Use of a strong CYP3A4 inducer may increase metabolism of ifosfamide, potentially increasing or decreasing ifosfamide therapeutic effects as well as increasing the risk of some toxicities (e.g., nephrotoxicity, neurotoxicity). Monitor patients closely for clinical response and toxicity, and adjust dose as needed. | CYP3A4 Inducers (Strong) may increase serum concentrations of the active metabolite(s) of Ifosfamide. CYP3A4 Inducers (Strong) may decrease serum concentrations of the active metabolite(s) of Ifosfamide. |
| Ifosfamide / CYP3A4 Inhibitors (Strong) | Ifosfamide | CYP3A4 Inhibitors (Strong) Interacting Members Atazanavir, Ceritinib, Clarithromycin, Cobicistat, Darunavir, Idelalisib, Indinavir, Itraconazole, Ketoconazole (Systemic), Lonafarnib, Lopinavir, MiFEPRIStone, Nefazodone, Nelfinavir, Ombitasvir, Paritaprevir, and Ritonavir, Ombitasvir, Paritaprevir, Ritonavir, and Dasabuvir, Posaconazole, Ritonavir, Saquinavir, Telithromycin, Tucatinib, Voriconazole | CYP3A4 | Renal toxicity | C: Monitor therapy | Reliability Rating Good | Severity Moderate | Use of a CYP3A4 inhibitor may decrease metabolism of ifosfamide, potentially reducing ifosfamide therapeutic effects as well as altering the risk of some toxicities (e.g., nephrotoxicity, neurotoxicity). | CYP3A4 Inhibitors (Strong) may decrease serum concentrations of the active metabolite(s) of Ifosfamide. |
| Bedaquiline / CYP3A4 Inhibitors (Strong) | Bedaquiline | CYP3A4 Inhibitors (Strong) Interacting Members Atazanavir, Cobicistat, Darunavir, Idelalisib, Indinavir, Itraconazole, Ketoconazole (Systemic), Lonafarnib, MiFEPRIStone, Nefazodone, Nelfinavir, Ombitasvir, Paritaprevir, and Ritonavir, Ombitasvir, Paritaprevir, Ritonavir, and Dasabuvir, Ritonavir, Telithromycin, Tucatinib Exceptions Ceritinib, Clarithromycin, Lopinavir, Posaconazole, Saquinavir, Voriconazole | CYP3A4 | Arrhythmias | D: Consider therapy modification | Reliability Rating Good | Severity Major | Limit concomitant administration of bedaquiline with strong CYP3A4 inhibitors to no more than 14 days, unless the expected benefit of continued administration outweighs the possible risks. Monitor patients for increased toxic effects of bedaquiline (eg, QTc interval prolongation). See separate drug interaction monographs for drugs listed as 'Exceptions' below. | CYP3A4 Inhibitors (Strong) may increase serum concentrations of the active metabolite(s) of Bedaquiline. CYP3A4 Inhibitors (Strong) may increase the serum concentration of Bedaquiline. |
| Bedaquiline / QT-prolonging Strong CYP3A4 Inhibitors (Moderate Risk) | Bedaquiline | QT-prolonging Strong CYP3A4 Inhibitors (Moderate Risk) Interacting Members Ceritinib, Saquinavir, Voriconazole Exception Clarithromycin | QT-prolonging Strong CYP3A4 | Arrhythmias | D: Consider therapy modification | Reliability Rating Good | Severity Major | Consider alternatives to this combination. Coadministration of bedaquiline and strong CYP3A4 inhibitors for more than 14 consecutive days should be avoided unless the benefit of treatment with the drug combination outweighs the risk. If use is necessary, monitor for QTc interval prolongation and arrhythmias (including torsades de pointes). Patients with other risk factors (eg, older age, female sex, bradycardia, hypokalemia, hypomagnesemia, heart disease, and higher drug concentrations) are likely at greater risk for these potentially life-threatening toxicities. See separate drug interaction monographs for drugs listed as ‘Exceptions’ below. | QT-prolonging Strong CYP3A4 Inhibitors (Moderate Risk) may enhance the QTc-prolonging effect of Bedaquiline. QT-prolonging Strong CYP3A4 Inhibitors (Moderate Risk) may increase the serum concentration of Bedaquiline. |
| Cannabis / CYP2C9 Inhibitors (Moderate) | Cannabis | CYP2C9 Inhibitors (Moderate) Interacting Members Fluconazole, MiFEPRIStone, 尼替西农 | CYP2C9 | Arrhythmias | C: Monitor therapy | Reliability Rating Good | Severity Moderate | Monitor patients who use cannabis in combination with moderate CYP2C9 inhibitors closely for enhanced effects of tetrahydrocannabinol (THC) (eg, cognitive effects, sedation, dizziness, tachycardia). No significant interaction has been described, or is expected, between moderate CYP2C9 inhibitors and cannabis strains/products/uses that do not introduce substantial systemic THC concentrations. | CYP2C9 Inhibitors (Moderate) may increase the serum concentration of Cannabis. More specifically, tetrahydrocannabinol serum concentrations may be increased. |
| Cannabis / CYP3A4 Inhibitors (Strong) | Cannabis | CYP3A4 Inhibitors (Strong) Interacting Members Atazanavir, Ceritinib, Clarithromycin, Cobicistat, Darunavir, Idelalisib, Indinavir, Itraconazole, Ketoconazole (Systemic), Lonafarnib, Lopinavir, MiFEPRIStone, Nefazodone, Nelfinavir, Ombitasvir, Paritaprevir, and Ritonavir, Ombitasvir, Paritaprevir, Ritonavir, and Dasabuvir, Posaconazole, Ritonavir, Saquinavir, Telithromycin, Tucatinib, Voriconazole | CYP3A4 | Arrhythmias | C: Monitor therapy | Reliability Rating Good | Severity Moderate | Monitor patients who use cannabis in combination with strong CYP3A4 inhibitors closely for enhanced effects of tetrahydrocannabinol (THC eg, cognitive effects, sedation, dizziness, tachycardia) and cannabidiol (CBD eg, muscle relaxant effects). No significant interaction has been described, or is expected, between strong CYP3A4 inhibitors and cannabis strains/products/uses that do not introduce substantial systemic THC or CBD concentrations. | CYP3A4 Inhibitors (Strong) may increase the serum concentration of Cannabis. More specifically, tetrahydrocannabinol and cannabidiol serum concentrations may be increased. |
| Carvedilol / CYP2C9 Inhibitors (Moderate) | Carvedilol | CYP2C9 Inhibitors (Moderate) Interacting Members Fluconazole, MiFEPRIStone, 尼替西农 | CYP2C9 | Arrhythmias | C: Monitor therapy | Reliability Rating Good | Severity Moderate | Monitor patients closely for signs and symptoms of excessive response to carvedilol (eg, hypotension, bradycardia, orthostasis) when adding a moderate CYP2C9 inhibitor to existing carvedilol therapy or vice versa. | CYP2C9 Inhibitors (Moderate) may increase the serum concentration of Carvedilol. Specifically, concentrations of the S-carvedilol enantiomer may be increased. |
| Carvedilol / CYP2D6 Inhibitors (Strong) | Carvedilol | CYP2D6 Inhibitors (Strong) Interacting Members BuPROPion, Dacomitinib, FLUoxetine, PARoxetine, QuiNIDine, Quinidine (Non-Therapeutic), Tipranavir | CYP2D6 | Arrhythmias | C: Monitor therapy | Reliability Rating Good | Severity Moderate | Monitor patients closely for signs and symptoms of excessive response to carvedilol (eg, hypotension, bradycardia, orthostasis) when used together with strong inhibitors of CYP2D6. | CYP2D6 Inhibitors (Strong) may increase the serum concentration of Carvedilol. |
| Crizotinib / QT-prolonging Moderate CYP3A4 Inhibitors (Moderate Risk) | Crizotinib | QT-prolonging Moderate CYP3A4 Inhibitors (Moderate Risk) Interacting Members Crizotinib, Erythromycin (Systemic), Fluconazole, Nilotinib, Ribociclib | QT-prolonging Moderate CYP3A4 | Arrhythmias | C: Monitor therapy | Reliability Rating Good | Severity Moderate | Monitor for QTc interval prolongation and ventricular arrhythmias (including torsades de pointes) when these drugs are combined. Patients with other risk factors (eg, older age, female sex, bradycardia, hypokalemia, hypomagnesemia, heart disease, and higher drug concentrations) are likely at greater risk for these potentially life-threatening toxicities. | QT-prolonging Moderate CYP3A4 Inhibitors (Moderate Risk) may enhance the QTc-prolonging effect of Crizotinib. QT-prolonging Moderate CYP3A4 Inhibitors (Moderate Risk) may increase the serum concentration of Crizotinib. |
| Delamanid / CYP3A4 Inhibitors (Strong) | Delamanid | CYP3A4 Inhibitors (Strong) Interacting Members Atazanavir, Cobicistat, Darunavir, Idelalisib, Indinavir, Itraconazole, Ketoconazole (Systemic), Lonafarnib, Lopinavir, MiFEPRIStone, Nefazodone, Nelfinavir, Ombitasvir, Paritaprevir, and Ritonavir, Ombitasvir, Paritaprevir, Ritonavir, and Dasabuvir, Posaconazole, Ritonavir, Telithromycin, Tucatinib Exceptions Ceritinib, Clarithromycin, Saquinavir, Voriconazole | CYP3A4 | Arrhythmias | D: Consider therapy modification | Reliability Rating Good | Severity Major | Increase electrocardiogram (ECG) monitoring frequency if delamanid is combined with strong CYP3A4 inhibitors because the risk for QTc interval prolongation may be increased. Continue frequent ECG assessments throughout the full delamanid treatment period. See separate drug interaction monographs for drugs listed as ‘Exceptions’ below. | CYP3A4 Inhibitors (Strong) may increase the serum concentration of Delamanid. |
| DilTIAZem / CYP3A4 Inhibitors (Moderate) | DilTIAZem | CYP3A4 Inhibitors (Moderate) Interacting Members Aprepitant, Berotralstat, Conivaptan, Crizotinib, DilTIAZem, Dronedarone, Duvelisib, Fedratinib, Fluconazole, Fosamprenavir, Fosnetupitant, Grapefruit Juice, Imatinib, Isavuconazonium Sulfate, Lefamulin, Letermovir, Netupitant, Nilotinib, Ribociclib, Schisandra, Verapamil Exception Erythromycin (Systemic) | CYP3A4 | Arrhythmias | C: Monitor therapy | Reliability Rating Good | Severity Minor | Monitor for increased diltiazem effects and toxicities (eg, hypotension, bradycardia) when combined with moderate CYP3A4 inhibitors. See separate drug interaction monographs for drugs listed as ‘Exceptions’ below. | CYP3A4 Inhibitors (Moderate) may increase the serum concentration of DilTIAZem. |
| Dronabinol / CYP2C9 Inhibitors (Moderate) | Dronabinol | CYP2C9 Inhibitors (Moderate) Interacting Members Fluconazole, Nitisinone Exception MiFEPRIStone | CYP2C9 | Arrhythmias | C: Monitor therapy | Reliability Rating Good | Severity Moderate | Monitor patients who use dronabinol in combination with moderate CYP2C9 inhibitors closely for enhanced dronabinol effects (eg, cognitive effects, sedation, dizziness, tachycardia). See separate drug interaction monographs for drugs listed as 'Exceptions' below. | CYP2C9 Inhibitors (Moderate) may increase the serum concentration of Dronabinol. |
| Dronabinol / CYP3A4 Inhibitors (Strong) | Dronabinol | CYP3A4 Inhibitors (Strong) Interacting Members Atazanavir, Ceritinib, Clarithromycin, Cobicistat, Darunavir, Idelalisib, Indinavir, Itraconazole, Ketoconazole (Systemic), Lonafarnib, Lopinavir, MiFEPRIStone, Nefazodone, Nelfinavir, Ombitasvir, Paritaprevir, and Ritonavir, Ombitasvir, Paritaprevir, Ritonavir, and Dasabuvir, Posaconazole, Ritonavir, Saquinavir, Telithromycin, Tucatinib, Voriconazole | CYP3A4 | Arrhythmias | C: Monitor therapy | Reliability Rating Good | Severity Moderate | Monitor patients who use dronabinol in combination with strong CYP3A4 inhibitors closely for enhanced effects of dronabinol (eg, cognitive effects, sedation, dizziness, tachycardia). | CYP3A4 Inhibitors (Strong) may increase the serum concentration of Dronabinol. |
| Gilteritinib / QT-prolonging Strong CYP3A4 Inhibitors (Moderate Risk) | Gilteritinib | QT-prolonging Strong CYP3A4 Inhibitors (Moderate Risk) Interacting Members Ceritinib, Clarithromycin, Saquinavir, Voriconazole | QT-prolonging Strong CYP3A4 | Arrhythmias | D: Consider therapy modification | Reliability Rating Good | Severity Major | Consider alternatives to the use of gilteritinib with strong CYP3A4 inhibitors that prolong the QTc interval whenever possible. If treatment with a strong CYP3A4 inhibitor is required, monitor more closely for evidence of gilteritinib toxicities, including QTc interval prolongation and ventricular arrhythmias. | QT-prolonging Strong CYP3A4 Inhibitors (Moderate Risk) may enhance the QTc-prolonging effect of Gilteritinib. QT-prolonging Strong CYP3A4 Inhibitors (Moderate Risk) may increase the serum concentration of Gilteritinib. |
| GuanFACINE / CYP3A4 Inhibitors (Moderate) | GuanFACINE | CYP3A4 Inhibitors (Moderate) Interacting Members Aprepitant, Berotralstat, Conivaptan, Crizotinib, DilTIAZem, Dronedarone, Duvelisib, Erythromycin (Systemic), Fedratinib, Fluconazole, Fosamprenavir, Fosnetupitant, Grapefruit Juice, Imatinib, Isavuconazonium Sulfate, Lefamulin, Letermovir, Netupitant, Nilotinib, Ribociclib, Schisandra, Verapamil | CYP3A4 | Arrhythmias | D: Consider therapy modification | Reliability Rating Fair: Reported in the prescribing information | Severity Moderate | Reduce the extended-release guanfacine dose by 50% when combined with a moderate CYP3A4 inhibitor. When discontinuing moderate CYP3A4 inhibitor treatment, increase the extended-release guanfacine dose to the recommended dose range. No specific dose adjustments are provided for use with immediate release guanfacine, but patients receiving any formulation of guanfacine with a moderate CYP3A4 inhibitor should be monitored closely for evidence of excessive guanfacine response (eg, hypotension, bradycardia, CNS depression). | CYP3A4 Inhibitors (Moderate) may increase the serum concentration of GuanFACINE. |
| GuanFACINE / CYP3A4 Inhibitors (Strong) | GuanFACINE | CYP3A4 Inhibitors (Strong) Interacting Members Atazanavir, Ceritinib, Clarithromycin, Cobicistat, Darunavir, Idelalisib, Indinavir, Itraconazole, Ketoconazole (Systemic), Lonafarnib, Lopinavir, MiFEPRIStone, Nefazodone, Nelfinavir, Ombitasvir, Paritaprevir, and Ritonavir, Ombitasvir, Paritaprevir, Ritonavir, and Dasabuvir, Posaconazole, Ritonavir, Saquinavir, Telithromycin, Tucatinib, Voriconazole | CYP3A4 | Arrhythmias | D: Consider therapy modification | Reliability Rating Good | Severity Moderate | Reduce the extended-release guanfacine dose by 50% when combined with a strong CYP3A4 inhibitor. When discontinuing strong CYP3A4 inhibitor treatment, increase the extended-release guanfacine dose to the recommended dose range. No specific dose adjustments are provided for use with immediate release guanfacine, but patients receiving any formulation of guanfacine with a strong CYP3A4 inhibitor should be monitored closely for evidence of excessive guanfacine response (eg, hypotension, bradycardia, CNS depression). | CYP3A4 Inhibitors (Strong) may increase the serum concentration of GuanFACINE. |
| Iloperidone / CYP2D6 Inhibitors (Strong) | Iloperidone | CYP2D6 Inhibitors (Strong) Interacting Members BuPROPion, Dacomitinib, FLUoxetine, PARoxetine, QuiNIDine, Quinidine (Non-Therapeutic), Tipranavir | CYP2D6 | Arrhythmias | D: Consider therapy modification | Reliability Rating Good | Severity Moderate | Reduce iloperidone dose by half when administered with a strong CYP2D6 inhibitor and monitor for increased iloperidone toxicities, including QTc interval prolongation and arrhythmias. | CYP2D6 Inhibitors (Strong) may increase serum concentrations of the active metabolite(s) of Iloperidone. Specifically, concentrations of the metabolite P88 may be increased. CYP2D6 Inhibitors (Strong) may decrease serum concentrations of the active metabolite(s) of Iloperidone. Specifically, concentrations of the metabolite P95 may be decreased. CYP2D6 Inhibitors (Strong) may increase the serum concentration of Iloperidone. |
| Lofexidine / CYP2D6 Inhibitors (Strong) | Lofexidine | CYP2D6 Inhibitors (Strong) Interacting Members BuPROPion, Dacomitinib, FLUoxetine, PARoxetine, Quinidine (Non-Therapeutic), Tipranavir Exception QuiNIDine | CYP2D6 | Arrhythmias | C: Monitor therapy | Reliability Rating Good | Severity Moderate | Clinical monitoring for signs and symptoms of lofexidine toxicity (eg, orthostatic hypotension, bradycardia) is warranted when coadministering lofexidine and strong CYP2D6 inhibitors. See separate drug interaction monographs for drugs listed as ‘Exceptions’ below. | CYP2D6 Inhibitors (Strong) may increase the serum concentration of Lofexidine. |
| Lonafarnib / CYP3A4 Inhibitors (Weak) | Lonafarnib | CYP3A4 Inhibitors (Weak) Interacting Members ALPRAZolam, Amiodarone, AmLODIPine, Berberine, Bicalutamide, Chlorzoxazone, Cilostazol, Cimetidine, Ciprofloxacin (Systemic), Clotrimazole (Oral), CycloSPORINE (Systemic), Danazol, Delavirdine, Everolimus, FluvoxaMINE, Fosaprepitant, Givosiran, Glecaprevir and Pibrentasvir, Goldenseal, Grazoprevir, Idebenone, Iloperidone, Isoniazid, Istradefylline, Ivacaftor, Lapatinib, Larotrectinib, Levamlodipine, Lomitapide, Lurasidone, Osilodrostat, Palbociclib, PAZOPanib, Peppermint, Piperaquine, Propiverine, Propofol, QuiNIDine, Quinupristin and Dalfopristin, Ranolazine, Resveratrol, Roxithromycin, Rucaparib, Selpercatinib, Tofisopam, Viloxazine, Voxelotor Exception Bitter Orange | CYP3A4 | Arrhythmias | D: Consider therapy modification | Reliability Rating Fair: Reported in the prescribing information | Severity Moderate | Avoid the concurrent use of lonafarnib with weak CYP3A4 inhibitors. If concurrent use is unavoidable, reduce the lonafarnib dose to 115 mg/m2 or continue lonafarnib at a dose of 115 mg/m2. With any combined use, monitor patient closely for evidence of arrhythmia, syncope, palpitations, or similar effects. Bitter orange is specifically contraindicated with lonafarnib, and that interaction is listed as an exception here because it is discussed in a separate interaction monograph. | CYP3A4 Inhibitors (Weak) may increase the serum concentration of Lonafarnib. |
| Loperamide-Loperamide Oxide / CYP3A4 Inhibitors (Strong) | Loperamide-Loperamide Oxide | CYP3A4 Inhibitors (Strong) Interacting Members Atazanavir, Ceritinib, Clarithromycin, Cobicistat, Darunavir, Idelalisib, Indinavir, Itraconazole, Ketoconazole (Systemic), Lonafarnib, Lopinavir, MiFEPRIStone, Nefazodone, Nelfinavir, Ombitasvir, Paritaprevir, and Ritonavir, Ombitasvir, Paritaprevir, Ritonavir, and Dasabuvir, Posaconazole, Ritonavir, Saquinavir, Telithromycin, Tucatinib, Voriconazole | CYP3A4 | Arrhythmias | B: No action needed | Reliability Rating Good | Severity Minor | No action necessary with use of normal doses of these drugs in most patients. Advise patients to avoid use of excessive doses of either drug, particularly in combination, and to promptly report any evidence of possible toxicity including any evidence of arrhythmia. Patients with other risk factors for QT prolongation or ventricular arrhythmias may be at particular risk with such a combination. | CYP3A4 Inhibitors (Strong) may increase the serum concentration of Loperamide-Loperamide Oxide. |
| Metoprolol / CYP2D6 Inhibitors (Moderate) | Metoprolol | CYP2D6 Inhibitors (Moderate) Interacting Members Abiraterone Acetate, Cinacalcet, Darifenacin, Darunavir, DULoxetine, Givosiran, Lorcaserin (Withdrawn From US Market), Mirabegron, Perhexiline, Rolapitant, Terbinafine (Systemic), Thioridazine | CYP2D6 | Arrhythmias | C: Monitor therapy | Reliability Rating Excellent | Severity Moderate | Monitor closely for evidence of excessive response to metoprolol (including, but not necessarily limited to, parameters such as heart rate, PR interval, blood pressure). Metoprolol dose reductions may be necessary. | CYP2D6 Inhibitors (Moderate) may increase the serum concentration of Metoprolol. |
| Metoprolol / CYP2D6 Inhibitors (Strong) | Metoprolol | CYP2D6 Inhibitors (Strong) Interacting Members BuPROPion, Dacomitinib, FLUoxetine, PARoxetine, QuiNIDine, Quinidine (Non-Therapeutic), Tipranavir | CYP2D6 | Arrhythmias | C: Monitor therapy | Reliability Rating Excellent | Severity Moderate | Monitor closely for evidence of excessive response to metoprolol (including, but not necessarily limited to, parameters such as heart rate, PR interval, blood pressure). Metoprolol dose reductions may be necessary. | CYP2D6 Inhibitors (Strong) may increase the serum concentration of Metoprolol. |
| Midostaurin / QT-prolonging Strong CYP3A4 Inhibitors (Moderate Risk) | Midostaurin | QT-prolonging Strong CYP3A4 Inhibitors (Moderate Risk) Interacting Members Ceritinib, Clarithromycin, Saquinavir, Voriconazole | QT-prolonging Strong CYP3A4 | Arrhythmias | D: Consider therapy modification | Reliability Rating Good | Severity Moderate | Consider alternatives to this combination. If use is necessary, monitor for QTc interval prolongation and arrhythmias (including torsades de pointes). Patients with other risk factors (eg, older age, female sex, bradycardia, hypokalemia, hypomagnesemia, heart disease, and higher drug concentrations) are likely at greater risk for these potentially life-threatening toxicities. | QT-prolonging Strong CYP3A4 Inhibitors (Moderate Risk) may enhance the QTc-prolonging effect of Midostaurin. QT-prolonging Strong CYP3A4 Inhibitors (Moderate Risk) may increase the serum concentration of Midostaurin. |
| Nebivolol / CYP2D6 Inhibitors (Strong) | Nebivolol | CYP2D6 Inhibitors (Strong) Interacting Members BuPROPion, Dacomitinib, FLUoxetine, PARoxetine, QuiNIDine, Quinidine (Non-Therapeutic), Tipranavir | CYP2D6 | Arrhythmias | C: Monitor therapy | Reliability Rating Excellent | Severity Moderate | Monitor for increased nebivolol effects and toxicities (eg, hypotension, bradycardia) when combined with strong CYP2D6 inhibitors. Adjust nebivolol doses as needed. | CYP2D6 Inhibitors (Strong) may increase the serum concentration of Nebivolol. |
| Propafenone / CYP2D6 Inhibitors (Strong) | Propafenone | CYP2D6 Inhibitors (Strong) Interacting Members BuPROPion, Dacomitinib, FLUoxetine, PARoxetine, Quinidine (Non-Therapeutic), Tipranavir Exception QuiNIDine | CYP2D6 | Arrhythmias | C: Monitor therapy | Reliability Rating Good | Severity Moderate | Monitor for increased propafenone toxicities, including arrhythmias, when combined with strong CYP2D6 inhibitors. Use of this combination along with a CYP3A4 inhibitor should be avoided. See separate drug interaction monographs for drugs listed as ‘Exceptions’ below. | CYP2D6 Inhibitors (Strong) may increase the serum concentration of Propafenone. |
| Propranolol / CYP2D6 Inhibitors (Strong) | Propranolol | CYP2D6 Inhibitors (Strong) Interacting Members BuPROPion, Dacomitinib, FLUoxetine, PARoxetine, QuiNIDine, Quinidine (Non-Therapeutic), Tipranavir | CYP2D6 | Arrhythmias | C: Monitor therapy | Reliability Rating Good | Severity Moderate | Monitor for increased propranolol effects and toxicities (eg, bradycardia, hypotension) when combined with strong CYP2D6 inhibitors. | CYP2D6 Inhibitors (Strong) may increase the serum concentration of Propranolol. |
| QT-prolonging CYP3A4 Substrates / Posaconazole | Posaconazole | QT-prolonging CYP3A4 Substrates Interacting Members Amiodarone, Astemizole, Bedaquiline, Ceritinib, Cisapride, Clarithromycin, Crizotinib, Dasatinib, Delamanid, Disopyramide, Dofetilide, Domperidone, Dronedarone, Encorafenib, Entrectinib, Gilteritinib, Halofantrine, Haloperidol, Ivosidenib, Methadone, Midostaurin, Nilotinib, Pimozide, QUEtiapine, QuiNIDine, QuiNINE, Ribociclib, Terfenadine | QT-prolonging CYP3A4 | Arrhythmias | X: Avoid combination | Reliability Rating Fair: Reported in the prescribing information | Severity Major | Do not use posaconazole with CYP3A4 substrates that prolong the QT interval. Posaconazole labeling lists this combination as contraindicated. | Posaconazole may increase the serum concentration of QT-prolonging CYP3A4 Substrates. Such increases may lead to a greater risk for proarrhythmic effects and other similar toxicities. |
| QT-prolonging Moderate CYP3A4 Inhibitors (Moderate Risk) / Erythromycin (Systemic) | Erythromycin (Systemic) | QT-prolonging Moderate CYP3A4 Inhibitors (Moderate Risk) Interacting Members Erythromycin (Systemic), Nilotinib, Ribociclib Exceptions Crizotinib, Fluconazole | QT-prolonging Moderate CYP3A4 | Arrhythmias | C: Monitor therapy | Reliability Rating Good | Severity Moderate | Monitor for QTc interval prolongation and ventricular arrhythmias (including torsades de pointes) when these drugs are combined. Patients with other risk factors (eg, older age, female sex, bradycardia, hypokalemia, hypomagnesemia, heart disease, and higher drug concentrations) are likely at greater risk for these potentially life-threatening toxicities. See separate drug interaction monographs for drugs listed as ‘Exceptions’ below. | Erythromycin (Systemic) may enhance the QTc-prolonging effect of QT-prolonging Moderate CYP3A4 Inhibitors (Moderate Risk). QT-prolonging Moderate CYP3A4 Inhibitors (Moderate Risk) may increase the serum concentration of Erythromycin (Systemic). |
| QT-prolonging Moderate CYP3A4 Inhibitors (Moderate Risk) / Ivosidenib | Ivosidenib | QT-prolonging Moderate CYP3A4 Inhibitors (Moderate Risk) Interacting Members Crizotinib, Erythromycin (Systemic), Fluconazole Exceptions Nilotinib, Ribociclib | QT-prolonging Moderate CYP3A4 | Arrhythmias | D: Consider therapy modification | Reliability Rating Good | Severity Major | Avoid concurrent use of moderate CYP3A4 inhibitors together with ivosidenib when possible. If such a combination must be used, monitor for QTc interval prolongation and arrhythmias (including torsades de pointes). Patients with other risk factors (eg, older age, female sex, bradycardia, hypokalemia, hypomagnesemia, heart disease, and higher drug concentrations) are likely at greater risk for these potentially life-threatening toxicities. See separate drug interaction monographs for drugs listed as ‘Exceptions’ below. | Ivosidenib may enhance the QTc-prolonging effect of QT-prolonging Moderate CYP3A4 Inhibitors (Moderate Risk). QT-prolonging Moderate CYP3A4 Inhibitors (Moderate Risk) may increase the serum concentration of Ivosidenib. |
| QT-prolonging Moderate CYP3A4 Inhibitors (Moderate Risk) / Methadone | Methadone | QT-prolonging Moderate CYP3A4 Inhibitors (Moderate Risk) Interacting Members Crizotinib, Erythromycin (Systemic), Fluconazole Exceptions Nilotinib, Ribociclib | QT-prolonging Moderate CYP3A4 | Arrhythmias | D: Consider therapy modification | Reliability Rating Good | Severity Major | Consider alternatives to this combination. If use is necessary, monitor for increased methadone toxicities (eg, respiratory depression, QTc interval prolongation, arrhythmias [including torsades de pointes]). Patients with other risk factors (eg, older age, female sex, bradycardia, hypokalemia, hypomagnesemia, heart disease, and higher drug concentrations) are likely at greater risk for these potentially life-threatening toxicities. See separate drug interaction monographs for drugs listed as ‘Exceptions’ below. | Methadone may enhance the QTc-prolonging effect of QT-prolonging Moderate CYP3A4 Inhibitors (Moderate Risk). QT-prolonging Moderate CYP3A4 Inhibitors (Moderate Risk) may increase the serum concentration of Methadone. |
| QT-prolonging Strong CYP3A4 Inhibitors (Moderate Risk) / Ceritinib | Ceritinib | QT-prolonging Strong CYP3A4 Inhibitors (Moderate Risk) Interacting Members Clarithromycin, Saquinavir, Voriconazole Exception Ceritinib | QT-prolonging Strong CYP3A4 | Arrhythmias | D: Consider therapy modification | Reliability Rating Good | Severity Major | Avoid concurrent use of strong CYP3A4 inhibitors with ceritinib when possible. If such combinations cannot be avoided, the ceritinib dose should be reduced by approximately one-third (rounded to the nearest 150 mg). After the strong CYP3A4 inhibitor is discontinued the prior ceritinib dose should be resumed. Monitor patients for increased ceritinib toxicities, including QTc interval prolongation and ventricular arrhythmias | Ceritinib may enhance the QTc-prolonging effect of QT-prolonging Strong CYP3A4 Inhibitors (Moderate Risk). QT-prolonging Strong CYP3A4 Inhibitors (Moderate Risk) may increase the serum concentration of Ceritinib. |
| QT-prolonging Strong CYP3A4 Inhibitors (Moderate Risk) / Erythromycin (Systemic) | Erythromycin (Systemic) | QT-prolonging Strong CYP3A4 Inhibitors (Moderate Risk) Interacting Members Ceritinib, Clarithromycin, Voriconazole Exception Saquinavir | QT-prolonging Strong CYP3A4 | Arrhythmias | C: Monitor therapy | Reliability Rating Good | Severity Moderate | Monitor for QTc interval prolongation and ventricular arrhythmias (including torsades de pointes) when these drugs are combined. Patients with other risk factors (eg, older age, female sex, bradycardia, hypokalemia, hypomagnesemia, heart disease, and higher drug concentrations) are likely at greater risk for these potentially life-threatening toxicities. See separate drug interaction monographs for drugs listed as ‘Exceptions’ below. | Erythromycin (Systemic) may enhance the QTc-prolonging effect of QT-prolonging Strong CYP3A4 Inhibitors (Moderate Risk). QT-prolonging Strong CYP3A4 Inhibitors (Moderate Risk) may increase the serum concentration of Erythromycin (Systemic). |
| Haloperidol / CYP2D6 Inhibitors (Strong) | Haloperidol | CYP2D6 Inhibitors (Strong) Interacting Members BuPROPion, Dacomitinib, FLUoxetine, PARoxetine, Quinidine (Non-Therapeutic), Tipranavir Exception QuiNIDine | CYP2D6 | Arrhythmias | C: Monitor therapy | Reliability Rating Good | Severity Moderate | Monitor patients for increased haloperidol concentrations and toxicities, including QT prolongation or seizures, when combined with strong CYP2D6 inhibitors. See separate drug interaction monographs for drugs listed as ‘Exceptions’ below. | CYP2D6 Inhibitors (Strong) may increase the serum concentration of Haloperidol. |
| Haloperidol / CYP3A4 Inhibitors (Strong) | Haloperidol | CYP3A4 Inhibitors (Strong) Interacting Members Atazanavir, Cobicistat, Darunavir, Idelalisib, Indinavir, Itraconazole, Ketoconazole (Systemic), Lonafarnib, Lopinavir, MiFEPRIStone, Nefazodone, Nelfinavir, Ombitasvir, Paritaprevir, and Ritonavir, Ombitasvir, Paritaprevir, Ritonavir, and Dasabuvir, Posaconazole, Ritonavir, Telithromycin, Tucatinib Exceptions Ceritinib, Clarithromycin, Saquinavir, Voriconazole | CYP3A4 | Arrhythmias | C: Monitor therapy | Reliability Rating Good | Severity Moderate | Monitor patients for increased haloperidol concentrations and toxicities, including QT prolongation or seizures, when combined with strong CYP3A4 inhibitors. See separate drug interaction monographs for drugs listed as ‘Exceptions’ below. | CYP3A4 Inhibitors (Strong) may increase the serum concentration of Haloperidol. |
| QT-prolonging Strong CYP3A4 Inhibitors (Moderate Risk) / Haloperidol | Haloperidol | QT-prolonging Strong CYP3A4 Inhibitors (Moderate Risk) Interacting Members Ceritinib, Clarithromycin, Voriconazole Exception Saquinavir | QT-prolonging Strong CYP3A4 | Arrhythmias | C: Monitor therapy | Reliability Rating Good | Severity Moderate | Monitor for QTc interval prolongation and ventricular arrhythmias (including torsades de pointes) when these drugs are combined. Patients with other risk factors (eg, older age, female sex, bradycardia, hypokalemia, hypomagnesemia, heart disease, and higher drug concentrations) or those using IV forms of haloperidol are likely at greater risk for these potentially life-threatening toxicities. See separate drug interaction monographs for drugs listed as ‘Exceptions’ below. | Haloperidol may enhance the QTc-prolonging effect of QT-prolonging Strong CYP3A4 Inhibitors (Moderate Risk). QT-prolonging Strong CYP3A4 Inhibitors (Moderate Risk) may increase the serum concentration of Haloperidol. |
| QT-prolonging Strong CYP3A4 Inhibitors (Moderate Risk) / Ivosidenib | Ivosidenib | QT-prolonging Strong CYP3A4 Inhibitors (Moderate Risk) Interacting Members Ceritinib, Saquinavir, Voriconazole Exception Clarithromycin | QT-prolonging Strong CYP3A4 | Arrhythmias | D: Consider therapy modification | Reliability Rating Good | Severity Major | Avoid concurrent use of strong CYP3A4 inhibitors together with ivosidenib when possible. If such a combination must be used, reduce the ivosidenib dose to 250 mg once daily. Following discontinuation of the strong CYP3A4 inhibitor, resume the recommended 500 mg once daily dose of ivosidenib only after a period of time equal to 5 half-lives of the strong inhibitor has elapsed. Additionally, due to the risks for QT prolongation with this combination, monitor for QTc interval prolongation and arrhythmias (including torsades de pointes). Patients with other risk factors (eg, older age, female sex, bradycardia, hypokalemia, hypomagnesemia, heart disease, and higher drug concentrations) are likely at greater risk for these potentially life-threatening toxicities. See separate drug interaction monographs for drugs listed as ‘Exceptions’ below. | Ivosidenib may enhance the QTc-prolonging effect of QT-prolonging Strong CYP3A4 Inhibitors (Moderate Risk). QT-prolonging Strong CYP3A4 Inhibitors (Moderate Risk) may increase the serum concentration of Ivosidenib. |
| QT-prolonging Strong CYP3A4 Inhibitors (Moderate Risk) / Nilotinib | Nilotinib | QT-prolonging Strong CYP3A4 Inhibitors (Moderate Risk) Interacting Members Ceritinib, Clarithromycin, Saquinavir, Voriconazole | QT-prolonging Strong CYP3A4 | Arrhythmias | D: Consider therapy modification | Reliability Rating Good | Severity Major | Avoid concomitant use of nilotinib and strong CYP3A4 inhibitors that prolong the QTc interval whenever possible. If treatment with a strong CYP3A4 inhibitor is required, interruption of nilotinib treatment is recommended. If coadministration is necessary, decrease the nilotinib dose to 300 mg once daily for patients with resistant or intolerant Ph+ CML, or to 200 mg once daily for patients with newly diagnosed Ph+ CML in chronic phase. Monitor patients for increased nilotinib toxicities, including QTc interval prolongation and ventricular arrhythmias. | Nilotinib may enhance the QTc-prolonging effect of QT-prolonging Strong CYP3A4 Inhibitors (Moderate Risk). QT-prolonging Strong CYP3A4 Inhibitors (Moderate Risk) may increase the serum concentration of Nilotinib. |
| QT-prolonging Strong CYP3A4 Inhibitors (Moderate Risk) / QUEtiapine | QUEtiapine | QT-prolonging Strong CYP3A4 Inhibitors (Moderate Risk) Interacting Members Ceritinib, Clarithromycin, Saquinavir, Voriconazole | QT-prolonging Strong CYP3A4 | Arrhythmias | D: Consider therapy modification | Reliability Rating Good | Severity Major | In patients receiving quetiapine, reduce the quetiapine dose to one-sixth of the regular dose following initiation of these strong CYP3A4 inhibitors. Increase the quetiapine dose by 6-fold following discontinuation of the CYP3A4 inhibitor. In patients receiving these strong CYP3A4 inhibitors, initiate quetiapine at the lowest dose and up-titrate cautiously as needed. Monitor patients for increased quetiapine toxicities, including QTc interval prolongation and ventricular arrhythmias. | QUEtiapine may enhance the QTc-prolonging effect of QT-prolonging Strong CYP3A4 Inhibitors (Moderate Risk). QT-prolonging Strong CYP3A4 Inhibitors (Moderate Risk) may increase the serum concentration of QUEtiapine. |
| QUEtiapine / QT-prolonging Moderate CYP3A4 Inhibitors (Moderate Risk) | QUEtiapine | QT-prolonging Moderate CYP3A4 Inhibitors (Moderate Risk) Interacting Members Crizotinib, Erythromycin (Systemic), Fluconazole, Nilotinib, Ribociclib | QT-prolonging Moderate CYP3A4 | Arrhythmias | C: Monitor therapy | Reliability Rating Good | Severity Moderate | Monitor for QTc interval prolongation and ventricular arrhythmias (including torsades de pointes) when these drugs are combined. Patients with other risk factors (eg, older age, female sex, bradycardia, hypokalemia, hypomagnesemia, heart disease, and higher drug concentrations) are likely at greater risk for these potentially life-threatening toxicities. Monitor for other quetiapine-related toxicities due to increased quetiapine exposure dose reduction may be required. | QT-prolonging Moderate CYP3A4 Inhibitors (Moderate Risk) may enhance the QTc-prolonging effect of QUEtiapine. QT-prolonging Moderate CYP3A4 Inhibitors (Moderate Risk) may increase the serum concentration of QUEtiapine. |
| QT-prolonging Strong CYP3A4 Inhibitors (Moderate Risk) / Ribociclib | Ribociclib | QT-prolonging Strong CYP3A4 Inhibitors (Moderate Risk) Interacting Members Ceritinib, Clarithromycin, Saquinavir, Voriconazole | QT-prolonging Strong CYP3A4 | Arrhythmias | D: Consider therapy modification | Reliability Rating Good | Severity Major Onset Immediate | Avoid concomitant use of ribociclib and strong CYP3A4 inhibitors that prolong the QTc interval whenever possible. If treatment with a strong CYP3A4 inhibitor is required, reduce the ribociclib dose to 400 mg once daily and monitor patients for increased ribociclib toxicities, including QTc interval prolongation and ventricular arrhythmias. Following discontinuation of any strong CYP3A4 inhibitor that is used in combination with ribociclib, the ribociclib dose should be increased to the dose used prior to use of the inhibitor once a period of 5 half-lives of the discontinued inhibitor have passed. | Ribociclib may enhance the QTc-prolonging effect of QT-prolonging Strong CYP3A4 Inhibitors (Moderate Risk). QT-prolonging Strong CYP3A4 Inhibitors (Moderate Risk) may increase the serum concentration of Ribociclib. |
| QT-prolonging Strong CYP3A4 Inhibitors (Moderate Risk) / Toremifene | Toremifene | QT-prolonging Strong CYP3A4 Inhibitors (Moderate Risk) Interacting Members Ceritinib, Clarithromycin, Saquinavir, Voriconazole | QT-prolonging Strong CYP3A4 | Arrhythmias | D: Consider therapy modification | Reliability Rating Good | Severity Major | Avoid concomitant use of toremifene and strong CYP3A4 inhibitors that prolong the QTc interval whenever possible. If treatment with a strong CYP3A4 inhibitor that prolongs the QTc interval is required, interruption of toremifene treatment is recommended. If coadministration is necessary, monitor patients for increased toremifene toxicities, including QTc interval prolongation and ventricular arrhythmias. | Toremifene may enhance the QTc-prolonging effect of QT-prolonging Strong CYP3A4 Inhibitors (Moderate Risk). QT-prolonging Strong CYP3A4 Inhibitors (Moderate Risk) may increase the serum concentration of Toremifene. |
| QT-prolonging Strong CYP3A4 Inhibitors (Moderate Risk) / Vemurafenib | Vemurafenib | QT-prolonging Strong CYP3A4 Inhibitors (Moderate Risk) Interacting Members Ceritinib, Clarithromycin, Saquinavir, Voriconazole | QT-prolonging Strong CYP3A4 | Arrhythmias | D: Consider therapy modification | Reliability Rating Good | Severity Moderate | Avoid concomitant use of vemurafenib and strong CYP3A4 inhibitors that prolong the QTc interval whenever possible. If coadministration is required, monitor patients for increased vemurafenib toxicities, including QTc interval prolongation and ventricular arrhythmias, and consider a vemurafenib dose reduction if clinically indicated. | Vemurafenib may enhance the QTc-prolonging effect of QT-prolonging Strong CYP3A4 Inhibitors (Moderate Risk). QT-prolonging Strong CYP3A4 Inhibitors (Moderate Risk) may increase the serum concentration of Vemurafenib. |
| Ripretinib / CYP3A4 Inhibitors (Strong) | Ripretinib | CYP3A4 Inhibitors (Strong) Interacting Members Atazanavir, Ceritinib, Clarithromycin, Cobicistat, Darunavir, Idelalisib, Indinavir, Itraconazole, Ketoconazole (Systemic), Lonafarnib, Lopinavir, MiFEPRIStone, Nefazodone, Nelfinavir, Ombitasvir, Paritaprevir, and Ritonavir, Ombitasvir, Paritaprevir, Ritonavir, and Dasabuvir, Posaconazole, Ritonavir, Saquinavir, Telithromycin, Tucatinib, Voriconazole | CYP3A4 | Arrhythmias | C: Monitor therapy | Reliability Rating Good | Severity Moderate | Monitor for increased ripretinib toxicities (eg, hypertension, arthralgias, left ventricular systolic dysfunction) when combined with strong CYP3A4 inhibitors. | CYP3A4 Inhibitors (Strong) may increase the serum concentration of Ripretinib. |
| Selpercatinib / QT-prolonging Moderate CYP3A4 Inhibitors (Moderate Risk) | Selpercatinib | QT-prolonging Moderate CYP3A4 Inhibitors (Moderate Risk) Interacting Members Crizotinib, Erythromycin (Systemic), Fluconazole, Nilotinib, Ribociclib | QT-prolonging Moderate CYP3A4 | Arrhythmias | D: Consider therapy modification | Reliability Rating Fair: Reported in the prescribing information | Severity Major | Avoid concurrent use of moderate CYP3A4 inhibitors together with selpercatinib when possible. If such a combination must be used, reduce the selpercatinib dose from 120 mg twice daily to 80 mg twice daily, or from 160 mg twice daily to 120 mg twice daily. Additionally, due to the risks for QT prolongation with this combination, monitor more closely for QTc interval prolongation and arrhythmias (including torsades de pointes). Patients with other risk factors (eg, older age, female sex, bradycardia, hypokalemia, hypomagnesemia, heart disease, and higher drug concentrations) are likely at greater risk for these potentially life-threatening toxicities. | QT-prolonging Moderate CYP3A4 Inhibitors (Moderate Risk) may enhance the QTc-prolonging effect of Selpercatinib. QT-prolonging Moderate CYP3A4 Inhibitors (Moderate Risk) may increase the serum concentration of Selpercatinib. |
| Selpercatinib / QT-prolonging Strong CYP3A4 Inhibitors (Moderate Risk) | Selpercatinib | QT-prolonging Strong CYP3A4 Inhibitors (Moderate Risk) Interacting Members Ceritinib, Clarithromycin, Saquinavir, Voriconazole | QT-prolonging Strong CYP3A4 | Arrhythmias | D: Consider therapy modification | Reliability Rating Good | Severity Major | Avoid concurrent use of strong CYP3A4 inhibitors together with selpercatinib when possible. If such a combination must be used, reduce the selpercatinib dose from 120 mg twice daily to 40 mg twice daily, or from 160 mg twice daily to 80 mg twice daily. Additionally, due to the risks for QT prolongation with this combination, monitor more closely for QTc interval prolongation and arrhythmias (including torsades de pointes). Patients with other risk factors (eg, older age, female sex, bradycardia, hypokalemia, hypomagnesemia, heart disease, and higher drug concentrations) are likely at greater risk for these potentially life-threatening toxicities. | QT-prolonging Strong CYP3A4 Inhibitors (Moderate Risk) may enhance the QTc-prolonging effect of Selpercatinib. QT-prolonging Strong CYP3A4 Inhibitors (Moderate Risk) may increase the serum concentration of Selpercatinib. |
| Tetrahydrocannabinol / CYP2C9 Inhibitors (Moderate) | Tetrahydrocannabinol | CYP2C9 Inhibitors (Moderate) Interacting Members Fluconazole, 尼替西农 Exception MiFEPRIStone | CYP2C9 | Arrhythmias | C: Monitor therapy | Reliability Rating Good | Severity Moderate | Monitor patients who use tetrahydrocannabinol (THC) in combination with moderate CYP2C9 inhibitors closely for enhanced THC effects (eg, cognitive effects, sedation, dizziness, tachycardia). See separate drug interaction monographs for drugs listed as 'Exceptions' below. | CYP2C9 Inhibitors (Moderate) may increase the serum concentration of Tetrahydrocannabinol. |
| Tetrahydrocannabinol / CYP3A4 Inhibitors (Strong) | Tetrahydrocannabinol | CYP3A4 Inhibitors (Strong) Interacting Members Atazanavir, Ceritinib, Clarithromycin, Cobicistat, Darunavir, Idelalisib, Indinavir, Itraconazole, Ketoconazole (Systemic), Lonafarnib, Lopinavir, MiFEPRIStone, Nefazodone, Nelfinavir, Ombitasvir, Paritaprevir, and Ritonavir, Ombitasvir, Paritaprevir, Ritonavir, and Dasabuvir, Posaconazole, Ritonavir, Saquinavir, Telithromycin, Tucatinib, Voriconazole | CYP3A4 | Arrhythmias | C: Monitor therapy | Reliability Rating Good | Severity Moderate | Monitor patients who use tetrahydrocannabinol (THC) in combination with strong CYP3A4 inhibitors closely for enhanced effects of THC (eg, cognitive effects, sedation, dizziness, tachycardia). | CYP3A4 Inhibitors (Strong) may increase the serum concentration of Tetrahydrocannabinol. |
| Tetrahydrocannabinol and Cannabidiol / CYP2C9 Inhibitors (Moderate) | Tetrahydrocannabinol and Cannabidiol | CYP2C9 Inhibitors (Moderate) Interacting Members Fluconazole, 尼替西农 Exception MiFEPRIStone | CYP2C9 | Arrhythmias | C: Monitor therapy | Reliability Rating Good | Severity Moderate | Monitor patients who use tetrahydrocannabinol (THC) in combination with moderate CYP2C9 inhibitors closely for enhanced THC effects (eg, cognitive effects, sedation, dizziness, tachycardia). See separate drug interaction monographs for drugs listed as 'Exceptions' below. | CYP2C9 Inhibitors (Moderate) may increase the serum concentration of Tetrahydrocannabinol and Cannabidiol. Specifically, concentrations of tetrahydrocannabinol may be increased. |
| Theophylline Derivatives / CYP1A2 Inhibitors (Moderate) | Acebrophylline, Aminophylline, Dyphylline, Theophylline | CYP1A2 Inhibitors (Moderate) Interacting Members Capmatinib, Ciprofloxacin (Systemic), Deferasirox, Enoxacin, Givosiran, Methoxsalen (Systemic), Mexiletine, Rucaparib, Stiripentol, Thiabendazole, Vemurafenib | CYP1A2 | Arrhythmias | D: Consider therapy modification | Reliability Rating Excellent | Severity Major | Due to the potential severity of theophylline toxicity, consider avoiding the concomitant use of theophylline derivatives and moderate CYP1A2 inhibitors. If coadministration is necessary, monitor for increased theophylline serum concentrations and toxicities (eg, agitation, headache, tachycardia, vomiting, seizures) when combined. Theophylline dose reductions will likely be required. | CYP1A2 Inhibitors (Moderate) may increase the serum concentration of Theophylline Derivatives. |
| Theophylline Derivatives / CYP1A2 Inhibitors (Strong) | Acebrophylline, Aminophylline, Dyphylline, Theophylline | CYP1A2 Inhibitors (Strong) Interacting Members FluvoxaMINE Exception Viloxazine | CYP1A2 | Arrhythmias | D: Consider therapy modification | Reliability Rating Excellent | Severity Major | Due to the potential severity of theophylline toxicity, consider avoiding the concomitant use of theophylline derivatives and strong CYP1A2 inhibitors. If coadministration is necessary, consider an empiric theophylline dose reduction to one-third of the original theophylline dose. Monitor for increased theophylline serum concentrations and toxicities (eg, agitation, headache, tachycardia, vomiting, seizures) when combined.  See separate drug interaction monographs for drugs listed as ‘Exceptions’ below. | CYP1A2 Inhibitors (Strong) may increase the serum concentration of Theophylline Derivatives. |
| Theophylline Derivatives / CYP1A2 Inhibitors (Weak) | Acebrophylline, Aminophylline, Dyphylline, Theophylline | CYP1A2 Inhibitors (Weak) Interacting Members Acyclovir (Systemic), Caffeine, Cannabidiol, Cimetidine, Cola-Containing Drinks, Dipyrone, Disulfiram, Elagolix, Estradiol, and Norethindrone, Estradiol (Systemic), Estradiol (Topical), Estrogens (Conjugated A/Synthetic), Estrogens (Conjugated/Equine, Systemic), Estrogens (Conjugated/Equine, Topical), Ethinyl Estradiol, Glecaprevir and Pibrentasvir, Interferon Alfa-2b, Kola Nut, Mestranol, Obeticholic Acid, Osilodrostat, Pefloxacin, Peginterferon Alfa-2a, Peginterferon Alfa-2b, Pipemidic Acid, Propafenone, Propranolol, Simeprevir, Ticlopidine, ValACYclovir, Verapamil, Zileuton | CYP1A2 | Arrhythmias | C: Monitor therapy | Reliability Rating Excellent | Severity Moderate | Monitor for increased theophylline serum concentrations and toxicities (eg, agitation, headache, tachycardia, vomiting) when theophylline derivatives are combined with weak CYP1A2 inhibitors. Theophylline dose reductions may be required. | CYP1A2 Inhibitors (Weak) may increase the serum concentration of Theophylline Derivatives. |
| Timolol (Ophthalmic) / CYP2D6 Inhibitors (Strong) | Timolol (Ophthalmic) | CYP2D6 Inhibitors (Strong) Interacting Members BuPROPion, Dacomitinib, FLUoxetine, PARoxetine, QuiNIDine, Quinidine (Non-Therapeutic), Tipranavir | CYP2D6 | Arrhythmias | C: Monitor therapy | Reliability Rating Good | Severity Moderate | Monitor closely for evidence of systemic beta-blocker effects, including but not limited to orthostatic hypotension, bradycardia, and exercise intolerance. | CYP2D6 Inhibitors (Strong) may increase the serum concentration of Timolol (Ophthalmic). |
| Timolol (Systemic) / CYP2D6 Inhibitors (Strong) | Timolol (Systemic) | CYP2D6 Inhibitors (Strong) Interacting Members BuPROPion, Dacomitinib, FLUoxetine, PARoxetine, QuiNIDine, Quinidine (Non-Therapeutic), Tipranavir | CYP2D6 | Arrhythmias | C: Monitor therapy | Reliability Rating Good | Severity Moderate | Monitor for increased timolol effects and toxicities (eg, bradycardia, hypotension) when combined with strong CYP2D6 inhibitors. | CYP2D6 Inhibitors (Strong) may increase the serum concentration of Timolol (Systemic). |
| TiZANidine / CYP1A2 Inhibitors (Moderate) | TiZANidine | CYP1A2 Inhibitors (Moderate) Interacting Members Capmatinib, Deferasirox, Enoxacin, Givosiran, Methoxsalen (Systemic), Mexiletine, Rucaparib, Stiripentol, Thiabendazole, Vemurafenib Exception Ciprofloxacin (Systemic) | CYP1A2 | Arrhythmias | D: Consider therapy modification | Reliability Rating Good | Severity Moderate | Avoid the use of tizanidine with moderate CYP1A2 inhibitors when possible. If combined use cannot be avoided, initiate tizanidine at an adult dose of 2 mg and increase in 2 to 4 mg increments based on patient response. Monitor for increased effects of tizanidine, including adverse reactions (eg, hypotension, bradycardia, drowsiness). Drugs listed as exceptions are discussed in separate interaction monographs. | CYP1A2 Inhibitors (Moderate) may increase the serum concentration of TiZANidine. |
| TiZANidine / CYP1A2 Inhibitors (Strong) | TiZANidine | CYP1A2 Inhibitors (Strong) Interacting Members FluvoxaMINE*, Viloxazine | CYP1A2 | Arrhythmias | X: Avoid combination | Reliability Rating Good | Severity Major | Avoid concomitant use of tizanidine and strong CYP1A2 inhibitors. US prescribing information states that combined use is contraindicated. | CYP1A2 Inhibitors (Strong) may increase the serum concentration of TiZANidine. |
| TiZANidine / CYP1A2 Inhibitors (Weak) | TiZANidine | CYP1A2 Inhibitors (Weak) Interacting Members Acyclovir (Systemic), Caffeine, Cannabidiol, Cimetidine, Cola-Containing Drinks, Dipyrone, Disulfiram, Elagolix, Estradiol, and Norethindrone, Estradiol (Systemic), Estradiol (Topical), Estrogens (Conjugated A/Synthetic), Estrogens (Conjugated/Equine, Systemic), Estrogens (Conjugated/Equine, Topical), Ethinyl Estradiol, Glecaprevir and Pibrentasvir, Interferon Alfa-2b, Kola Nut, Mestranol, Obeticholic Acid, Osilodrostat, Pefloxacin, Peginterferon Alfa-2a, Peginterferon Alfa-2b, Pipemidic Acid, Propafenone, Propranolol, Simeprevir, Ticlopidine, ValACYclovir, Verapamil, Zileuton | CYP1A2 | Arrhythmias | D: Consider therapy modification | Reliability Rating Good | Severity Major | Avoid the use of tizanidine with weak CYP1A2 inhibitors when possible. If combined use cannot be avoided, initiate tizanidine at an adult dose of 2 mg and increase in 2 to 4 mg increments based on patient response. Monitor for increased effects of tizanidine, including adverse reactions (eg, hypotension, bradycardia, drowsiness). | CYP1A2 Inhibitors (Weak) may increase the serum concentration of TiZANidine. |
| Upadacitinib / CYP3A4 Inhibitors (Strong) | Upadacitinib | CYP3A4 Inhibitors (Strong) Interacting Members Atazanavir, Ceritinib, Clarithromycin, Cobicistat, Darunavir, Idelalisib, Indinavir, Itraconazole, Ketoconazole (Systemic), Lonafarnib, Lopinavir, MiFEPRIStone, Nefazodone, Nelfinavir, Ombitasvir, Paritaprevir, and Ritonavir, Ombitasvir, Paritaprevir, Ritonavir, and Dasabuvir, Posaconazole, Ritonavir, Saquinavir, Telithromycin, Tucatinib, Voriconazole | CYP3A4 | Arrhythmias | C: Monitor therapy | Reliability Rating Good | Severity Moderate | Monitor for increased upadacitinib toxicities (eg, infection, nausea, signs and symptoms of blood clot) if combined with strong CYP3A4 inhibitors. | CYP3A4 Inhibitors (Strong) may increase the serum concentration of Upadacitinib. |
| Verapamil / CYP3A4 Inhibitors (Moderate) | Verapamil | CYP3A4 Inhibitors (Moderate) Interacting Members Aprepitant, Berotralstat, Conivaptan, Crizotinib, DilTIAZem, Dronedarone, Duvelisib, Fedratinib, Fluconazole, Fosamprenavir, Fosnetupitant, Grapefruit Juice, Imatinib, Isavuconazonium Sulfate, Lefamulin, Letermovir, Netupitant, Nilotinib, Ribociclib, Schisandra, Verapamil Exception Erythromycin (Systemic) | CYP3A4 | Arrhythmias | C: Monitor therapy | Reliability Rating Good | Severity Moderate | Monitor for increased verapamil toxicities (eg, hypotension, bradycardia) when combined with moderate CYP3A4 inhibitors. See separate drug interaction monographs for drugs listed as ‘Exceptions’ below. | CYP3A4 Inhibitors (Moderate) may increase the serum concentration of Verapamil. |
| Halofantrine / QT-prolonging Strong CYP3A4 Inhibitors (Moderate Risk) | Halofantrine | QT-prolonging Strong CYP3A4 Inhibitors (Moderate Risk) Interacting Members Ceritinib, Clarithromycin, Saquinavir, Voriconazole | QT-prolonging Strong CYP3A4 | Arrhythmias | X: Avoid combination | Reliability Rating Good | Severity Moderate | Avoid concomitant use of QT-prolonging Strong CYP3A4 Inhibitors (Moderate Risk) and Halofantrine. | QT-prolonging Strong CYP3A4 Inhibitors (Moderate Risk) may enhance the QTc-prolonging effect of Halofantrine. QT-prolonging Strong CYP3A4 Inhibitors (Moderate Risk) may increase the serum concentration of Halofantrine. |
| Halofantrine / CYP3A4 Inhibitors (Strong) | Halofantrine | CYP3A4 Inhibitors (Strong) Interacting Members Atazanavir, Cobicistat, Darunavir, Idelalisib, Indinavir, Itraconazole, Ketoconazole (Systemic), Lonafarnib, Lopinavir, MiFEPRIStone, Nefazodone, Nelfinavir, Ombitasvir, Paritaprevir, and Ritonavir, Ombitasvir, Paritaprevir, Ritonavir, and Dasabuvir, Posaconazole, Ritonavir, Telithromycin, Tucatinib Exceptions Ceritinib, Clarithromycin, Saquinavir, Voriconazole | CYP3A4 | Arrhythmias | D: Consider therapy modification | Reliability Rating Good | Severity Moderate | Consider alternatives to this combination whenever possible. If combined, monitor closely for halofantrine toxicities, including QTc interval prolongation. See separate drug interaction monographs for drugs listed as ‘Exceptions’ below. | CYP3A4 Inhibitors (Strong) may increase the serum concentration of Halofantrine. |
| Halofantrine / CYP3A4 Inhibitors (Moderate) | Halofantrine | CYP3A4 Inhibitors (Moderate) Interacting Members Aprepitant, Berotralstat, Conivaptan, DilTIAZem, Dronedarone, Duvelisib, Fedratinib, Fosamprenavir, Fosnetupitant, Grapefruit Juice, Imatinib, Isavuconazonium Sulfate, Lefamulin, Letermovir, Netupitant, Schisandra, Verapamil Exceptions Crizotinib, Erythromycin (Systemic), Fluconazole, Nilotinib, Ribociclib | CYP3A4 | Arrhythmias | C: Monitor therapy | Reliability Rating Good | Severity Moderate | Extreme caution, with possibly increased monitoring of cardiac status (eg, ECG), should be used with concurrent use of halofantrine with any moderate CYP3A4 inhibitor(s). See separate drug interaction monographs for drugs listed as ‘Exceptions’ below. | CYP3A4 Inhibitors (Moderate) may increase the serum concentration of Halofantrine. |
| Cisapride / QT-prolonging Strong CYP3A4 Inhibitors (Moderate Risk) | Cisapride | QT-prolonging Strong CYP3A4 Inhibitors (Moderate Risk) Interacting Members Ceritinib, Clarithromycin, Saquinavir, Voriconazole | QT-prolonging Strong CYP3A4 | Arrhythmias | X: Avoid combination | Reliability Rating Good | Severity Major | Avoid concomitant use of cisapride and QT-prolonging Strong CYP3A4 Inhibitors (Moderate Risk). Concomitant use of cisapride with macrolides, protease inhibitors, and azoles is contraindicated. | QT-prolonging Strong CYP3A4 Inhibitors (Moderate Risk) may enhance the QTc-prolonging effect of Cisapride. QT-prolonging Strong CYP3A4 Inhibitors (Moderate Risk) may increase the serum concentration of Cisapride. |
| Citalopram / CYP2C19 Inhibitors (Moderate) | Citalopram | CYP2C19 Inhibitors (Moderate) Interacting Members Cannabidiol, Cenobamate, Fedratinib, Stiripentol Exceptions FLUoxetine, FluvoxaMINE, Moclobemide, Voriconazole | CYP2C19 | Arrhythmias | D: Consider therapy modification | Reliability Rating Good | Severity Moderate | Limit citalopram dose to a maximum of 20 mg/day if used with a moderate CYP2C19 inhibitor. Patients using this combination should be monitored closely for evidence of citalopram toxicity (eg, serotonin syndrome, QT prolongation). See separate drug interaction monographs for drugs listed as ‘Exceptions’ below. | CYP2C19 Inhibitors (Moderate) may increase the serum concentration of Citalopram. |
| CloZAPine / CYP3A4 Inhibitors (Strong) | CloZAPine | CYP3A4 Inhibitors (Strong) Interacting Members Atazanavir, Cobicistat, Darunavir, Idelalisib, Indinavir, Itraconazole, Ketoconazole (Systemic), Lonafarnib, Lopinavir, MiFEPRIStone, Nefazodone, Nelfinavir, Ombitasvir, Paritaprevir, and Ritonavir, Ombitasvir, Paritaprevir, Ritonavir, and Dasabuvir, Posaconazole, Ritonavir, Telithromycin, Tucatinib Exceptions Ceritinib, Clarithromycin, Saquinavir, Voriconazole | CYP3A4 | Arrhythmias | C: Monitor therapy | Reliability Rating Fair: Existing data/reports are inconsistent | Severity Moderate | Monitor for increased clozapine effects/toxicities, including QTc prolongation, if combined with strong CYP3A4 inhibitors. Clozapine dose reductions may be necessary. If discontinuing a strong CYP3A4 inhibitor, monitor for loss of clozapine efficacy, and consider increasing the clozapine dose if needed. See separate drug interaction monographs for drugs listed as ‘Exceptions’ below. | CYP3A4 Inhibitors (Strong) may increase the serum concentration of CloZAPine. |
| CloZAPine / CYP1A2 Inhibitors (Moderate) | CloZAPine | CYP1A2 Inhibitors (Moderate) Interacting Members Capmatinib, Deferasirox, Enoxacin, Givosiran, Methoxsalen (Systemic), Mexiletine, Rucaparib, Stiripentol, Thiabendazole, Vemurafenib Exception Ciprofloxacin (Systemic) | CYP1A2 | Arrhythmias | C: Monitor therapy | Reliability Rating Good | Severity Moderate | Monitor patients receiving moderate CYP1A2 inhibitors with clozapine for evidence of clozapine toxicity, including QTc prolongation. Clozapine dose reduction may be needed. See separate drug interaction monographs for drugs listed as ‘Exceptions’ below. | CYP1A2 Inhibitors (Moderate) may increase the serum concentration of CloZAPine. |
| CloZAPine / CYP1A2 Inhibitors (Strong) | CloZAPine | CYP1A2 Inhibitors (Strong) Interacting Members FluvoxaMINE, Viloxazine | CYP1A2 | Arrhythmias | D: Consider therapy modification | Reliability Rating Excellent | Severity Major | Reduce the dose of clozapine to one-third of the original dose when adding a strong CYP1A2 inhibitor and monitor patient response closely. Return to the original clozapine dose, as clinically appropriate, when the strong CYP1A2 inhibitor is discontinued. | CYP1A2 Inhibitors (Strong) may increase the serum concentration of CloZAPine. |
| CloZAPine / CYP3A4 Inhibitors (Moderate) | CloZAPine | CYP3A4 Inhibitors (Moderate) Interacting Members Aprepitant, Berotralstat, Conivaptan, DilTIAZem, Duvelisib, Fosamprenavir, Fosnetupitant, Grapefruit Juice, Imatinib, Isavuconazonium Sulfate, Lefamulin, Letermovir, Netupitant, Nilotinib, Schisandra Exceptions Crizotinib, Dronedarone, Erythromycin (Systemic), Fedratinib, Fluconazole, Ribociclib, Verapamil | CYP3A4 | Arrhythmias | C: Monitor therapy | Reliability Rating Fair: Existing data/reports are inconsistent | Severity Moderate | Monitor for increased clozapine effects/toxicities, including QTc prolongation, if combined with moderate CYP3A4 inhibitors. Clozapine dose reductions may be necessary. If discontinuing a moderate CYP3A4 inhibitor, monitor for loss of clozapine efficacy, and consider increasing the clozapine dose if needed. See separate drug interaction monographs for drugs listed as ‘Exceptions’ below. | CYP3A4 Inhibitors (Moderate) may increase the serum concentration of CloZAPine. |
| CloZAPine / CYP3A4 Inhibitors (Strong) | CloZAPine | CYP3A4 Inhibitors (Strong) Interacting Members Atazanavir, Cobicistat, Darunavir, Idelalisib, Indinavir, Itraconazole, Ketoconazole (Systemic), Lonafarnib, Lopinavir, MiFEPRIStone, Nefazodone, Nelfinavir, Ombitasvir, Paritaprevir, and Ritonavir, Ombitasvir, Paritaprevir, Ritonavir, and Dasabuvir, Posaconazole, Ritonavir, Telithromycin, Tucatinib Exceptions Ceritinib, Clarithromycin, Saquinavir, Voriconazole | CYP3A4 | Arrhythmias | C: Monitor therapy | Reliability Rating Fair: Existing data/reports are inconsistent | Severity Moderate | Monitor for increased clozapine effects/toxicities, including QTc prolongation, if combined with strong CYP3A4 inhibitors. Clozapine dose reductions may be necessary. If discontinuing a strong CYP3A4 inhibitor, monitor for loss of clozapine efficacy, and consider increasing the clozapine dose if needed. See separate drug interaction monographs for drugs listed as ‘Exceptions’ below. | CYP3A4 Inhibitors (Strong) may increase the serum concentration of CloZAPine. |
| Dasabuvir / CYP2C8 Inhibitors (Moderate) | Dasabuvir | CYP2C8 Inhibitors (Moderate) Interacting Members Clopidogrel, Deferasirox, Leflunomide, Selpercatinib, Teriflunomide | CYP2C8 | Arrhythmias | C: Monitor therapy | Reliability Rating Good | Severity Moderate | Monitor for increased dasabuvir concentrations and toxicities, including QTc interval prolongation, when combined with moderate CYP2C8 inhibitors. | CYP2C8 Inhibitors (Moderate) may increase the serum concentration of Dasabuvir. |
| Deutetrabenazine / CYP2D6 Inhibitors (Strong) | Deutetrabenazine | CYP2D6 Inhibitors (Strong) Interacting Members BuPROPion, Dacomitinib, FLUoxetine, PARoxetine, QuiNIDine, Quinidine (Non-Therapeutic), Tipranavir | CYP2D6 | Arrhythmias | D: Consider therapy modification | Reliability Rating Good | Severity Moderate | The total daily dose of deutetrabenazine should not exceed 36 mg, and the maximum single dose of deutetrabenazine should not exceed 18 mg, with concurrent use of a strong CYP2D6 inhibitor. Monitor patient response closely as the risk for adverse effects, including QT prolongation, may be increased with use of this combination. | CYP2D6 Inhibitors (Strong) may increase serum concentrations of the active metabolite(s) of Deutetrabenazine. |
| Disopyramide / CYP3A4 Inhibitors (Strong) | Disopyramide | CYP3A4 Inhibitors (Strong) Interacting Members Atazanavir, Cobicistat, Darunavir, Idelalisib, Indinavir, Lonafarnib, Lopinavir, MiFEPRIStone, Nefazodone, Nelfinavir, Ombitasvir, Paritaprevir, and Ritonavir, Ombitasvir, Paritaprevir, Ritonavir, and Dasabuvir, Posaconazole, Ritonavir, Telithromycin, Tucatinib Exceptions Ceritinib, Clarithromycin, Itraconazole, Ketoconazole (Systemic), Saquinavir, Voriconazole | CYP3A4 | Arrhythmias | C: Monitor therapy | Reliability Rating Good | Severity Moderate | Monitor for increased disopyramide serum concentrations and toxicities, including QTc interval prolongation, when combined with strong CYP3A4 inhibitors. See separate drug interaction monographs for drugs listed as ‘Exceptions’ below. | CYP3A4 Inhibitors (Strong) may increase the serum concentration of Disopyramide. |
| Domperidone / QT-prolonging Strong CYP3A4 Inhibitors (Moderate Risk) | Domperidone | QT-prolonging Strong CYP3A4 Inhibitors (Moderate Risk) Interacting Members Ceritinib, Clarithromycin, Saquinavir, Voriconazole | QT-prolonging Strong CYP3A4 | Arrhythmias | X: Avoid combination | Reliability Rating Good | Severity Major | Avoid concomitant use of QT-prolonging Strong CYP3A4 Inhibitors (Moderate Risk) and Domperidone. | QT-prolonging Strong CYP3A4 Inhibitors (Moderate Risk) may enhance the QTc-prolonging effect of Domperidone. QT-prolonging Strong CYP3A4 Inhibitors (Moderate Risk) may increase the serum concentration of Domperidone. |
| Erythromycin (Systemic) / CYP3A4 Inhibitors (Moderate) | Erythromycin (Systemic) | CYP3A4 Inhibitors (Moderate) Interacting Members Aprepitant, Berotralstat, Conivaptan, Duvelisib, Erythromycin (Systemic), Fedratinib, Fosamprenavir, Fosnetupitant, Grapefruit Juice, Imatinib, Isavuconazonium Sulfate, Lefamulin, Letermovir, Netupitant, Schisandra Exceptions Crizotinib, DilTIAZem, Dronedarone, Fluconazole, Nilotinib, Ribociclib, Verapamil | CYP3A4 | Arrhythmias | C: Monitor therapy | Reliability Rating Good | Severity Moderate | Monitor for increased erythromycin effects and toxicities, including QTc interval prolongation, when combined with moderate CYP3A4 inhibitors. See separate drug interaction monographs for drugs listed as ‘Exceptions’ below. | CYP3A4 Inhibitors (Moderate) may increase the serum concentration of Erythromycin (Systemic). |
| Erythromycin (Systemic) / CYP3A4 Inhibitors (Strong) | Erythromycin (Systemic) | CYP3A4 Inhibitors (Strong) Interacting Members Atazanavir, Cobicistat, Darunavir, Idelalisib, Indinavir, Itraconazole, Ketoconazole (Systemic), Lonafarnib, Lopinavir, MiFEPRIStone, Nefazodone, Nelfinavir, Ombitasvir, Paritaprevir, and Ritonavir, Ombitasvir, Paritaprevir, Ritonavir, and Dasabuvir, Posaconazole, Ritonavir, Telithromycin, Tucatinib Exceptions Ceritinib, Clarithromycin, Saquinavir, Voriconazole | CYP3A4 | Arrhythmias | C: Monitor therapy | Reliability Rating Good | Severity Moderate | Monitor for increased erythromycin effects and toxicities, including QTc interval prolongation, when combined with strong CYP3A4 inhibitors. Consider alternatives when possible. See separate drug interaction monographs for drugs listed as ‘Exceptions’ below. | CYP3A4 Inhibitors (Strong) may increase the serum concentration of Erythromycin (Systemic). |
| Escitalopram / CYP2C19 Inhibitors (Moderate) | Escitalopram | CYP2C19 Inhibitors (Moderate) Interacting Members Cannabidiol, Cenobamate, Fedratinib, Stiripentol Exceptions FLUoxetine, FluvoxaMINE, Moclobemide, Voriconazole | CYP2C19 | Arrhythmias | C: Monitor therapy | Reliability Rating Good | Severity Moderate | Monitor for increased escitalopram toxicity (including increased QTc prolongation) if combined with moderate CYP2C19 inhibitors. Consider limiting the escitalopram dose to 10 mg daily when these agents are combined. See separate drug interaction monographs for drugs listed as ‘Exceptions’ below. | CYP2C19 Inhibitors (Moderate) may increase the serum concentration of Escitalopram. |
| Flecainide / CYP2D6 Inhibitors (Strong) | Flecainide | CYP2D6 Inhibitors (Strong) Interacting Members BuPROPion, Dacomitinib, FLUoxetine, PARoxetine, Quinidine (Non-Therapeutic), Tipranavir Exception QuiNIDine | CYP2D6 | Arrhythmias | C: Monitor therapy | Reliability Rating Good | Severity Moderate | Monitor for increased flecainide effects and toxicities, including QTc interval prolongation, when combined with strong CYP2D6 inhibitors. See separate drug interaction monographs for drugs listed as ‘Exceptions’ below. | CYP2D6 Inhibitors (Strong) may increase the serum concentration of Flecainide. |
| Glasdegib / CYP3A4 Inhibitors (Strong) | Glasdegib | CYP3A4 Inhibitors (Strong) Interacting Members Atazanavir, Ceritinib, Clarithromycin, Cobicistat, Darunavir, Idelalisib, Indinavir, Itraconazole, Ketoconazole (Systemic), Lonafarnib, Lopinavir, MiFEPRIStone, Nefazodone, Nelfinavir, Ombitasvir, Paritaprevir, and Ritonavir, Ombitasvir, Paritaprevir, Ritonavir, and Dasabuvir, Posaconazole, Ritonavir, Saquinavir, Telithromycin, Tucatinib, Voriconazole | CYP3A4 | Arrhythmias | D: Consider therapy modification | Reliability Rating Good | Severity Major | Consider alternatives to this combination when possible. If the combination must be used, monitor closely for evidence of QT interval prolongation and other adverse reactions to glasdegib. | CYP3A4 Inhibitors (Strong) may increase the serum concentration of Glasdegib. |
| Ivosidenib / CYP3A4 Inhibitors (Moderate) | Ivosidenib | CYP3A4 Inhibitors (Moderate) Interacting Members Aprepitant, Berotralstat, Conivaptan, DilTIAZem, Duvelisib, Fedratinib, Fosamprenavir, Fosnetupitant, Grapefruit Juice, Imatinib, Isavuconazonium Sulfate, Lefamulin, Letermovir, Netupitant, Schisandra, Verapamil Exceptions Crizotinib, Dronedarone, Erythromycin (Systemic), Fluconazole, Nilotinib, Ribociclib | CYP3A4 | Arrhythmias | D: Consider therapy modification | Reliability Rating Good | Severity Major | Avoid use of moderate CYP3A4 inhibitors with ivosidenib and consider alternatives that are not strong or moderate inhibitors of CYP3A4 whenever possible. When combined use is not avoidable, monitor patients for increased ivosidenib toxicities, including QTc interval prolongation. See separate drug interaction monographs for drugs listed as ‘Exceptions’ below. | CYP3A4 Inhibitors (Moderate) may increase the serum concentration of Ivosidenib. |
| Ivosidenib / CYP3A4 Inhibitors (Strong) | Ivosidenib | CYP3A4 Inhibitors (Strong) Interacting Members Atazanavir, Cobicistat, Darunavir, Idelalisib, Indinavir, Itraconazole, Ketoconazole (Systemic), Lonafarnib, Lopinavir, MiFEPRIStone, Nefazodone, Nelfinavir, Ombitasvir, Paritaprevir, and Ritonavir, Ombitasvir, Paritaprevir, Ritonavir, and Dasabuvir, Posaconazole, Ritonavir, Telithromycin, Tucatinib Exceptions Ceritinib, Clarithromycin, Saquinavir, Voriconazole | CYP3A4 | Arrhythmias | D: Consider therapy modification | Reliability Rating Good | Severity Major | Avoid use of a strong CYP3A4 inhibitor with ivosidenib and consider alternatives that are not strong or moderate inhibitors of CYP3A4 whenever possible. When combined use is not avoidable, reduce the ivosidenib dose to 250 mg once daily. Monitor patients for increased ivosidenib toxicities, including QTc interval prolongation. Following discontinuation of a strong CYP3A4 inhibitor, allow a period of at least 5 half-lives of the discontinued drug to elapse before resuming the standard recommended ivosidenib dose of 500 mg once daily.  See separate drug interaction monographs for drugs listed as ‘Exceptions’ below. | CYP3A4 Inhibitors (Strong) may increase the serum concentration of Ivosidenib. |
| Mequitazine / CYP2D6 Inhibitors (Strong) | Mequitazine | CYP2D6 Inhibitors (Strong) Interacting Members BuPROPion, Dacomitinib, FLUoxetine, PARoxetine, QuiNIDine, Quinidine (Non-Therapeutic), Tipranavir | CYP2D6 | Arrhythmias | X: Avoid combination | Reliability Rating Fair: Reported in the prescribing information | Severity Moderate | Concurrent use of mequitazine with a strong CYP2D6 inhibitor is not recommended. Such use could increase mequitazine concentrations and the risk for significant adverse effects, including QT interval prolongation. | CYP2D6 Inhibitors (Strong) may increase the serum concentration of Mequitazine. |
| Ombitasvir, Paritaprevir, Ritonavir, and Dasabuvir / CYP2C8 Inhibitors (Moderate) | Ombitasvir, Paritaprevir, Ritonavir, and Dasabuvir | CYP2C8 Inhibitors (Moderate) Interacting Members Clopidogrel, Deferasirox, Leflunomide, Selpercatinib, Teriflunomide | CYP2C8 | Arrhythmias | C: Monitor therapy | Reliability Rating Good | Severity Moderate | Monitor for increased dasabuvir concentrations and toxicities, including QTc interval prolongation, when combined with moderate CYP2C8 inhibitors. | CYP2C8 Inhibitors (Moderate) may increase the serum concentration of Ombitasvir, Paritaprevir, Ritonavir, and Dasabuvir. Specifically, the concentrations of the dasabuvir component may be increased. |
| QT-prolonging CYP3A4 Substrates / Lefamulin | Lefamulin | QT-prolonging CYP3A4 Substrates Interacting Members Amiodarone, Astemizole, Bedaquiline, Ceritinib, Cisapride, Clarithromycin, Crizotinib, Dasatinib, Delamanid, Disopyramide, Dofetilide, Domperidone, Dronedarone, Encorafenib, Entrectinib, Gilteritinib, Halofantrine, Haloperidol, Ivosidenib, Methadone, Midostaurin, Nilotinib, Pimozide, QUEtiapine, QuiNIDine, QuiNINE, Ribociclib, Terfenadine | QT-prolonging CYP3A4 | Arrhythmias | X: Avoid combination | Reliability Rating Good | Severity Major | Do not use lefamulin tablets with QT-prolonging CYP3A4 substrates. Lefamulin prescribing information lists this combination as contraindicated. | Lefamulin may enhance the QTc-prolonging effect of QT-prolonging CYP3A4 Substrates. |
| QT-prolonging CYP3A4 Substrates / Posaconazole | Posaconazole | QT-prolonging CYP3A4 Substrates Interacting Members Amiodarone, Astemizole, Bedaquiline, Ceritinib, Cisapride, Clarithromycin, Crizotinib, Dasatinib, Delamanid, Disopyramide, Dofetilide, Domperidone, Dronedarone, Encorafenib, Entrectinib, Gilteritinib, Halofantrine, Haloperidol, Ivosidenib, Methadone, Midostaurin, Nilotinib, Pimozide, QUEtiapine, QuiNIDine, QuiNINE, Ribociclib, Terfenadine | QT-prolonging CYP3A4 | Arrhythmias | X: Avoid combination | Reliability Rating Fair: Reported in the prescribing information | Severity Major | Do not use posaconazole with CYP3A4 substrates that prolong the QT interval. Posaconazole labeling lists this combination as contraindicated. | Posaconazole may increase the serum concentration of QT-prolonging CYP3A4 Substrates. Such increases may lead to a greater risk for proarrhythmic effects and other similar toxicities. |
| QT-prolonging Strong CYP3A4 Inhibitors (Moderate Risk) / Entrectinib | Entrectinib | QT-prolonging Strong CYP3A4 Inhibitors (Moderate Risk) Interacting Members Ceritinib, Clarithromycin, Saquinavir, Voriconazole | QT-prolonging Strong CYP3A4 | Arrhythmias | X: Avoid combination | Reliability Rating Good | Severity Major | Avoid use of this combination. Entrectinib labeling recommends avoiding use with drugs that have a high risk of prolonging the QT interval. Further, labeling recommends avoiding use with strong CYP3A4 inhibitors. Although alternative dosing is provided if use with a strong CYP3A4 inhibitor is required, no alternatives are given for use with QT-prolonging drugs. | Entrectinib may enhance the QTc-prolonging effect of QT-prolonging Strong CYP3A4 Inhibitors (Moderate Risk). QT-prolonging Strong CYP3A4 Inhibitors (Moderate Risk) may increase the serum concentration of Entrectinib. |
| QUEtiapine / CYP3A4 Inhibitors (Moderate) | QUEtiapine | CYP3A4 Inhibitors (Moderate) Interacting Members Aprepitant, Berotralstat, Conivaptan, DilTIAZem, Duvelisib, Fedratinib, Fosamprenavir, Fosnetupitant, Grapefruit Juice, Imatinib, Isavuconazonium Sulfate, Lefamulin, Letermovir, Netupitant, Schisandra, Verapamil Exceptions Crizotinib, Dronedarone, Erythromycin (Systemic), Fluconazole, Nilotinib, Ribociclib | CYP3A4 | Arrhythmias | C: Monitor therapy | Reliability Rating Good | Severity Moderate | Monitor for increased quetiapine effects and toxicities, including QTc prolongation, when combined with moderate CYP3A4 inhibitors. See separate drug interaction monographs for drugs listed as ‘Exceptions’ below. | CYP3A4 Inhibitors (Moderate) may increase the serum concentration of QUEtiapine. |
| Quinidine (Non-Therapeutic) / CYP3A4 Inhibitors (Moderate) | Quinidine (Non-Therapeutic) | CYP3A4 Inhibitors (Moderate) Interacting Members Aprepitant, Berotralstat, Conivaptan, Crizotinib, DilTIAZem, Dronedarone, Duvelisib, Erythromycin (Systemic), Fedratinib, Fluconazole, Fosamprenavir, Fosnetupitant, Grapefruit Juice, Imatinib, Isavuconazonium Sulfate, Lefamulin, Letermovir, Netupitant, Nilotinib, Ribociclib, Schisandra, Verapamil | CYP3A4 | Arrhythmias | C: Monitor therapy | Reliability Rating Good | Severity Moderate | Monitor patients for prolonged QTc interval and other quinidine toxicities when combined with moderate CYP3A4 inhibitors. | CYP3A4 Inhibitors (Moderate) may increase the serum concentration of Quinidine (Non-Therapeutic). |
| Quinidine (Non-Therapeutic) / CYP3A4 Inhibitors (Strong) | Quinidine (Non-Therapeutic) | CYP3A4 Inhibitors (Strong) Interacting Members Atazanavir, Ceritinib, Clarithromycin, Cobicistat, Darunavir, Idelalisib, Indinavir, Itraconazole, Ketoconazole (Systemic), Lonafarnib, Lopinavir, MiFEPRIStone, Nefazodone, Nelfinavir, Ombitasvir, Paritaprevir, and Ritonavir, Ombitasvir, Paritaprevir, Ritonavir, and Dasabuvir, Posaconazole, Ritonavir, Saquinavir, Telithromycin, Tucatinib, Voriconazole | CYP3A4 | Arrhythmias | C: Monitor therapy | Reliability Rating Good | Severity Moderate | Monitor patients for prolonged QTc interval and other quinidine toxicities when combined with strong CYP3A4 inhibitors. | CYP3A4 Inhibitors (Strong) may increase the serum concentration of Quinidine (Non-Therapeutic). |
| QuiNINE / CYP3A4 Inhibitors (Strong) | QuiNINE | CYP3A4 Inhibitors (Strong) Interacting Members Atazanavir, Cobicistat, Darunavir, Idelalisib, Indinavir, Itraconazole, Ketoconazole (Systemic), Lonafarnib, MiFEPRIStone, Nefazodone, Nelfinavir, Telithromycin, Tucatinib, Voriconazole Exceptions Ceritinib, Clarithromycin, Lopinavir, Ombitasvir, Paritaprevir, and Ritonavir, Ombitasvir, Paritaprevir, Ritonavir, and Dasabuvir, Posaconazole, Ritonavir, Saquinavir | CYP3A4 | Arrhythmias | C: Monitor therapy | Reliability Rating Excellent | Severity Moderate | Monitor for increased quinine toxicities, including QTc interval prolongation, when combined with strong CYP3A4 inhibitors. See separate drug interaction monographs for drugs listed as ‘Exceptions’ below. | CYP3A4 Inhibitors (Strong) may increase the serum concentration of QuiNINE. |
| Ribociclib / CYP3A4 Inhibitors (Moderate) | Ribociclib | CYP3A4 Inhibitors (Moderate) Interacting Members Aprepitant, Berotralstat, Conivaptan, DilTIAZem, Duvelisib, Fedratinib, Fosamprenavir, Fosnetupitant, Imatinib, Isavuconazonium Sulfate, Lefamulin, Letermovir, Netupitant, Schisandra, Verapamil Exceptions Crizotinib, Dronedarone, Erythromycin (Systemic), Fluconazole, Grapefruit Juice, Nilotinib, Ribociclib | CYP3A4 | Arrhythmias | C: Monitor therapy | Reliability Rating Good | Severity Moderate | Monitor for increased ribociclib toxicities, including QTc prolongation, when combined with moderate CYP3A4 inhibitors. See separate drug interaction monographs for drugs listed as ‘Exceptions’ below. | CYP3A4 Inhibitors (Moderate) may increase the serum concentration of Ribociclib. |
| Ribociclib / CYP3A4 Inducers (Strong) | Ribociclib | CYP3A4 Inducers (Strong) Interacting Members Apalutamide, CarBAMazepine, Enzalutamide, Fosphenytoin, Lumacaftor and Ivacaftor, Mitotane, PHENobarbital, Phenytoin, Primidone, RifAMPin | CYP3A4 | Arrhythmias | X: Avoid combination | Reliability Rating Good | Severity Major | Avoid coadministration of ribociclib with strong CYP3A4 inducers when possible, and consider using an alternative with less of a potential to induce CYP3A4. | CYP3A4 Inducers (Strong) may decrease the serum concentration of Ribociclib. |
| Terfenadine / QT-prolonging Strong CYP3A4 Inhibitors (Moderate Risk) | Terfenadine | QT-prolonging Strong CYP3A4 Inhibitors (Moderate Risk) Interacting Members Ceritinib, Clarithromycin, Saquinavir, Voriconazole | QT-prolonging Strong CYP3A4 | Arrhythmias | X: Avoid combination | Reliability Rating Good | Severity Major | Avoid concomitant use of terfenadine and strong CYP3A4 inhibitors that prolong the QTc interval. | QT-prolonging Strong CYP3A4 Inhibitors (Moderate Risk) may enhance the QTc-prolonging effect of Terfenadine. QT-prolonging Strong CYP3A4 Inhibitors (Moderate Risk) may increase the serum concentration of Terfenadine. |
| Toremifene / CYP3A4 Inhibitors (Strong) | Toremifene | CYP3A4 Inhibitors (Strong) Interacting Members Atazanavir, Cobicistat, Darunavir, Idelalisib, Indinavir, Itraconazole, Ketoconazole (Systemic), Lonafarnib, Lopinavir, MiFEPRIStone, Nefazodone, Nelfinavir, Ombitasvir, Paritaprevir, and Ritonavir, Ombitasvir, Paritaprevir, Ritonavir, and Dasabuvir, Posaconazole, Ritonavir, Telithromycin, Tucatinib Exceptions Ceritinib, Clarithromycin, Saquinavir, Voriconazole | CYP3A4 | Arrhythmias | D: Consider therapy modification | Reliability Rating Good | Severity Major | Concurrent use of toremifene with strong CYP3A4 inhibitors should be avoided if possible. Interupt toremifene treatment during therapy with a strong CYP3A4 inhibitor. If coadministration is necessary, monitor patients for increased toremifene toxicities, including QTc interval prolongation. See separate drug interaction monographs for drugs listed as ‘Exceptions’ below. | CYP3A4 Inhibitors (Strong) may increase the serum concentration of Toremifene. |
| TraZODone / CYP3A4 Inhibitors (Strong) | TraZODone | CYP3A4 Inhibitors (Strong) Interacting Members Atazanavir, Ceritinib, Clarithromycin, Cobicistat, Darunavir, Idelalisib, Indinavir, Itraconazole, Ketoconazole (Systemic), Lonafarnib, Lopinavir, MiFEPRIStone, Nelfinavir, Ombitasvir, Paritaprevir, and Ritonavir, Ombitasvir, Paritaprevir, Ritonavir, and Dasabuvir, Posaconazole, Ritonavir, Saquinavir, Telithromycin, Tucatinib, Voriconazole Exception Nefazodone | CYP3A4 | Arrhythmias | D: Consider therapy modification | Reliability Rating Good | Severity Moderate | Consider the use of a lower trazodone dose and monitor for increased trazodone effects (eg, sedation, QTc prolongation) if combined with strong CYP3A4 inhibitors. See separate drug interaction monographs for drugs listed as ‘Exceptions’ below. | CYP3A4 Inhibitors (Strong) may increase the serum concentration of TraZODone. |
| Valbenazine / CYP2D6 Inhibitors (Strong) | Valbenazine | CYP2D6 Inhibitors (Strong) Interacting Members BuPROPion, Dacomitinib, FLUoxetine, PARoxetine, QuiNIDine, Quinidine (Non-Therapeutic), Tipranavir | CYP2D6 | Arrhythmias | D: Consider therapy modification | Reliability Rating Good | Severity Moderate | Reduce the valbenazine dose to 40 mg once daily when valbenazine is combined with a strong CYP2D6 inhibitor. Monitor for increased valbenazine effects/toxicities (eg, QT prolongation, somnolence, restlessness, arthralgia, vomiting) with any use of this combination. | CYP2D6 Inhibitors (Strong) may increase serum concentrations of the active metabolite(s) of Valbenazine. |
| Clopidogrel / CYP2C19 Inducers (Strong) | Clopidogrel | CYP2C19 Inducers (Strong) Interacting Members Apalutamide, RifAMPin | CYP2C19 | Bleeding | D: Consider therapy modification | Reliability Rating Good | Severity Moderate | Consider alternatives to this combination when possible. If combined, monitor for increased clopidogrel effects and toxicities (eg, bleeding) if clopidogrel is combined with a strong CYP2C19 inducer. | CYP2C19 Inducers (Strong) may increase serum concentrations of the active metabolite(s) of Clopidogrel. |
| Diclofenac (Systemic) / CYP2C9 Inhibitors (Moderate) | Diclofenac (Systemic) | CYP2C9 Inhibitors (Moderate) Interacting Members Fluconazole, MiFEPRIStone, 尼替西农 | CYP2C9 | Bleeding | C: Monitor therapy | Reliability Rating Good | Severity Moderate | Monitor for increased diclofenac toxicities (eg, gastrointestinal bleeding, renal toxicity) if combined with moderate CYP2C9 inhibitors. A dosage adjustment of diclofenac may be warranted. The prescribing information for the diclofenac and misoprostol combination product recommends a maximum dose of 50 mg twice daily when coadministered with CYP2C9 inhibitors. | CYP2C9 Inhibitors (Moderate) may increase the serum concentration of Diclofenac (Systemic). |
| Flurbiprofen (Systemic) / CYP2C9 Inhibitors (Moderate) | Flurbiprofen (Systemic) | CYP2C9 Inhibitors (Moderate) Interacting Members Fluconazole, MiFEPRIStone, 尼替西农 | CYP2C9 | Bleeding | C: Monitor therapy | Reliability Rating Excellent | Severity Moderate | Monitor for increased flurbiprofen toxicities (eg, gastrointestinal bleeding, renal toxicity) if combined with moderate CYP2C9 inhibitors. | CYP2C9 Inhibitors (Moderate) may increase the serum concentration of Flurbiprofen (Systemic). |
| Rivaroxaban / Inhibitors of CYP3A4 (Strong) and P-glycoprotein | Rivaroxaban | Inhibitors of CYP3A4 (Strong) and P-glycoprotein Interacting Members Cobicistat, Itraconazole, Ketoconazole (Systemic), Ombitasvir, Paritaprevir, and Ritonavir, Ritonavir, Tucatinib Exception Clarithromycin | CYP3A4 | Bleeding | X: Avoid combination | Reliability Rating Good | Severity Major | Avoid rivaroxaban use in combination with drugs that strongly inhibit CYP3A4 and also inhibit P-glycoprotein. Some non-US labels list this combination as a contraindication. Where data suggest that the change in rivaroxaban exposure is unlikely to increase bleeding risk with such an inhibitor (eg, clarithromycin), concurrent use may be considered. See separate drug interaction monographs for drugs listed as ‘Exceptions’ below. | Inhibitors of CYP3A4 (Strong) and P-glycoprotein may increase the serum concentration of Rivaroxaban. |
| Selective Serotonin Reuptake Inhibitors (Strong CYP2D6 Inhibitors) / DULoxetine | DULoxetine | Selective Serotonin Reuptake Inhibitors (Strong CYP2D6 Inhibitors) Interacting Members FLUoxetine, PARoxetine | CYP2D6 | Bleeding | C: Monitor therapy | Reliability Rating Good | Severity Major | Monitor closely for increased duloxetine effects/toxicities and signs and symptoms of serotonin syndrome/serotonin toxicity (eg, hyperreflexia, clonus, hyperthermia, diaphoresis, tremor, autonomic instability, mental status changes) when these drugs are combined. Consider alternatives in patients with other risk factors (eg, higher drug concentrations/doses, greater numbers of serotonergic agents) who are likely at an even greater risk for these potentially life-threatening toxicities. In addition, monitor for signs and symptoms of bleeding. | DULoxetine may enhance the antiplatelet effect of Selective Serotonin Reuptake Inhibitors (Strong CYP2D6 Inhibitors). DULoxetine may enhance the serotonergic effect of Selective Serotonin Reuptake Inhibitors (Strong CYP2D6 Inhibitors). This could result in serotonin syndrome. Selective Serotonin Reuptake Inhibitors (Strong CYP2D6 Inhibitors) may increase the serum concentration of DULoxetine. |
| Ticagrelor / CYP3A4 Inhibitors (Moderate) | Ticagrelor | CYP3A4 Inhibitors (Moderate) Interacting Members Aprepitant, Berotralstat, Conivaptan, Crizotinib, DilTIAZem, Dronedarone, Duvelisib, Erythromycin (Systemic), Fedratinib, Fluconazole, Fosamprenavir, Fosnetupitant, Grapefruit Juice, Imatinib, Isavuconazonium Sulfate, Lefamulin, Letermovir, Netupitant, Nilotinib, Ribociclib, Schisandra, Verapamil | CYP3A4 | Bleeding | C: Monitor therapy | Reliability Rating Good | Severity Moderate | Monitor for increased ticagrelor effects/toxicities (eg, bleeding) if combined with moderate CYP3A4 inhibitors. No dose adjustment of ticagrelor is recommended. | CYP3A4 Inhibitors (Moderate) may decrease serum concentrations of the active metabolite(s) of Ticagrelor. CYP3A4 Inhibitors (Moderate) may increase the serum concentration of Ticagrelor. |
| Ticagrelor / CYP3A4 Inhibitors (Strong) | Ticagrelor | CYP3A4 Inhibitors (Strong) Interacting Members Atazanavir, Ceritinib, Clarithromycin, Cobicistat, Darunavir, Idelalisib, Indinavir, Itraconazole, Ketoconazole (Systemic), Lonafarnib, Lopinavir, MiFEPRIStone, Nefazodone, Nelfinavir, Ombitasvir, Paritaprevir, and Ritonavir, Ombitasvir, Paritaprevir, Ritonavir, and Dasabuvir, Posaconazole, Ritonavir, Saquinavir, Telithromycin, Tucatinib, Voriconazole | CYP3A4 | Bleeding | X: Avoid combination | Reliability Rating Good | Severity Moderate | Avoid concomitant use of ticagrelor and strong CYP3A4 inhibitors. Ticagrelor product labeling outside of the US lists this combination as contraindicated. Itraconazole US labeling also lists use with ticagrelor as contraindicated. | CYP3A4 Inhibitors (Strong) may decrease serum concentrations of the active metabolite(s) of Ticagrelor. CYP3A4 Inhibitors (Strong) may increase the serum concentration of Ticagrelor. |
| Treprostinil / CYP2C8 Inhibitors (Strong) | Treprostinil | CYP2C8 Inhibitors (Strong) Interacting Members Gemfibrozil | CYP2C8 | Bleeding | D: Consider therapy modification | Reliability Rating Good | Severity Moderate | Reduce the initial dose of treprostinil extended release tablets to 0.125 mg twice daily, titrating by increments of 0.125 mg every 3 to 4 days, in patients receiving strong CYP2C8 inhibitors. No preemptive dose adjustment is recommended for patients receiving strong CYP2C8 inhibitors with other treprostinil formulations. If combined, monitor patients for increases in treprostinil adverse reactions (eg, hypotension, bleeding, cough, headache) and need for treprostinil dose reduction. | CYP2C8 Inhibitors (Strong) may increase the serum concentration of Treprostinil. |
| Vortioxetine / Selective Serotonin Reuptake Inhibitors (Strong CYP2D6 Inhibitors) | Vortioxetine | Selective Serotonin Reuptake Inhibitors (Strong CYP2D6 Inhibitors) Interacting Members FLUoxetine, PARoxetine | CYP2D6 | Bleeding | D: Consider therapy modification | Reliability Rating Good | Severity Major | Consider alternatives to this combination. If use is necessary, reduce the vortioxetine dose by half and monitor for signs and symptoms of serotonin syndrome/serotonin toxicity (eg, hyperreflexia, clonus, hyperthermia, diaphoresis, tremor, autonomic instability, mental status changes). Patients with other risk factors (eg, higher drug concentrations/doses, greater numbers of serotonergic agents) are likely at greater risk for these potentially life-threatening toxicities. In addition, monitor for signs and symptoms of bleeding. | Selective Serotonin Reuptake Inhibitors (Strong CYP2D6 Inhibitors) may enhance the antiplatelet effect of Vortioxetine. Selective Serotonin Reuptake Inhibitors (Strong CYP2D6 Inhibitors) may enhance the serotonergic effect of Vortioxetine. This could result in serotonin syndrome. Selective Serotonin Reuptake Inhibitors (Strong CYP2D6 Inhibitors) may increase the serum concentration of Vortioxetine. |
| Zanubrutinib / CYP3A4 Inhibitors (Strong) | Zanubrutinib | CYP3A4 Inhibitors (Strong) Interacting Members Atazanavir, Ceritinib, Clarithromycin, Cobicistat, Darunavir, Idelalisib, Indinavir, Itraconazole, Ketoconazole (Systemic), Lonafarnib, Lopinavir, MiFEPRIStone, Nefazodone, Nelfinavir, Ombitasvir, Paritaprevir, and Ritonavir, Ombitasvir, Paritaprevir, Ritonavir, and Dasabuvir, Posaconazole, Ritonavir, Saquinavir, Telithromycin, Tucatinib, Voriconazole | CYP3A4 | Bleeding | D: Consider therapy modification | Reliability Rating Good | Severity Moderate | The dose of zanubrutinib should be reduced to 80 mg once daily during coadministration with a strong CYP3A4 inhibitor. If grade 3 toxicity to zanubrutinib occurs (eg, febrile neutropenia, thrombocytopenia with bleeding, neutropenia for 10 days or longer, etc), interruption of zanubrutinib therapy should occur. See zanubrutinib prescribing information for specific guidelines on toxicity management and zanubrutinib dose interruption. If strong CYP3A4 inhibitor therapy is discontinued, the zanubrutinib dose should be increased to the recommended starting dose (160 mg twice daily or 320 mg once daily). | CYP3A4 Inhibitors (Strong) may increase the serum concentration of Zanubrutinib. |
| Drospirenone / CYP3A4 Inhibitors (Strong) | Drospirenone | CYP3A4 Inhibitors (Strong) Interacting Members Atazanavir, Ceritinib, Clarithromycin, Cobicistat, Darunavir, Idelalisib, Indinavir, Itraconazole, Ketoconazole (Systemic), Lonafarnib, Lopinavir, MiFEPRIStone, Nefazodone, Nelfinavir, Ombitasvir, Paritaprevir, and Ritonavir, Ombitasvir, Paritaprevir, Ritonavir, and Dasabuvir, Posaconazole, Ritonavir, Saquinavir, Telithromycin, Tucatinib, Voriconazole | CYP3A4 | Hyperkalemia | D: Consider therapy modification | Reliability Rating Good | Severity Major | Drospirenone use is contraindicated specifically when the strong CYP3A4 inhibitors atazanavir and cobicistat are administered concurrently, as the impact of higher drospirenone concentrations may result in hyperkalemia. Caution should be used when drospirenone is coadministered with other strong CYP3A4 inhibitors. Patients at higher risk for hyperkalemia should be monitored for evidence of hyperkalemia during their first treatment cycle exposed to this combination. Those at higher risk include, but are not limited to, those with impaired renal or hepatic function, adrenal insufficiency, or concurrent use of other medications that have potassium-retaining properties. | CYP3A4 Inhibitors (Strong) may increase the serum concentration of Drospirenone. |
| Fedratinib / CYP3A4 Inhibitors (Moderate) | Fedratinib | CYP3A4 Inhibitors (Moderate) Interacting Members Aprepitant, Berotralstat, Conivaptan, Crizotinib, DilTIAZem, Dronedarone, Duvelisib, Erythromycin (Systemic), Fedratinib, Fosamprenavir, Fosnetupitant, Grapefruit Juice, Imatinib, Isavuconazonium Sulfate, Lefamulin, Letermovir, Netupitant, Nilotinib, Ribociclib, Schisandra, Verapamil Exception Fluconazole | CYP3A4 | Anemia | C: Monitor therapy | Reliability Rating Good | Severity Minor | Monitor for increased fedratinib adverse effects (eg, anemia, thrombocytopenia) if fedratinib is coadministered with moderate CYP3A4 inhibitors. See separate drug interaction monographs for drugs listed as ‘Exceptions’ below. | CYP3A4 Inhibitors (Moderate) may increase the serum concentration of Fedratinib. |
| Brentuximab Vedotin / CYP3A4 Inhibitors (Strong) | Brentuximab Vedotin | CYP3A4 Inhibitors (Strong) Interacting Members Atazanavir, Ceritinib, Clarithromycin, Cobicistat, Darunavir, Idelalisib, Indinavir, Itraconazole, Ketoconazole (Systemic), Lonafarnib, Lopinavir, MiFEPRIStone, Nefazodone, Nelfinavir, Ombitasvir, Paritaprevir, and Ritonavir, Ombitasvir, Paritaprevir, Ritonavir, and Dasabuvir, Posaconazole, Ritonavir, Saquinavir, Telithromycin, Tucatinib, Voriconazole | CYP3A4 | Neutropenia | C: Monitor therapy | Reliability Rating Good | Severity Moderate | Increase monitoring for evidence of brentuximab vedotin toxicity (eg, neuropathy, neutropenia) in patients receiving a strong CYP3A4 inhibitor. | CYP3A4 Inhibitors (Strong) may increase the serum concentration of Brentuximab Vedotin. Specifically, concentrations of the active monomethyl auristatin E (MMAE) component may be increased. |
| Fostamatinib / CYP3A4 Inhibitors (Strong) | Fostamatinib | CYP3A4 Inhibitors (Strong) Interacting Members Atazanavir, Ceritinib, Clarithromycin, Cobicistat, Darunavir, Idelalisib, Indinavir, Itraconazole, Ketoconazole (Systemic), Lonafarnib, Lopinavir, MiFEPRIStone, Nefazodone, Nelfinavir, Ombitasvir, Paritaprevir, and Ritonavir, Ombitasvir, Paritaprevir, Ritonavir, and Dasabuvir, Posaconazole, Ritonavir, Saquinavir, Telithromycin, Tucatinib, Voriconazole | CYP3A4 | Neutropenia | C: Monitor therapy | Reliability Rating Good | Severity Moderate | Monitor patients for increased fostamatinib toxicities when combined with strong CYP3A4 inhibitors. Specifically monitor for toxicities that may require fostamatinib dose reductions (ie, hypertension, hepatoxicity, diarrhea, neutropenia) when these agents are combined. | CYP3A4 Inhibitors (Strong) may increase serum concentrations of the active metabolite(s) of Fostamatinib. |
| Irinotecan Products / CYP3A4 Inhibitors (Moderate) | Irinotecan Products | CYP3A4 Inhibitors (Moderate) Interacting Members Aprepitant, Berotralstat, Conivaptan, Crizotinib, DilTIAZem, Dronedarone, Duvelisib, Erythromycin (Systemic), Fedratinib, Fluconazole, Fosamprenavir, Fosnetupitant, Imatinib, Isavuconazonium Sulfate, Lefamulin, Letermovir, Netupitant, Nilotinib, Ribociclib, Schisandra, Verapamil Exception Grapefruit Juice | CYP3A4 | Neutropenia | C: Monitor therapy | Reliability Rating Good | Severity Moderate | Monitor for increased irinotecan toxicities (eg, neutropenia, diarrhea) if combined with moderate CYP3A4 inhibitors. | CYP3A4 Inhibitors (Moderate) may increase serum concentrations of the active metabolite(s) of Irinotecan Products. Specifically, the serum concentration of SN-38 may be increased. CYP3A4 Inhibitors (Moderate) may increase the serum concentration of Irinotecan Products. |
| Irinotecan Products / CYP3A4 Inhibitors (Strong) | Irinotecan Products | CYP3A4 Inhibitors (Strong) Interacting Members Ceritinib, Clarithromycin, Cobicistat, Darunavir, Idelalisib, Indinavir, Lonafarnib, Lopinavir, MiFEPRIStone, Nefazodone, Nelfinavir, Ombitasvir, Paritaprevir, and Ritonavir, Ombitasvir, Paritaprevir, Ritonavir, and Dasabuvir, Posaconazole, Ritonavir, Saquinavir, Telithromycin, Tucatinib, Voriconazole Exceptions Atazanavir, Itraconazole, Ketoconazole (Systemic) | CYP3A4 | Neutropenia | D: Consider therapy modification | Reliability Rating Good | Severity Major | Avoid administration of strong CYP3A4 inhibitors during and within 1 week prior to irinotecan administration, unless no therapeutic alternatives to these agents exist. If combined, monitor closely for increased irinotecan toxicities (eg, neutropenia, diarrhea). See separate drug interaction monographs for drugs listed as 'Exceptions' below. | CYP3A4 Inhibitors (Strong) may increase serum concentrations of the active metabolite(s) of Irinotecan Products. Specifically, serum concentrations of SN-38 may be increased. |
| PACLitaxel (Conventional) / CYP2C8 Inhibitors (Moderate) | PACLitaxel (Conventional) | CYP2C8 Inhibitors (Moderate) Interacting Members Clopidogrel, Deferasirox, Leflunomide, Selpercatinib, Teriflunomide | CYP2C8 | Neutropenia | C: Monitor therapy | Reliability Rating Good | Severity Major | Monitor for an increased incidence or severity of paclitaxel toxicities (eg, neuropathy, neutropenia) when combined with moderate CYP2C8 inhibitors. | CYP2C8 Inhibitors (Moderate) may increase the serum concentration of PACLitaxel (Conventional). |
| PACLitaxel (Conventional) / CYP2C8 Inhibitors (Strong) | PACLitaxel (Conventional) | CYP2C8 Inhibitors (Strong) Interacting Members Gemfibrozil | CYP2C8 | Neutropenia | C: Monitor therapy | Reliability Rating Good | Severity Major | Monitor for an increased incidence or severity of paclitaxel toxicities (eg, neuropathy, neutropenia) when combined with strong CYP2C8 inhibitors. | CYP2C8 Inhibitors (Strong) may increase the serum concentration of PACLitaxel (Conventional). |
| PACLitaxel (Protein Bound) / CYP2C8 Inhibitors (Moderate) | PACLitaxel (Protein Bound) | CYP2C8 Inhibitors (Moderate) Interacting Members Clopidogrel, Deferasirox, Leflunomide, Selpercatinib, Teriflunomide | CYP2C8 | Neutropenia | C: Monitor therapy | Reliability Rating Good | Severity Moderate | Monitor for an increased incidence or severity of paclitaxel toxicities (eg, neuropathy, neutropenia) when combined with moderate CYP2C8 inhibitors. | CYP2C8 Inhibitors (Moderate) may increase the serum concentration of PACLitaxel (Protein Bound). |
| Zanubrutinib / CYP3A4 Inhibitors (Strong) | Zanubrutinib | CYP3A4 Inhibitors (Strong) Interacting Members Atazanavir, Ceritinib, Clarithromycin, Cobicistat, Darunavir, Idelalisib, Indinavir, Itraconazole, Ketoconazole (Systemic), Lonafarnib, Lopinavir, MiFEPRIStone, Nefazodone, Nelfinavir, Ombitasvir, Paritaprevir, and Ritonavir, Ombitasvir, Paritaprevir, Ritonavir, and Dasabuvir, Posaconazole, Ritonavir, Saquinavir, Telithromycin, Tucatinib, Voriconazole | CYP3A4 | Neutropenia | D: Consider therapy modification | Reliability Rating Good | Severity Moderate | The dose of zanubrutinib should be reduced to 80 mg once daily during coadministration with a strong CYP3A4 inhibitor. If grade 3 toxicity to zanubrutinib occurs (eg, febrile neutropenia, thrombocytopenia with bleeding, neutropenia for 10 days or longer, etc), interruption of zanubrutinib therapy should occur. See zanubrutinib prescribing information for specific guidelines on toxicity management and zanubrutinib dose interruption. If strong CYP3A4 inhibitor therapy is discontinued, the zanubrutinib dose should be increased to the recommended starting dose (160 mg twice daily or 320 mg once daily). | CYP3A4 Inhibitors (Strong) may increase the serum concentration of Zanubrutinib. |
| Fedratinib / CYP3A4 Inhibitors (Moderate) | Fedratinib | CYP3A4 Inhibitors (Moderate) Interacting Members Aprepitant, Berotralstat, Conivaptan, Crizotinib, DilTIAZem, Dronedarone, Duvelisib, Erythromycin (Systemic), Fedratinib, Fosamprenavir, Fosnetupitant, Grapefruit Juice, Imatinib, Isavuconazonium Sulfate, Lefamulin, Letermovir, Netupitant, Nilotinib, Ribociclib, Schisandra, Verapamil Exception Fluconazole | CYP3A4 | Thrombocytopenia | C: Monitor therapy | Reliability Rating Good | Severity Minor | Monitor for increased fedratinib adverse effects (eg, anemia, thrombocytopenia) if fedratinib is coadministered with moderate CYP3A4 inhibitors. See separate drug interaction monographs for drugs listed as ‘Exceptions’ below. | CYP3A4 Inhibitors (Moderate) may increase the serum concentration of Fedratinib. |
| Zanubrutinib / CYP3A4 Inhibitors (Strong) | Zanubrutinib | CYP3A4 Inhibitors (Strong) Interacting Members Atazanavir, Ceritinib, Clarithromycin, Cobicistat, Darunavir, Idelalisib, Indinavir, Itraconazole, Ketoconazole (Systemic), Lonafarnib, Lopinavir, MiFEPRIStone, Nefazodone, Nelfinavir, Ombitasvir, Paritaprevir, and Ritonavir, Ombitasvir, Paritaprevir, Ritonavir, and Dasabuvir, Posaconazole, Ritonavir, Saquinavir, Telithromycin, Tucatinib, Voriconazole | CYP3A4 | Thrombocytopenia | D: Consider therapy modification | Reliability Rating Good | Severity Moderate | The dose of zanubrutinib should be reduced to 80 mg once daily during coadministration with a strong CYP3A4 inhibitor. If grade 3 toxicity to zanubrutinib occurs (eg, febrile neutropenia, thrombocytopenia with bleeding, neutropenia for 10 days or longer, etc), interruption of zanubrutinib therapy should occur. See zanubrutinib prescribing information for specific guidelines on toxicity management and zanubrutinib dose interruption. If strong CYP3A4 inhibitor therapy is discontinued, the zanubrutinib dose should be increased to the recommended starting dose (160 mg twice daily or 320 mg once daily). | CYP3A4 Inhibitors (Strong) may increase the serum concentration of Zanubrutinib. |
| AtorvaSTATin / CYP3A4 Inhibitors (Moderate) | AtorvaSTATin | CYP3A4 Inhibitors (Moderate) Interacting Members Aprepitant, Berotralstat, Conivaptan, Crizotinib, DilTIAZem, Dronedarone, Duvelisib, Erythromycin (Systemic), Fedratinib, Fluconazole, Fosnetupitant, Imatinib, Isavuconazonium Sulfate, Lefamulin, Netupitant, Nilotinib, Ribociclib, Schisandra Exceptions Fosamprenavir, Grapefruit Juice, Letermovir, Verapamil | CYP3A4 | Rhabdomyolysis | C: Monitor therapy | Reliability Rating Good | Severity Moderate | Monitor for increased atorvastatin adverse effects (eg, myopathy, rhabdomyolysis) during coadministration with moderate CYP3A4 inhibitors. See separate drug interaction monographs for drugs listed as ‘Exceptions’ below. | CYP3A4 Inhibitors (Moderate) may increase the serum concentration of AtorvaSTATin. |
| Fluvastatin / CYP2C9 Inhibitors (Moderate) | Fluvastatin | CYP2C9 Inhibitors (Moderate) Interacting Members Fluconazole, MiFEPRIStone, 尼替西农 | CYP2C9 | Rhabdomyolysis | D: Consider therapy modification | Reliability Rating Good | Severity Moderate | Fluvastatin should be used at the lowest effective dose and should not exceed 20 mg twice daily when combined with moderate CYP2C9 inhibitors. Monitor patients closely for increased fluvastatin toxicities (eg, myalgias, rhabdomyolysis) whenever these agents are combined. | CYP2C9 Inhibitors (Moderate) may increase the serum concentration of Fluvastatin. |
| Lovastatin / CYP3A4 Inhibitors (Strong) | Lovastatin | CYP3A4 Inhibitors (Strong) Interacting Members Atazanavir, Ceritinib, Clarithromycin, Cobicistat, Darunavir, Idelalisib, Indinavir, Itraconazole*, Ketoconazole (Systemic), Lonafarnib, Lopinavir, MiFEPRIStone, Nefazodone, Nelfinavir, Ombitasvir, Paritaprevir, and Ritonavir, Ombitasvir, Paritaprevir, Ritonavir, and Dasabuvir, Posaconazole, Ritonavir, Saquinavir, Telithromycin, Tucatinib, Voriconazole | CYP3A4 | Rhabdomyolysis | X: Avoid combination | Reliability Rating Excellent | Severity Major | Do not use lovastatin with strong CYP3A4 inhibitors. Lovastatin prescribing information states this combination is contraindicated. If treatment with a strong CYP3A4 inhibitor is unavoidable, discontinue lovastatin during therapy. Alternative HMG-CoA reductase inhibitors that are less likely to be significantly affected by CYP3A4 inhibition include fluvastatin, rosuvastatin, pitavastatin, and pravastatin. | CYP3A4 Inhibitors (Strong) may increase serum concentrations of the active metabolite(s) of Lovastatin. CYP3A4 Inhibitors (Strong) may increase the serum concentration of Lovastatin. |
| Simvastatin / CYP3A4 Inhibitors (Moderate) | Simvastatin | CYP3A4 Inhibitors (Moderate) Interacting Members Aprepitant, Berotralstat, Conivaptan, Crizotinib, Duvelisib, Fedratinib, Fluconazole, Fosamprenavir, Fosnetupitant, Imatinib, Isavuconazonium Sulfate, Lefamulin, Netupitant, Nilotinib, Ribociclib, Schisandra Exceptions DilTIAZem, Dronedarone, Erythromycin (Systemic), Grapefruit Juice, Letermovir, Verapamil | CYP3A4 | Rhabdomyolysis | C: Monitor therapy | Reliability Rating Excellent | Severity Moderate | Monitor for increased simvastatin adverse effects (eg, myopathy, rhabdomyolysis) if simvastatin and moderate CYP3A4 inhibitors are coadministered. Consider limiting simvastatin doses and using the lowest simvastatin dose necessary. See separate drug interaction monographs for drugs listed as ‘Exceptions’ below. | CYP3A4 Inhibitors (Moderate) may increase serum concentrations of the active metabolite(s) of Simvastatin. CYP3A4 Inhibitors (Moderate) may increase the serum concentration of Simvastatin. |
| Simvastatin / CYP3A4 Inhibitors (Strong) | Simvastatin | CYP3A4 Inhibitors (Strong) Interacting Members Ceritinib, Clarithromycin, Cobicistat, Idelalisib, Itraconazole, Ketoconazole (Systemic), Lonafarnib, Lopinavir, MiFEPRIStone, Nefazodone, Nelfinavir, Ombitasvir, Paritaprevir, and Ritonavir, Ombitasvir, Paritaprevir, Ritonavir, and Dasabuvir, Posaconazole, Ritonavir, Saquinavir, Telithromycin, Tucatinib, Voriconazole Exceptions Atazanavir, Darunavir, Indinavir | CYP3A4 | Rhabdomyolysis | X: Avoid combination | Reliability Rating Excellent | Severity Major | Do not use simvastatin with strong CYP3A4 inhibitors. Simvastatin prescribing information lists this combination as contraindicated. If treatment with a strong CYP3A4 inhibitor is required, discontinue simvastatin during therapy. Alternative HMG-CoA reductase inhibitors that are less likely to be significantly affected by CYP3A4 inhibition include fluvastatin, rosuvastatin, pitavastatin, and pravastatin. See separate drug interaction monographs for drugs listed as ‘Exceptions’ below. | CYP3A4 Inhibitors (Strong) may increase serum concentrations of the active metabolite(s) of Simvastatin. CYP3A4 Inhibitors (Strong) may increase the serum concentration of Simvastatin. |
| Simvastatin / CYP3A4 Inhibitors (Weak) | Simvastatin | CYP3A4 Inhibitors (Weak) Interacting Members ALPRAZolam, Berberine, Bicalutamide, Bitter Orange, Chlorzoxazone, Cilostazol, Cimetidine, Clotrimazole (Oral), Delavirdine, Everolimus, FluvoxaMINE, Fosaprepitant, Givosiran, Goldenseal, Grazoprevir, Idebenone, Iloperidone, Isoniazid, Istradefylline, Ivacaftor, Lapatinib, Larotrectinib, Lomitapide, Lurasidone, Osilodrostat, Palbociclib, PAZOPanib, Peppermint, Piperaquine, Propiverine, Propofol, QuiNIDine, Quinupristin and Dalfopristin, Resveratrol, Roxithromycin, Rucaparib, Selpercatinib, Tofisopam, Viloxazine, Voxelotor Exceptions Amiodarone, AmLODIPine, Ciprofloxacin (Systemic), CycloSPORINE (Systemic), Danazol, Glecaprevir and Pibrentasvir, Levamlodipine, Ranolazine | CYP3A4 | Rhabdomyolysis | C: Monitor therapy | Reliability Rating Good | Severity Moderate | Monitor for increased simvastatin adverse effects (eg, LFT elevation, myopathy, rhabdomyolysis) if simvastatin and weak CYP3A4 inhibitors are coadministered. See separate drug interaction monographs for drugs listed as ‘Exceptions’ below. | CYP3A4 Inhibitors (Weak) may increase serum concentrations of the active metabolite(s) of Simvastatin. CYP3A4 Inhibitors (Weak) may increase the serum concentration of Simvastatin. |
| Erlotinib / CYP3A4 Inhibitors (Strong) | Erlotinib | CYP3A4 Inhibitors (Strong) Interacting Members Atazanavir, Ceritinib, Clarithromycin, Cobicistat, Darunavir, Idelalisib, Indinavir, Itraconazole, Ketoconazole (Systemic), Lonafarnib, Lopinavir, MiFEPRIStone, Nefazodone, Nelfinavir, Ombitasvir, Paritaprevir, and Ritonavir, Ombitasvir, Paritaprevir, Ritonavir, and Dasabuvir, Posaconazole, Ritonavir, Saquinavir, Telithromycin, Tucatinib, Voriconazole | CYP3A4 | Severe skin reactions | D: Consider therapy modification | Reliability Rating Good | Severity Moderate | Avoid use of this combination when possible. When the combination must be used, monitor the patient closely for the development of severe erlotinib-associated adverse reactions (eg, severe diarrhea, severe skin reactions). If severe adverse reactions do occur, reduce the erlotinib dose (in 50 mg decrements). | CYP3A4 Inhibitors (Strong) may increase the serum concentration of Erlotinib. |
| Erlotinib / CYP3A4 Inhibitors (Strong) | Erlotinib | CYP3A4 Inhibitors (Strong) Interacting Members Atazanavir, Ceritinib, Clarithromycin, Cobicistat, Darunavir, Idelalisib, Indinavir, Itraconazole, Ketoconazole (Systemic), Lonafarnib, Lopinavir, MiFEPRIStone, Nefazodone, Nelfinavir, Ombitasvir, Paritaprevir, and Ritonavir, Ombitasvir, Paritaprevir, Ritonavir, and Dasabuvir, Posaconazole, Ritonavir, Saquinavir, Telithromycin, Tucatinib, Voriconazole | CYP3A4 | Diarrhea | D: Consider therapy modification | Reliability Rating Good | Severity Moderate | Avoid use of this combination when possible. When the combination must be used, monitor the patient closely for the development of severe erlotinib-associated adverse reactions (eg, severe diarrhea, severe skin reactions). If severe adverse reactions do occur, reduce the erlotinib dose (in 50 mg decrements). | CYP3A4 Inhibitors (Strong) may increase the serum concentration of Erlotinib. |
| Fostamatinib / CYP3A4 Inhibitors (Strong) | Fostamatinib | CYP3A4 Inhibitors (Strong) Interacting Members Atazanavir, Ceritinib, Clarithromycin, Cobicistat, Darunavir, Idelalisib, Indinavir, Itraconazole, Ketoconazole (Systemic), Lonafarnib, Lopinavir, MiFEPRIStone, Nefazodone, Nelfinavir, Ombitasvir, Paritaprevir, and Ritonavir, Ombitasvir, Paritaprevir, Ritonavir, and Dasabuvir, Posaconazole, Ritonavir, Saquinavir, Telithromycin, Tucatinib, Voriconazole | CYP3A4 | Diarrhea | C: Monitor therapy | Reliability Rating Good | Severity Moderate | Monitor patients for increased fostamatinib toxicities when combined with strong CYP3A4 inhibitors. Specifically monitor for toxicities that may require fostamatinib dose reductions (ie, hypertension, hepatoxicity, diarrhea, neutropenia) when these agents are combined. | CYP3A4 Inhibitors (Strong) may increase serum concentrations of the active metabolite(s) of Fostamatinib. |
| Irinotecan Products / CYP3A4 Inhibitors (Moderate) | Irinotecan Products | CYP3A4 Inhibitors (Moderate) Interacting Members Aprepitant, Berotralstat, Conivaptan, Crizotinib, DilTIAZem, Dronedarone, Duvelisib, Erythromycin (Systemic), Fedratinib, Fluconazole, Fosamprenavir, Fosnetupitant, Imatinib, Isavuconazonium Sulfate, Lefamulin, Letermovir, Netupitant, Nilotinib, Ribociclib, Schisandra, Verapamil Exception Grapefruit Juice | CYP3A4 | Diarrhea | C: Monitor therapy | Reliability Rating Good | Severity Moderate | Monitor for increased irinotecan toxicities (eg, neutropenia, diarrhea) if combined with moderate CYP3A4 inhibitors. | CYP3A4 Inhibitors (Moderate) may increase serum concentrations of the active metabolite(s) of Irinotecan Products. Specifically, the serum concentration of SN-38 may be increased. CYP3A4 Inhibitors (Moderate) may increase the serum concentration of Irinotecan Products. |
| Irinotecan Products / CYP3A4 Inhibitors (Strong) | Irinotecan Products | CYP3A4 Inhibitors (Strong) Interacting Members Ceritinib, Clarithromycin, Cobicistat, Darunavir, Idelalisib, Indinavir, Lonafarnib, Lopinavir, MiFEPRIStone, Nefazodone, Nelfinavir, Ombitasvir, Paritaprevir, and Ritonavir, Ombitasvir, Paritaprevir, Ritonavir, and Dasabuvir, Posaconazole, Ritonavir, Saquinavir, Telithromycin, Tucatinib, Voriconazole Exceptions Atazanavir, Itraconazole, Ketoconazole (Systemic) | CYP3A4 | Diarrhea | D: Consider therapy modification | Reliability Rating Good | Severity Major | Avoid administration of strong CYP3A4 inhibitors during and within 1 week prior to irinotecan administration, unless no therapeutic alternatives to these agents exist. If combined, monitor closely for increased irinotecan toxicities (eg, neutropenia, diarrhea). See separate drug interaction monographs for drugs listed as 'Exceptions' below. | CYP3A4 Inhibitors (Strong) may increase serum concentrations of the active metabolite(s) of Irinotecan Products. Specifically, serum concentrations of SN-38 may be increased. |
| Naldemedine / CYP3A4 Inhibitors (Moderate) | Naldemedine | CYP3A4 Inhibitors (Moderate) Interacting Members Aprepitant, Berotralstat, Conivaptan, Crizotinib, DilTIAZem, Dronedarone, Duvelisib, Erythromycin (Systemic), Fedratinib, Fluconazole, Fosamprenavir, Fosnetupitant, Grapefruit Juice, Imatinib, Isavuconazonium Sulfate, Lefamulin, Letermovir, Netupitant, Nilotinib, Ribociclib, Schisandra, Verapamil | CYP3A4 | Diarrhea | C: Monitor therapy | Reliability Rating Good | Severity Moderate | Monitor for increased naldemedine effects (eg, abdominal pain, nausea, diarrhea, opioid withdrawal) if combined with a moderate CYP3A4 inhibitor. | CYP3A4 Inhibitors (Moderate) may increase the serum concentration of Naldemedine. |
| Naldemedine / CYP3A4 Inhibitors (Strong) | Naldemedine | CYP3A4 Inhibitors (Strong) Interacting Members Atazanavir, Ceritinib, Clarithromycin, Cobicistat, Darunavir, Idelalisib, Indinavir, Itraconazole, Ketoconazole (Systemic), Lonafarnib, Lopinavir, MiFEPRIStone, Nefazodone, Nelfinavir, Ombitasvir, Paritaprevir, and Ritonavir, Ombitasvir, Paritaprevir, Ritonavir, and Dasabuvir, Posaconazole, Ritonavir, Saquinavir, Telithromycin, Tucatinib, Voriconazole | CYP3A4 | Diarrhea | C: Monitor therapy | Reliability Rating Good | Severity Moderate | Monitor for increased naldemedine effects (eg, abdominal pain, nausea, diarrhea, opioid withdrawal) if combined with a strong CYP3A4 inhibitor. | CYP3A4 Inhibitors (Strong) may increase the serum concentration of Naldemedine. |
| Naloxegol / CYP3A4 Inhibitors (Moderate) | Naloxegol | CYP3A4 Inhibitors (Moderate) Interacting Members Aprepitant, Berotralstat, Conivaptan, Crizotinib, DilTIAZem, Dronedarone, Duvelisib, Erythromycin (Systemic), Fedratinib, Fluconazole, Fosamprenavir, Fosnetupitant, Grapefruit Juice, Imatinib, Isavuconazonium Sulfate, Lefamulin, Letermovir, Netupitant, Nilotinib, Ribociclib, Schisandra, Verapamil | CYP3A4 | Diarrhea | D: Consider therapy modification | Reliability Rating Good | Severity Moderate | The use of naloxegol and moderate CYP3A4 inhibitors should be avoided. If concurrent use is unavoidable, reduce naloxegol dose to 12.5 mg once daily and monitor for signs of toxicity, specifically signs and symptoms of opiate withdrawal syndrome (eg, hyperhidrosis, chills, diarrhea, abdominal pain, anxiety, irritability). | CYP3A4 Inhibitors (Moderate) may increase the serum concentration of Naloxegol. |
| Tucatinib / CYP2C8 Inhibitors (Moderate) | Tucatinib | CYP2C8 Inhibitors (Moderate) Interacting Members Clopidogrel, Deferasirox, Leflunomide, Selpercatinib, Teriflunomide | CYP2C8 | Diarrhea | C: Monitor therapy | Reliability Rating Fair: Reported in the prescribing information | Severity Moderate | Close clinical and laboratory monitoring for signs and symptoms of tucatinib toxicity (eg, diarrhea, liver function tests) is warranted during coadministration of tucatinib and moderate CYP2C8 inhibitors. | CYP2C8 Inhibitors (Moderate) may increase the serum concentration of Tucatinib. |
| Naldemedine / CYP3A4 Inhibitors (Moderate) | Naldemedine | CYP3A4 Inhibitors (Moderate) Interacting Members Aprepitant, Berotralstat, Conivaptan, Crizotinib, DilTIAZem, Dronedarone, Duvelisib, Erythromycin (Systemic), Fedratinib, Fluconazole, Fosamprenavir, Fosnetupitant, Grapefruit Juice, Imatinib, Isavuconazonium Sulfate, Lefamulin, Letermovir, Netupitant, Nilotinib, Ribociclib, Schisandra, Verapamil | CYP3A4 | Abdominal pain | C: Monitor therapy | Reliability Rating Good | Severity Moderate | Monitor for increased naldemedine effects (eg, abdominal pain, nausea, diarrhea, opioid withdrawal) if combined with a moderate CYP3A4 inhibitor. | CYP3A4 Inhibitors (Moderate) may increase the serum concentration of Naldemedine. |
| Naldemedine / CYP3A4 Inhibitors (Strong) | Naldemedine | CYP3A4 Inhibitors (Strong) Interacting Members Atazanavir, Ceritinib, Clarithromycin, Cobicistat, Darunavir, Idelalisib, Indinavir, Itraconazole, Ketoconazole (Systemic), Lonafarnib, Lopinavir, MiFEPRIStone, Nefazodone, Nelfinavir, Ombitasvir, Paritaprevir, and Ritonavir, Ombitasvir, Paritaprevir, Ritonavir, and Dasabuvir, Posaconazole, Ritonavir, Saquinavir, Telithromycin, Tucatinib, Voriconazole | CYP3A4 | Abdominal pain | C: Monitor therapy | Reliability Rating Good | Severity Moderate | Monitor for increased naldemedine effects (eg, abdominal pain, nausea, diarrhea, opioid withdrawal) if combined with a strong CYP3A4 inhibitor. | CYP3A4 Inhibitors (Strong) may increase the serum concentration of Naldemedine. |
| Naloxegol / CYP3A4 Inhibitors (Moderate) | Naloxegol | CYP3A4 Inhibitors (Moderate) Interacting Members Aprepitant, Berotralstat, Conivaptan, Crizotinib, DilTIAZem, Dronedarone, Duvelisib, Erythromycin (Systemic), Fedratinib, Fluconazole, Fosamprenavir, Fosnetupitant, Grapefruit Juice, Imatinib, Isavuconazonium Sulfate, Lefamulin, Letermovir, Netupitant, Nilotinib, Ribociclib, Schisandra, Verapamil | CYP3A4 | Abdominal pain | D: Consider therapy modification | Reliability Rating Good | Severity Moderate | The use of naloxegol and moderate CYP3A4 inhibitors should be avoided. If concurrent use is unavoidable, reduce naloxegol dose to 12.5 mg once daily and monitor for signs of toxicity, specifically signs and symptoms of opiate withdrawal syndrome (eg, hyperhidrosis, chills, diarrhea, abdominal pain, anxiety, irritability). | CYP3A4 Inhibitors (Moderate) may increase the serum concentration of Naloxegol. |
| Naldemedine / CYP3A4 Inhibitors (Moderate) | Naldemedine | CYP3A4 Inhibitors (Moderate) Interacting Members Aprepitant, Berotralstat, Conivaptan, Crizotinib, DilTIAZem, Dronedarone, Duvelisib, Erythromycin (Systemic), Fedratinib, Fluconazole, Fosamprenavir, Fosnetupitant, Grapefruit Juice, Imatinib, Isavuconazonium Sulfate, Lefamulin, Letermovir, Netupitant, Nilotinib, Ribociclib, Schisandra, Verapamil | CYP3A4 | Nausea | C: Monitor therapy | Reliability Rating Good | Severity Moderate | Monitor for increased naldemedine effects (eg, abdominal pain, nausea, diarrhea, opioid withdrawal) if combined with a moderate CYP3A4 inhibitor. | CYP3A4 Inhibitors (Moderate) may increase the serum concentration of Naldemedine. |
| Naldemedine / CYP3A4 Inhibitors (Strong) | Naldemedine | CYP3A4 Inhibitors (Strong) Interacting Members Atazanavir, Ceritinib, Clarithromycin, Cobicistat, Darunavir, Idelalisib, Indinavir, Itraconazole, Ketoconazole (Systemic), Lonafarnib, Lopinavir, MiFEPRIStone, Nefazodone, Nelfinavir, Ombitasvir, Paritaprevir, and Ritonavir, Ombitasvir, Paritaprevir, Ritonavir, and Dasabuvir, Posaconazole, Ritonavir, Saquinavir, Telithromycin, Tucatinib, Voriconazole | CYP3A4 | Nausea | C: Monitor therapy | Reliability Rating Good | Severity Moderate | Monitor for increased naldemedine effects (eg, abdominal pain, nausea, diarrhea, opioid withdrawal) if combined with a strong CYP3A4 inhibitor. | CYP3A4 Inhibitors (Strong) may increase the serum concentration of Naldemedine. |
| Upadacitinib / CYP3A4 Inhibitors (Strong) | Upadacitinib | CYP3A4 Inhibitors (Strong) Interacting Members Atazanavir, Ceritinib, Clarithromycin, Cobicistat, Darunavir, Idelalisib, Indinavir, Itraconazole, Ketoconazole (Systemic), Lonafarnib, Lopinavir, MiFEPRIStone, Nefazodone, Nelfinavir, Ombitasvir, Paritaprevir, and Ritonavir, Ombitasvir, Paritaprevir, Ritonavir, and Dasabuvir, Posaconazole, Ritonavir, Saquinavir, Telithromycin, Tucatinib, Voriconazole | CYP3A4 | Nausea | C: Monitor therapy | Reliability Rating Good | Severity Moderate | Monitor for increased upadacitinib toxicities (eg, infection, nausea, signs and symptoms of blood clot) if combined with strong CYP3A4 inhibitors. | CYP3A4 Inhibitors (Strong) may increase the serum concentration of Upadacitinib. |
| Theophylline Derivatives / CYP1A2 Inhibitors (Moderate) | TheopAcebrophylline, Aminophylline, Dyphylline, Theophyllinehylline Derivatives | CYP1A2 Inhibitors (Moderate) Interacting Members Capmatinib, Ciprofloxacin (Systemic), Deferasirox, Enoxacin, Givosiran, Methoxsalen (Systemic), Mexiletine, Rucaparib, Stiripentol, Thiabendazole, Vemurafenib | CYP1A2 | Vomiting | D: Consider therapy modification | Reliability Rating Excellent | Severity Major | Due to the potential severity of theophylline toxicity, consider avoiding the concomitant use of theophylline derivatives and moderate CYP1A2 inhibitors. If coadministration is necessary, monitor for increased theophylline serum concentrations and toxicities (eg, agitation, headache, tachycardia, vomiting, seizures) when combined. Theophylline dose reductions will likely be required. | CYP1A2 Inhibitors (Moderate) may increase the serum concentration of Theophylline Derivatives. |
| Theophylline Derivatives / CYP1A2 Inhibitors (Weak) | TheophAcebrophylline, Aminophylline, Dyphylline, Theophyllineylline Derivatives | CYP1A2 Inhibitors (Weak) Interacting Members Acyclovir (Systemic), Caffeine, Cannabidiol, Cimetidine, Cola-Containing Drinks, Dipyrone, Disulfiram, Elagolix, Estradiol, and Norethindrone, Estradiol (Systemic), Estradiol (Topical), Estrogens (Conjugated A/Synthetic), Estrogens (Conjugated/Equine, Systemic), Estrogens (Conjugated/Equine, Topical), Ethinyl Estradiol, Glecaprevir and Pibrentasvir, Interferon Alfa-2b, Kola Nut, Mestranol, Obeticholic Acid, Osilodrostat, Pefloxacin, Peginterferon Alfa-2a, Peginterferon Alfa-2b, Pipemidic Acid, Propafenone, Propranolol, Simeprevir, Ticlopidine, ValACYclovir, Verapamil, Zileuton | CYP1A2 | Vomiting | C: Monitor therapy | Reliability Rating Excellent | Severity Moderate | Monitor for increased theophylline serum concentrations and toxicities (eg, agitation, headache, tachycardia, vomiting) when theophylline derivatives are combined with weak CYP1A2 inhibitors. Theophylline dose reductions may be required. | CYP1A2 Inhibitors (Weak) may increase the serum concentration of Theophylline Derivatives. |
| Theophylline Derivatives / CYP1A2 Inhibitors (Strong) | TheophyAcebrophylline, Aminophylline, Dyphylline, Theophyllinelline Derivatives | CYP1A2 Inhibitors (Strong) Interacting Members FluvoxaMINE Exception Viloxazine | CYP1A2 | Vomiting | D: Consider therapy modification | Reliability Rating Excellent | Severity Major | Due to the potential severity of theophylline toxicity, consider avoiding the concomitant use of theophylline derivatives and strong CYP1A2 inhibitors. If coadministration is necessary, consider an empiric theophylline dose reduction to one-third of the original theophylline dose. Monitor for increased theophylline serum concentrations and toxicities (eg, agitation, headache, tachycardia, vomiting, seizures) when combined.  See separate drug interaction monographs for drugs listed as ‘Exceptions’ below. | CYP1A2 Inhibitors (Strong) may increase the serum concentration of Theophylline Derivatives. |
| Valbenazine / CYP2D6 Inhibitors (Strong) | Valbenazine | CYP2D6 Inhibitors (Strong) Interacting Members BuPROPion, Dacomitinib, FLUoxetine, PARoxetine, QuiNIDine, Quinidine (Non-Therapeutic), Tipranavir | CYP2D6 | Vomiting | D: Consider therapy modification | Reliability Rating Good | Severity Moderate | Reduce the valbenazine dose to 40 mg once daily when valbenazine is combined with a strong CYP2D6 inhibitor. Monitor for increased valbenazine effects/toxicities (eg, QT prolongation, somnolence, restlessness, arthralgia, vomiting) with any use of this combination. | CYP2D6 Inhibitors (Strong) may increase serum concentrations of the active metabolite(s) of Valbenazine. |
| Cannabis / CYP2C9 Inhibitors (Moderate) | Cannabis | CYP2C9 Inhibitors (Moderate) Interacting Members Fluconazole, MiFEPRIStone, 尼替西农 | CYP2C9 | Dizziness | C: Monitor therapy | Reliability Rating Good | Severity Moderate | Monitor patients who use cannabis in combination with moderate CYP2C9 inhibitors closely for enhanced effects of tetrahydrocannabinol (THC) (eg, cognitive effects, sedation, dizziness, tachycardia). No significant interaction has been described, or is expected, between moderate CYP2C9 inhibitors and cannabis strains/products/uses that do not introduce substantial systemic THC concentrations. | CYP2C9 Inhibitors (Moderate) may increase the serum concentration of Cannabis. More specifically, tetrahydrocannabinol serum concentrations may be increased. |
| Cannabis / CYP3A4 Inhibitors (Strong) | Cannabis | CYP3A4 Inhibitors (Strong) Interacting Members Atazanavir, Ceritinib, Clarithromycin, Cobicistat, Darunavir, Idelalisib, Indinavir, Itraconazole, Ketoconazole (Systemic), Lonafarnib, Lopinavir, MiFEPRIStone, Nefazodone, Nelfinavir, Ombitasvir, Paritaprevir, and Ritonavir, Ombitasvir, Paritaprevir, Ritonavir, and Dasabuvir, Posaconazole, Ritonavir, Saquinavir, Telithromycin, Tucatinib, Voriconazole | CYP3A4 | Dizziness | C: Monitor therapy | Reliability Rating Good | Severity Moderate | Monitor patients who use cannabis in combination with strong CYP3A4 inhibitors closely for enhanced effects of tetrahydrocannabinol (THC eg, cognitive effects, sedation, dizziness, tachycardia) and cannabidiol (CBD eg, muscle relaxant effects). No significant interaction has been described, or is expected, between strong CYP3A4 inhibitors and cannabis strains/products/uses that do not introduce substantial systemic THC or CBD concentrations. | CYP3A4 Inhibitors (Strong) may increase the serum concentration of Cannabis. More specifically, tetrahydrocannabinol and cannabidiol serum concentrations may be increased. |
| Dronabinol / CYP2C9 Inhibitors (Moderate) | Dronabinol | CYP2C9 Inhibitors (Moderate) Interacting Members Fluconazole, 尼替西农 Exception MiFEPRIStone | CYP2C9 | Dizziness | C: Monitor therapy | Reliability Rating Good | Severity Moderate | Monitor patients who use dronabinol in combination with moderate CYP2C9 inhibitors closely for enhanced dronabinol effects (eg, cognitive effects, sedation, dizziness, tachycardia). See separate drug interaction monographs for drugs listed as 'Exceptions' below. | CYP2C9 Inhibitors (Moderate) may increase the serum concentration of Dronabinol. |
| Dronabinol / CYP3A4 Inhibitors (Strong) | Dronabinol | CYP3A4 Inhibitors (Strong) Interacting Members Atazanavir, Ceritinib, Clarithromycin, Cobicistat, Darunavir, Idelalisib, Indinavir, Itraconazole, Ketoconazole (Systemic), Lonafarnib, Lopinavir, MiFEPRIStone, Nefazodone, Nelfinavir, Ombitasvir, Paritaprevir, and Ritonavir, Ombitasvir, Paritaprevir, Ritonavir, and Dasabuvir, Posaconazole, Ritonavir, Saquinavir, Telithromycin, Tucatinib, Voriconazole | CYP3A4 | Dizziness | C: Monitor therapy | Reliability Rating Good | Severity Moderate | Monitor patients who use dronabinol in combination with strong CYP3A4 inhibitors closely for enhanced effects of dronabinol (eg, cognitive effects, sedation, dizziness, tachycardia). | CYP3A4 Inhibitors (Strong) may increase the serum concentration of Dronabinol. |
| Tetrahydrocannabinol / CYP2C9 Inhibitors (Moderate) | Tetrahydrocannabinol | CYP2C9 Inhibitors (Moderate) Interacting Members Fluconazole, 尼替西农 Exception MiFEPRIStone | CYP2C9 | Dizziness | C: Monitor therapy | Reliability Rating Good | Severity Moderate | Monitor patients who use tetrahydrocannabinol (THC) in combination with moderate CYP2C9 inhibitors closely for enhanced THC effects (eg, cognitive effects, sedation, dizziness, tachycardia). See separate drug interaction monographs for drugs listed as 'Exceptions' below. | CYP2C9 Inhibitors (Moderate) may increase the serum concentration of Tetrahydrocannabinol. |
| Tetrahydrocannabinol / CYP3A4 Inhibitors (Strong) | Tetrahydrocannabinol | CYP3A4 Inhibitors (Strong) Interacting Members Atazanavir, Ceritinib, Clarithromycin, Cobicistat, Darunavir, Idelalisib, Indinavir, Itraconazole, Ketoconazole (Systemic), Lonafarnib, Lopinavir, MiFEPRIStone, Nefazodone, Nelfinavir, Ombitasvir, Paritaprevir, and Ritonavir, Ombitasvir, Paritaprevir, Ritonavir, and Dasabuvir, Posaconazole, Ritonavir, Saquinavir, Telithromycin, Tucatinib, Voriconazole | CYP3A4 | Dizziness | C: Monitor therapy | Reliability Rating Good | Severity Moderate | Monitor patients who use tetrahydrocannabinol (THC) in combination with strong CYP3A4 inhibitors closely for enhanced effects of THC (eg, cognitive effects, sedation, dizziness, tachycardia). | CYP3A4 Inhibitors (Strong) may increase the serum concentration of Tetrahydrocannabinol. |
| Tetrahydrocannabinol and Cannabidiol / CYP2C9 Inhibitors (Moderate) | Tetrahydrocannabinol and Cannabidiol | CYP2C9 Inhibitors (Moderate) Interacting Members Fluconazole, 尼替西农 Exception MiFEPRIStone | CYP2C9 | Dizziness | C: Monitor therapy | Reliability Rating Good | Severity Moderate | Monitor patients who use tetrahydrocannabinol (THC) in combination with moderate CYP2C9 inhibitors closely for enhanced THC effects (eg, cognitive effects, sedation, dizziness, tachycardia). See separate drug interaction monographs for drugs listed as 'Exceptions' below. | CYP2C9 Inhibitors (Moderate) may increase the serum concentration of Tetrahydrocannabinol and Cannabidiol. Specifically, concentrations of tetrahydrocannabinol may be increased. |
| Zuclopenthixol / CYP2D6 Inhibitors (Strong) | Zuclopenthixol | CYP2D6 Inhibitors (Strong) Interacting Members BuPROPion, Dacomitinib, FLUoxetine, PARoxetine, QuiNIDine, Quinidine (Non-Therapeutic), Tipranavir | CYP2D6 | Dizziness | C: Monitor therapy | Reliability Rating Good | Severity Moderate | Monitor for increased zuclopenthixol effects and toxicities (eg, drowsiness, extrapyramidal symptoms, dizziness, dry mouth) when combined with strong CYP2D6 inhibitors. | CYP2D6 Inhibitors (Strong) may increase the serum concentration of Zuclopenthixol. |
| CarBAMazepine / CYP3A4 Inhibitors (Moderate) | CarBAMazepine | CYP3A4 Inhibitors (Moderate) Interacting Members Aprepitant, Berotralstat, Conivaptan, Crizotinib, Dronedarone, Duvelisib, Erythromycin (Systemic), Fedratinib, Fluconazole, Fosamprenavir, Fosnetupitant, Imatinib, Isavuconazonium Sulfate, Lefamulin, Letermovir, Netupitant, Nilotinib, Ribociclib, Schisandra Exceptions DilTIAZem, Grapefruit Juice, Verapamil | CYP3A4 | Dizziness | C: Monitor therapy | Reliability Rating Good | Severity Moderate | Monitor for increased carbamazepine levels and toxicity (eg, ataxia, drowsiness, vertigo, diplopia) during coadministration with moderate CYP3A4 inhibitors. Carbamazepine dose reductions may be required. See separate drug interaction monographs for drugs listed as ‘Exceptions’ below. | CYP3A4 Inhibitors (Moderate) may increase the serum concentration of CarBAMazepine. |
| CarBAMazepine / CYP3A4 Inhibitors (Weak) | CarBAMazepine | CYP3A4 Inhibitors (Weak) Interacting Members ALPRAZolam, Amiodarone, AmLODIPine, Berberine, Bicalutamide, Bitter Orange, Chlorzoxazone, Cilostazol, Cimetidine, Ciprofloxacin (Systemic), Clotrimazole (Oral), CycloSPORINE (Systemic), Danazol, Everolimus, FluvoxaMINE, Fosaprepitant, Givosiran, Goldenseal, Grazoprevir, Idebenone, Iloperidone, Istradefylline, Ivacaftor, Lapatinib, Larotrectinib, Levamlodipine, Lomitapide, Lurasidone, Osilodrostat, Palbociclib, PAZOPanib, Peppermint, Piperaquine, Propiverine, Propofol, QuiNIDine, Quinupristin and Dalfopristin, Ranolazine, Resveratrol, Roxithromycin, Rucaparib, Selpercatinib, Tofisopam, Viloxazine, Voxelotor Exceptions Delavirdine, Glecaprevir and Pibrentasvir, Isoniazid | CYP3A4 | Dizziness | C: Monitor therapy | Reliability Rating Fair: Existing data/reports are inconsistent | Severity Moderate | Monitor for increased carbamazepine levels and toxicity (eg, ataxia, drowsiness, vertigo, diplopia) during coadministration with weak CYP3A4 inhibitors. Carbamazepine dose reductions may be required. See separate drug interaction monographs for drugs listed as ‘Exceptions’ below. | CYP3A4 Inhibitors (Weak) may increase the serum concentration of CarBAMazepine. |
| CarBAMazepine / CYP3A4 Inhibitors (Strong) | CarBAMazepine | CYP3A4 Inhibitors (Strong) Interacting Members Atazanavir, Ceritinib, Darunavir, Idelalisib, Indinavir, Itraconazole, Ketoconazole (Systemic), Lonafarnib, MiFEPRIStone, Nelfinavir, Ombitasvir, Paritaprevir, and Ritonavir, Ombitasvir, Paritaprevir, Ritonavir, and Dasabuvir, Posaconazole, Ritonavir, Saquinavir, Telithromycin, Tucatinib, Voriconazole Exceptions Clarithromycin, Cobicistat, Lopinavir, Nefazodone | CYP3A4 | Dizziness | C: Monitor therapy | Reliability Rating Good | Severity Moderate | Monitor for increased carbamazepine levels and toxicity (eg, ataxia, drowsiness, vertigo, diplopia) during coadministration with strong CYP3A4 inhibitors. Carbamazepine dose reductions may be required. See separate drug interaction monographs for drugs listed as ‘Exceptions’ below. | CYP3A4 Inhibitors (Strong) may increase serum concentrations of the active metabolite(s) of CarBAMazepine. CYP3A4 Inhibitors (Strong) may increase the serum concentration of CarBAMazepine. |
| Lonafarnib / CYP3A4 Inhibitors (Strong) | Lonafarnib | CYP3A4 Inhibitors (Strong) Interacting Members Atazanavir, Ceritinib, Clarithromycin, Cobicistat, Darunavir, Idelalisib, Indinavir, Itraconazole, Ketoconazole (Systemic), Lonafarnib, Lopinavir, MiFEPRIStone, Nefazodone, Nelfinavir, Ombitasvir, Paritaprevir, and Ritonavir, Ombitasvir, Paritaprevir, Ritonavir, and Dasabuvir, Posaconazole, Ritonavir, Saquinavir, Telithromycin, Tucatinib, Voriconazole | CYP3A4 | Dizziness | X: Avoid combination | Reliability Rating Good | Severity Moderate | Do not use lonafarnib with strong CYP3A4 inhibitors. The lonafarnib prescribing information lists this combination as contraindicated. | CYP3A4 Inhibitors (Strong) may increase the serum concentration of Lonafarnib. |
| Lonafarnib / CYP3A4 Inhibitors (Weak) | Lonafarnib | CYP3A4 Inhibitors (Weak) Interacting Members ALPRAZolam, Amiodarone, AmLODIPine, Berberine, Bicalutamide, Chlorzoxazone, Cilostazol, Cimetidine, Ciprofloxacin (Systemic), Clotrimazole (Oral), CycloSPORINE (Systemic), Danazol, Delavirdine, Everolimus, FluvoxaMINE, Fosaprepitant, Givosiran, Glecaprevir and Pibrentasvir, Goldenseal, Grazoprevir, Idebenone, Iloperidone, Isoniazid, Istradefylline, Ivacaftor, Lapatinib, Larotrectinib, Levamlodipine, Lomitapide, Lurasidone, Osilodrostat, Palbociclib, PAZOPanib, Peppermint, Piperaquine, Propiverine, Propofol, QuiNIDine, Quinupristin and Dalfopristin, Ranolazine, Resveratrol, Roxithromycin, Rucaparib, Selpercatinib, Tofisopam, Viloxazine, Voxelotor Exception Bitter Orange | CYP3A4 | Dizziness | D: Consider therapy modification | Reliability Rating Fair: Reported in the prescribing information | Severity Moderate | Avoid the concurrent use of lonafarnib with weak CYP3A4 inhibitors. If concurrent use is unavoidable, reduce the lonafarnib dose to 115 mg/m2 or continue lonafarnib at a dose of 115 mg/m2. With any combined use, monitor patient closely for evidence of arrhythmia, syncope, palpitations, or similar effects. Bitter orange is specifically contraindicated with lonafarnib, and that interaction is listed as an exception here because it is discussed in a separate interaction monograph. | CYP3A4 Inhibitors (Weak) may increase the serum concentration of Lonafarnib. |
| Avanafil / CYP3A4 Inhibitors (Moderate) | Avanafil | CYP3A4 Inhibitors (Moderate) Interacting Members Aprepitant, Berotralstat, Conivaptan, Crizotinib, DilTIAZem, Dronedarone, Duvelisib, Erythromycin (Systemic), Fedratinib, Fluconazole, Fosamprenavir, Fosnetupitant, Grapefruit Juice, Imatinib, Isavuconazonium Sulfate, Lefamulin, Letermovir, Netupitant, Nilotinib, Ribociclib, Schisandra, Verapamil | CYP3A4 | Dizziness | D: Consider therapy modification | Reliability Rating Good | Severity Moderate | The maximum avanafil dose is 50 mg per 24-hour period when used together with a moderate CYP3A4 inhibitor. Patients receiving such a combination should also be monitored more closely for evidence of adverse effects (eg, hypotension, syncope, priapism). | CYP3A4 Inhibitors (Moderate) may increase the serum concentration of Avanafil. |
| Lonafarnib / CYP3A4 Inhibitors (Weak) | Lonafarnib | CYP3A4 Inhibitors (Weak) Interacting Members ALPRAZolam, Amiodarone, AmLODIPine, Berberine, Bicalutamide, Chlorzoxazone, Cilostazol, Cimetidine, Ciprofloxacin (Systemic), Clotrimazole (Oral), CycloSPORINE (Systemic), Danazol, Delavirdine, Everolimus, FluvoxaMINE, Fosaprepitant, Givosiran, Glecaprevir and Pibrentasvir, Goldenseal, Grazoprevir, Idebenone, Iloperidone, Isoniazid, Istradefylline, Ivacaftor, Lapatinib, Larotrectinib, Levamlodipine, Lomitapide, Lurasidone, Osilodrostat, Palbociclib, PAZOPanib, Peppermint, Piperaquine, Propiverine, Propofol, QuiNIDine, Quinupristin and Dalfopristin, Ranolazine, Resveratrol, Roxithromycin, Rucaparib, Selpercatinib, Tofisopam, Viloxazine, Voxelotor Exception Bitter Orange | CYP3A4 | Dizziness | D: Consider therapy modification | Reliability Rating Fair: Reported in the prescribing information | Severity Moderate | Avoid the concurrent use of lonafarnib with weak CYP3A4 inhibitors. If concurrent use is unavoidable, reduce the lonafarnib dose to 115 mg/m2 or continue lonafarnib at a dose of 115 mg/m2. With any combined use, monitor patient closely for evidence of arrhythmia, syncope, palpitations, or similar effects. Bitter orange is specifically contraindicated with lonafarnib, and that interaction is listed as an exception here because it is discussed in a separate interaction monograph. | CYP3A4 Inhibitors (Weak) may increase the serum concentration of Lonafarnib. |
| Nefazodone / Opioid Agonists (metabolized by CYP3A4 and CYP2D6) | Nefazodone | Opioid Agonists (metabolized by CYP3A4 and CYP2D6) Interacting Members Benzhydrocodone, Codeine, Dihydrocodeine, HYDROcodone, Oliceridine | CYP3A4 and CYP2D6 | serotonin syndrome | C: Monitor therapy | Reliability Rating Good | Severity Moderate | Monitor patients for increased opioid toxicities, including fatal respiratory depression, if combined with nefazodone. Monitor patients at frequent intervals and consider opioid agonist dose reductions until stable drug effects are achieved. Additionally, monitor for signs and symptoms of serotonin syndrome/serotonin toxicity (eg, hyperreflexia, clonus, hyperthermia, diaphoresis, tremor, autonomic instability, mental status changes) when these drugs are combined. Patients with other risk factors (eg, higher drug concentrations/doses, greater numbers of serotonergic agents) are likely at greater risk for these potentially life-threatening toxicities. | Opioid Agonists (metabolized by CYP3A4 and CYP2D6) may enhance the serotonergic effect of Nefazodone. This could result in serotonin syndrome. Nefazodone may increase the serum concentration of Opioid Agonists (metabolized by CYP3A4 and CYP2D6). |

A drug from Members of Drug 1 and a drug from Members of Drug 2 make up the CYP-mediated DDI pairings.
